# Transcriptional Biomarkers of Differentially Detectable Mycobacterium tuberculosis in Patient Sputum

**DOI:** 10.1128/mbio.02701-22

**Published:** 2022-11-03

**Authors:** Kayvan Zainabadi, Kohta Saito, Saurabh Mishra, Kathleen Frances Walsh, Laurent Daniel Mathurin, Stalz Charles Vilbrun, Oksana Ocheretina, Jean William Pape, Daniel W. Fitzgerald, Carl F. Nathan, Myung Hee Lee

**Affiliations:** a Center for Global Health, Weill Cornell Medicine, New York, New York, USA; b Department of Medicine, Division of Infectious Diseases, Weill Cornell Medicine, New York, New York, USA; c Department of Microbiology & Immunology, Weill Cornell Medicine, New York, New York, USA; d Department of Medicine, Division of General Internal Medicine, Weill Cornell Medicine, New York, New York, USA; e Les Centres GHESKIO, Port-au-Prince, Haiti; Rutgers New Jersey Medical School; New York University School of Medicine

**Keywords:** *Mycobacterium tuberculosis*, clinical methods, diagnostics, differentially detectable bacteria, molecular methods, multidrug resistance, persistence, viable but nonculturable

## Abstract

Certain populations of Mycobacterium tuberculosis go undetected by standard diagnostics but can be enumerated using limiting dilution assays. These differentially detectable M. tuberculosis (DD M. tuberculosis) populations may have relevance for persistence due to their drug tolerance. It is unclear how well DD M. tuberculosis from patients is modeled by a recently developed *in vitro* model in which M. tuberculosis starved in phosphate-buffered saline is incubated with rifampin to produce DD M. tuberculosis (the PBS-RIF model). This study attempted to answer this question. We selected 14 genes that displayed differential expression in the PBS-RIF model and evaluated their expression in patient sputa containing various proportions of DD M. tuberculosis. The expression of 12/14 genes correlated with the relative abundance of DD M. tuberculosis in patient sputa. Culture filtrate (CF), which promotes recovery of DD M. tuberculosis from certain patient sputa, improved these correlations in most cases. The gene whose reduced expression relative to M. tuberculosis 16S rRNA showed the greatest association with the presence and relative abundance of DD M. tuberculosis in patient sputa, *icl1*, was recently shown to play a functional role in restraining DD M. tuberculosis formation in the PBS-RIF model. Expression of *icl1*, combined with two additional DD M. tuberculosis-related genes, showed strong performance for predicting the presence or absence of DD M. tuberculosis in patient sputa (receiver operating characteristic [ROC] area under the curve [AUC] = 0.88). Thus, the *in vitro* DD M. tuberculosis model developed by Saito et al. (K. Saito, T. Warrier, S. Somersan-Karakaya, L. Kaminski, et al., Proc Natl Acad Sci U S A 114:E4832–E4840, 2017, https://doi.org/10.1073/pnas.1705385114) bears a resemblance to DD M. tuberculosis found in tuberculosis (TB) patients, and DD M. tuberculosis transcriptional profiles may be useful for monitoring DD M. tuberculosis populations in patient sputum.

## INTRODUCTION

Tuberculosis (TB) continues to be a leading cause of morbidity and mortality across the world, particularly in developing countries, where 95% of deaths occur (1). Currently used diagnostics are effective for detecting Mycobacterium tuberculosis infection in treatment-naive individuals but are poor at monitoring treatment response during the course of therapy (2, 3). As a consequence, patients with drug-sensitive TB are required to adhere to a 6-month treatment regimen, even though a majority are known to be cured after 2 months (3).

The gold standard method of enumerating M. tuberculosis is by culturing sputum on solid agar and counting the number of CFU. CFU and other standard culture-based methods are unable to detect all populations of M. tuberculosis present in sputa (4–15). Viable but differentially detectable/culturable M. tuberculosis (DD M. tuberculosis) can be detected and enumerated by the most probable number method using limiting dilution assays (MPN-LD). The excess viable M. tuberculosis bacteria identified by MPN-LD versus CFU are defined as the DD M. tuberculosis population. In certain cases, DD M. tuberculosis is recoverable only when the medium for the MPN-LD assay is supplemented with culture filtrate (CF), that is, cell-free medium from a growing culture of a laboratory strain of M. tuberculosis (12–16). DD M. tuberculosis can at times constitute the majority of viable M. tuberculosis bacteria in the sputa of a majority of TB patients, particularly after initiation of first-line treatment (11–15). These findings may have implications for M. tuberculosis persistence as DD M. tuberculosis is profoundly tolerant to the drugs used in anti-TB combination chemotherapy (11, 17, 18). The presence of DD M. tuberculosis may help explain the long-standing observation that lung homogenates of surgical specimens from TB patients can lack detectable M. tuberculosis based on culture but still be capable of causing infection in experimental animals (19).

An *in vitro* model of DD M. tuberculosis was recently developed in which DD M. tuberculosis is generated by first starving M. tuberculosis of nutrients in phosphate-buffered saline and then incubating cells with a high dose of rifampin (here referred to as the PBS-RIF model) (11). Characterization of this model has revealed that intermediate levels of oxidative damage may contribute to DD M. tuberculosis generation (20). RNA sequencing (RNA-seq) experiments with the PBS-RIF model have also uncovered gene expression changes associated with DD M. tuberculosis (20). Such gene expression profiles could serve as potential biomarkers for detection of DD M. tuberculosis. However, it is unknown how well the PBS-RIF model corresponds to DD M. tuberculosis in patients.

In this study, we attempted to address this question. Using stringent selection criteria, we identified 14 genes that were differentially expressed in the PBS-RIF model and tested their expression in TB patient sputa containing various proportions of DD M. tuberculosis. Our observation that the expression of most of these genes show a significant correlation with the relative abundance of DD M. tuberculosis found in patient sputa provides evidence that the PBS-RIF model recapitulates aspects of DD M. tuberculosis biology found in patients. We further explored whether some of these transcriptional changes could be utilized to develop tools for detection of DD M. tuberculosis in patient sputa.

## RESULTS

### Selection criteria for DD M. tuberculosis candidate genes from the PBS-RIF model.

A total of 253 genes showed a ≥6-fold change in expression from two independent RNA-seq experiments comparing M. tuberculosis from the PBS-RIF model to M. tuberculosis from PBS-dimethyl sulfoxide (DMSO) controls (20). From these, we selected 23 genes (here referred to as DD M. tuberculosis candidate genes) for further evaluation based on their average fold change in expression based on the aforementioned RNA-seq experiments and whether additional gene family members showed similar changes, their level of expression in sputum as determined by Walter et al. (21), and any ascribed role in M. tuberculosis persistence and/or other DD M. tuberculosis models (22, 23) (see Table S1 in the supplemental material). For instance, 10 of 13 downregulated DD M. tuberculosis candidate genes had been found downregulated in an independent *in vitro* model of DD M. tuberculosis that uses potassium starvation and a lower dose of rifampin (22), and 8 of 13 had been implicated in models of M. tuberculosis persistence (23) (Table S1).

10.1128/mbio.02701-22.6 TABLE S1 List of all M. tuberculosis genes that showed ≥6-fold change in expression between DD M. tuberculosis-positive and -negative cultures. Average fold change from two independent RNA-seq experiments from the *in vitro* DD M. tuberculosis model is presented in column C, fold change by RNA-seq in an independent DD M. tuberculosis model is presented in column D, association in models of M. tuberculosis persistence is presented in column E, and ranked expression by high-throughput qRT-PCR in patient sputum before and after 2 weeks of first-line treatment is presented in columns F and G. Primer sequences are provided in the “Primers” tab. Download Table S1, XLSX file, 0.1 MB. Copyright © 2022 Zainabadi et al. 2022 Zainabadi et al. https://creativecommons.org/licenses/by/4.0/ This content is distributed under the terms of the Creative Commons Attribution 4.0 International license.

For each of the 23 genes, we designed four primers (to yield a total of four possible amplicons) to identify the primer set that yielded the lowest cycle threshold (*C_T_*) value. Next, we ranked the expression of the 23 genes by quantitative reverse transcription-PCR (qRT-PCR) using RNA from pooled pretreatment sputa derived from our patient population (Table S2). To increase the chances of detecting gene expression after initiation of treatment (when total M. tuberculosis counts drop precipitously), we chose to study only the most highly expressed genes. This resulted in a final list of 10 downregulated and 4 upregulated DD M. tuberculosis candidate genes (Table 1).

**TABLE 1:** List of the 14 DD M. tuberculosis candidate genes chosen for this study that in a prior report (20) were found downregulated or upregulated in expression in the PBS-RIF model in comparison to PBS-DMSO controls

DD M. tuberculosis candidate gene	Gene product/function^*g*^	Functional category	Avg fold Δexpression PBS-RIF DD M. tuberculosis^*a*^^,^^*b*^	Avg fold Δexpression −K^+^ RIF DD M. tuberculosis^*c*^	Implicated in models of M. tuberculosis persistence?^*d*^	Avg *C_T_* (day 0 pooled sputum)^*e*^	Ranked expression 0–100 (day 0 sputum)^*f*^
Downregulated							
*icl1* (Rv0467)	Isocitrate lyase/involved in glyoxylate bypass in TCA cycle	Intermediary metabolism and respiration	−45.9^*h*^	−3.0	Yes	20.1	99.6
*carD* (Rv3583c)	RNA-Pol binding transcription factor	Regulatory proteins (transcriptional)	−22.0^*h*^	−1.4		21.4	99.2
*vapB10* (Rv1398c)	Possible antitoxin	Virulence, detoxification, adaptation	−29.4^*h*^	−2.5	Yes	20.8	99.2
*ppsA* (Rv2931)	Phenol phthiocerol synthesis type I polyketide synthase	Lipid metabolism	−13.9^*h*^	−1.8	Yes	21.4	95.5
*hspX* (Rv2031c)	Heat shock protein (α-crystallin homolog)	Virulence, detoxification, adaptation	−10.5	1.0	Yes	20.4	92.3
Rv1738	Similar to bacterial hibernation factors	Conserved hypotheticals	−13.8	6.0	Yes	21.9	98.5
*tatA* (Rv2094c)	Sec-independent protein translocase	Cell wall and cell processes	−14.0	−9.7		20.6	98.1
*whiB1* (Rv3219)	NO-responsive transcription factor	Regulatory proteins (transcriptional)	−11.3^*h*^	−4.2	Yes	24.0	98.6
*pks15* (Rv2947c)	Probable polyketide synthase	Lipid metabolism	−16.0^*h*^	2.6		25.5	60
*lldD2* (Rv1872c)	Possible l-lactate dehydrogenase (cytochrome)	Intermediary metabolism and respiration	−10.0^*h*^	−3.6	Yes	24.0	95.1
Upregulated							
*arsC* (Rv2643)	Probable arsenic-transport integral membrane protein	Cell wall and cell processes	7.7	2.6	Yes	25.2	56.5
*lpqX* (Rv1228)	Probable lipoprotein	Cell wall and cell processes	17.2^*h*^	8.4		27.1	72.8
*ugpC* (Rv2832c)	Probable *sn*-glycerol-3-phosphate transport ABC transporter	Cell wall and cell processes	11.6^*h*^	9.6		25.7	32.3
*rpfE* (Rv2450c)	Probable resuscitation-promoting factor	Cell wall and cell processes	11.2	−2.3		27.1	32.3

a Comparing DD M. tuberculosis positive versus negative cultures from Saito et al. (20).

b The PBS used in this model contains potassium.

c Comparing DD M. tuberculosis positive versus negative cultures from Ignatov et al. (22).

d Torrey et al. (23).

e This study.

f Walter et al. (21) (100 represents highest expressed gene in sputum based on high-throughput qRT-PCR).

g Abbreviations: TCA, tricarboxylic acid cycle; Pol, polymerase; NO, nitric oxide; ABC, ATP-binding cassette.

h Other putative gene/operon family members show similar changes in expression.

10.1128/mbio.02701-22.7 TABLE S2 qRT-PCR cycle threshold (*C_T_*) values for the 23 DD M. tuberculosis candidate genes were obtained using pooled pretreatment sputa derived from the patient population used in this study and compared to the ranked expression of each gene as determined by high-throughput qRT-PCR based on the work of Walter et al. (N. D. Walter, G. M. Dolganov, B. J. Garcia, W. Worodria, et al., J Infect Dis 212:990–998, 2015, https://doi.org/10.1093/infdis/jiv149). Genes in red are predicted by RNA-seq to decrease in expression with DD M. tuberculosis based on the *in vitro* model, genes in blue are predicted to increase in expression with DD M. tuberculosis, and genes in black were used as references. *, 100 represents the highest-expressed gene in sputum and 0 represents the lowest. Download Table S2, XLSX file, 0.01 MB. Copyright © 2022 Zainabadi et al. 2022 Zainabadi et al. https://creativecommons.org/licenses/by/4.0/ This content is distributed under the terms of the Creative Commons Attribution 4.0 International license.

### Relative expression of DD M. tuberculosis candidate genes in TB patient sputa containing DD M. tuberculosis.

The relative expression of these 14 genes (normalized to M. tuberculosis 16S rRNA) was then assessed using sputa from a published cohort of subjects with drug-sensitive (DS) or drug-resistant (DR) TB (here referred to as the DS and DR cohorts) before and after initiation of first-line (rifampin-containing) or second-line (non-rifampin-containing) treatment regimens, respectively (15). In these subjects, DD M. tuberculosis was present (defined statistically as when the MPN value was more than the upper bound of the 95% confidence interval (CI) of the CFU value) in 29 to 30% of patients’ pretreatment sputa from the two cohorts and in 73% and 27% of patients’ sputa after initiation of treatment from the DS and DR cohorts, respectively. The relative abundance of DD M. tuberculosis (as measured by the MPN/CFU ratio) in the sputa of these subjects varied accordingly, with the highest levels found in sputa from the DS cohort after initiation of treatment (Table 2).

**TABLE 2:** The size and median relative abundance of DD M. tuberculosis in the sputa of the various cohorts used in this study (as determined by the MPN/CFU ratio with or without CF)^*c*^

Cohort	Sample size (*n*)	MPN^Max^/CFU (median [Q1, Q3])	MPN^+CF^/CFU (median [Q1, Q3])	MPN^−CF^/CFU (median [Q1, Q3])
All	62–63^*a*^	1.73 [1.37, 2.94]	1.55 [1.05, 2.39]	1.43 [0.95, 2.39]
All DS	35	1.85 [1.28, 3.64]	1.61 [0.97, 2.97]	1.51 [0.99, 3.07]
All DR	27–28^*a*^	1.61 [1.38, 2.22]	1.49 [1.18, 2.16]	1.37 [0.91, 1.92]
All-D0	36–37^*a*^	1.60 [1.22, 2.21]	1.43 [0.83, 1.73]	1.28 [0.95, 1.90]
All-W2	26	2.25 [1.52, 5.66]	2.17 [1.43, 5.01]	1.86 [1.27, 3.60]
DS-D0	20	1.52 [1.00, 2.22]	1.40 [0.81, 1.56]	1.23 [0.95, 1.83]
DS-W2	15	3.80 [2.02, 6.22]	3.19 [1.88, 5.89]	2.46 [1.62, 5.47]
DR-D0	16–17^*a*^	1.61 [1.38, 2.21]	1.56 [1.10, 1.89]	1.38 [0.96, 1.90]
DR-W2	11	1.55 [1.34, 2.70]	1.43 [1.27, 2.70]	1.36 [0.79, 1.86]
All paired^*b*^	21–22	0.77 [−0.28, 3.86]	0.83 [0.12, 3.67]	0.83 [−0.42, 1.64]
DS-paired^*b*^	13	1.65 [0.73, 4.40]	1.65 [0.73, 5.36]	1.29 [0.81, 4.40]
DR-paired^*b*^	8–9^*a*^	0.35 [−0.71, 0.05]	0.08 [−0.71, 0.42]	−0.52 [−0.61, −0.06]

a Sample size smaller for MPN^+CF^ due to a contamination event.

b Δ[MPN/CFU] values (week 2 versus day 0) are presented.

c Abbreviations: DS, drug sensitive; DR, drug resistant; D0, day 0; W2, week 2; MPN, most probable number; MPN^Max^, the maximum M. tuberculosis count obtained by MPN with or without CF; CF, culture filtrate.

**FIG 1:**
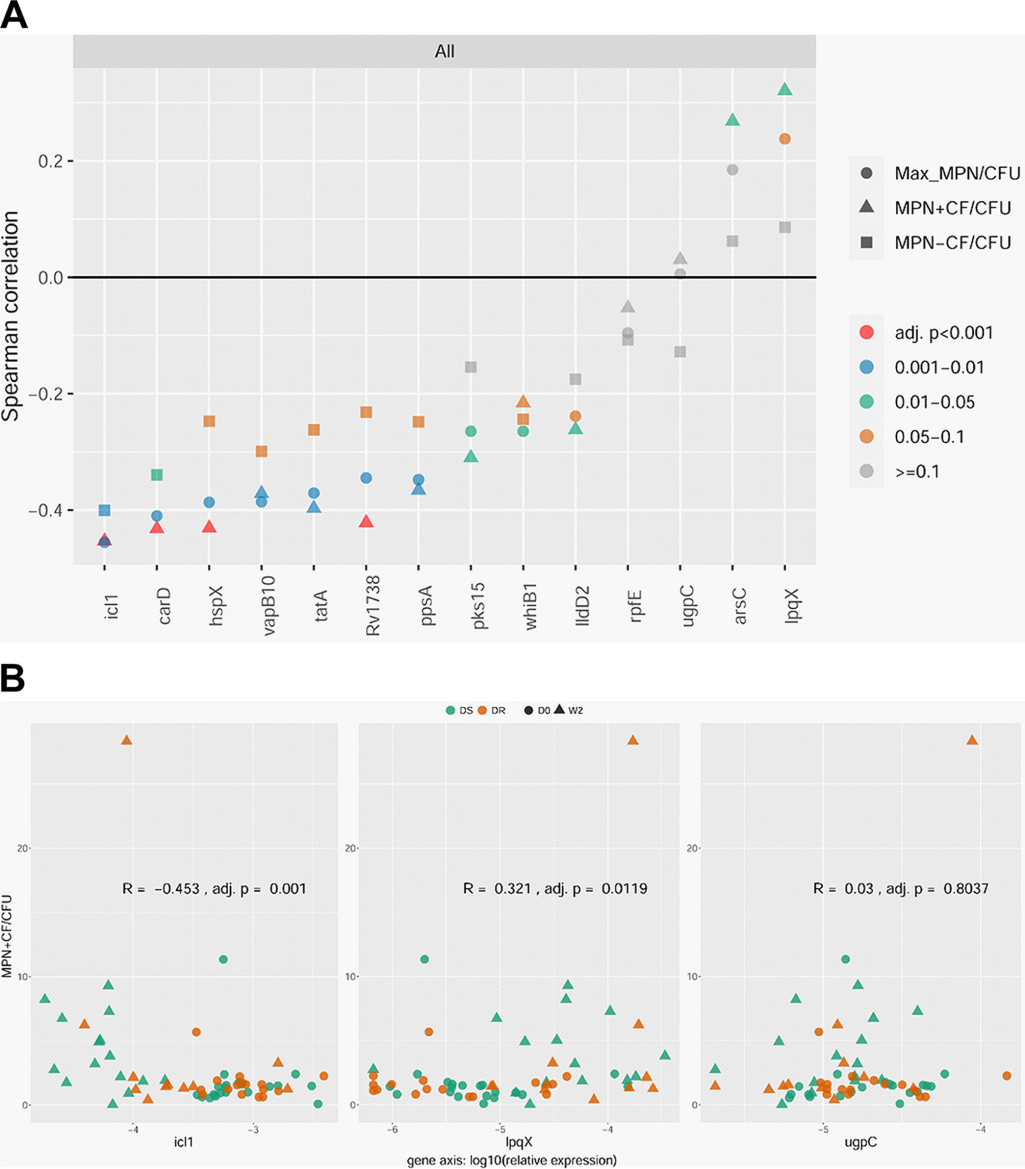
(A) Summary of Spearman correlation coefficients for the relative expression of the 14 DD M. tuberculosis candidate genes with respect to the relative abundance of DD M. tuberculosis in sputa from patients with drug-sensitive (DS) or drug-resistant (DR) TB before and after initiation of therapy. (B) Representative scatterplots for genes with Spearman correlation coefficients that are negative (*icl1*), positive (*lpqX*), or insignificant (*ugpC*) (as determined by the MPN^+CF^/CFU ratio). Data are presented for all samples (*n* = 62 to 63). Complete scatterplots and *R* and *P* values can be found in Fig. S3 and Table S3. D0, day 0; W2, week 2; MPN, most probable number; Max MPN, the maximum M. tuberculosis count obtained by MPN with or without CF; CF, culture filtrate.

We used Spearman rank-based correlation coefficients to measure the strength and direction of the monotonic relationship between relative gene expression and relative abundance of DD M. tuberculosis in sputa using a metric between −1 and 1 (with 0 representing no association and −1 and 1 representing perfect negative and positive association, respectively). When analyzing all sputum samples (that is, sputa from both cohorts at both time points, *n* = 62 to 63), 12 of the 14 DD M. tuberculosis candidate genes showed Spearman correlation coefficients that were statistically significant (adjusted *P* value < 0.05): all 10 candidates found by RNA-seq to be downregulated in the PBS-RIF model showed a significant negative Spearman correlation coefficient, and two of four candidates found to be upregulated by RNA-seq showed a significant positive Spearman correlation coefficient (Fig. 1 and Table S3). The presence of culture filtrate (CF) in the MPN-LD assay, which has been shown to promote recovery of certain DD M. tuberculosis populations (12–16), improved the Spearman correlation coefficient for 11 of the 12 DD M. tuberculosis candidates (Fig. S1 and Table S3). In fact, nine of the 12 DD M. tuberculosis candidates showed a statistically significant Spearman correlation coefficient only when CF was included in the MPN-LD assay.

10.1128/mbio.02701-22.1 FIG S1 The effect of culture filtrate (CF) on the association of relative gene expression with relative DD M. tuberculosis abundance in patient sputa for the 14 DD M. tuberculosis candidate genes. (A) Scatterplots of Spearman correlation coefficients with CF (*y* axis) and without CF (*x* axis). Top panel represents all sputum samples, middle panel represents week 2 sputum samples, and bottom panel represents day 0 sputum samples. The 45-degree straight line represents the null states where Spearman correlation coefficients with DD M. tuberculosis determined by MPN with and without CF are the same. (B) The effect of different DD M. tuberculosis detection methods (Max MPN/CFU, MPN^+CF^/CFU, or MPN^−CF^/CFU) on the correlation of relative gene expression with DD M. tuberculosis relative abundance for the 14 DD M. tuberculosis candidate genes (as depicted by circles or triangles). DD M. tuberculosis candidate genes predicted to be up- or downregulated by RNA-seq are denoted by circle and triangle markers, respectively, and significance (by adjusted *P* value) is depicted by the color red, otherwise black. The height of the markers on the *y* axis represents Spearman correlations, and the *x* axis represents different sputum sample cohorts. Download FIG S1, PDF file, 1.2 MB. Copyright © 2022 Zainabadi et al. 2022 Zainabadi et al. https://creativecommons.org/licenses/by/4.0/ This content is distributed under the terms of the Creative Commons Attribution 4.0 International license.

10.1128/mbio.02701-22.8 TABLE S3 The relative expression of the 14 DD M. tuberculosis candidate genes (normalized to M. tuberculosis 16S rRNA) in relation to the relative abundance of DD M. tuberculosis present in patient sputa (as determined by the MPN/CFU ratio) in various analyses and subanalyses of the two cohorts (DS and DR) both before (D0) and after (W2) initiation of treatment. Adjusted *P* values of <0.05 are highlighted in red; adjusted *P* values of 0.05 to 0.10 are highlighted in blue. MPN, most probable number; MPN^Max^, the maximum M. tuberculosis number obtained by MPN with or without CF; CF, culture filtrate; DS, drug sensitive; DR, drug resistant; D0, day 0; W2, week 2. Download Table S3, DOCX file, 0.05 MB. Copyright © 2022 Zainabadi et al. 2022 Zainabadi et al. https://creativecommons.org/licenses/by/4.0/ This content is distributed under the terms of the Creative Commons Attribution 4.0 International license.

10.1128/mbio.02701-22.3 FIG S3 Summary of Spearman correlation coefficients for the relative gene expression (normalized to M. tuberculosis 16S rRNA) for the 14 DD M. tuberculosis candidate genes with respect to the relative abundance of DD M. tuberculosis (as determined by the Max MPN/CFU, MPN^+CF^/CFU, or MPN^−CF^/CFU ratio) in the sputa of patients with drug-sensitive (DS) or drug-resistant (DR) TB before (D0) and after 2 weeks of (W2) first-line or second-line treatment regimens, respectively. Data for all samples (sputa from both cohorts at both time points) and various subanalyses are presented; red color represents adjusted *P* value of <0.05. Sample sizes for each analysis are as follows: all, *n* = 62 to 63; DS, *n* = 35; DR, *n* = 27 to 28; D0, *n* = 36 to 37; W2, *n* = 26; DS-D0, *n* = 20; DS-W2, *n* = 15; DR-D0, *n* = 16 to 17; DR-W2, *n* = 11. Complete scatterplots and *R* and *P* values can be found below and in Table S3. MPN, most probable number; Max MPN, the maximum M. tuberculosis count obtained by MPN with or without CF; CF, culture filtrate. Download FIG S3, PDF file, 2.9 MB. Copyright © 2022 Zainabadi et al. 2022 Zainabadi et al. https://creativecommons.org/licenses/by/4.0/ This content is distributed under the terms of the Creative Commons Attribution 4.0 International license.

Hierarchical clustering analysis was performed to identify groups of genes that showed similar associations between relative gene expression and relative abundance of DD M. tuberculosis in sputa. This analysis showed that the 4 upregulated DD M. tuberculosis candidate genes formed a distinct cluster separate from the 10 downregulated DD M. tuberculosis candidates (Fig. S2).

10.1128/mbio.02701-22.2 FIG S2 Using the calculated Spearman correlation coefficients from Fig. 1, hierarchical clustering analysis using complete linkage was performed to identify genes that showed similar associations between relative gene expression and DD M. tuberculosis relative abundance in sputa. Download FIG S2, PDF file, 0.4 MB. Copyright © 2022 Zainabadi et al. 2022 Zainabadi et al. https://creativecommons.org/licenses/by/4.0/ This content is distributed under the terms of the Creative Commons Attribution 4.0 International license.

### Diagnostic utility of gene expression profiles for predicting presence or absence of DD M. tuberculosis in sputa.

**FIG 2:**
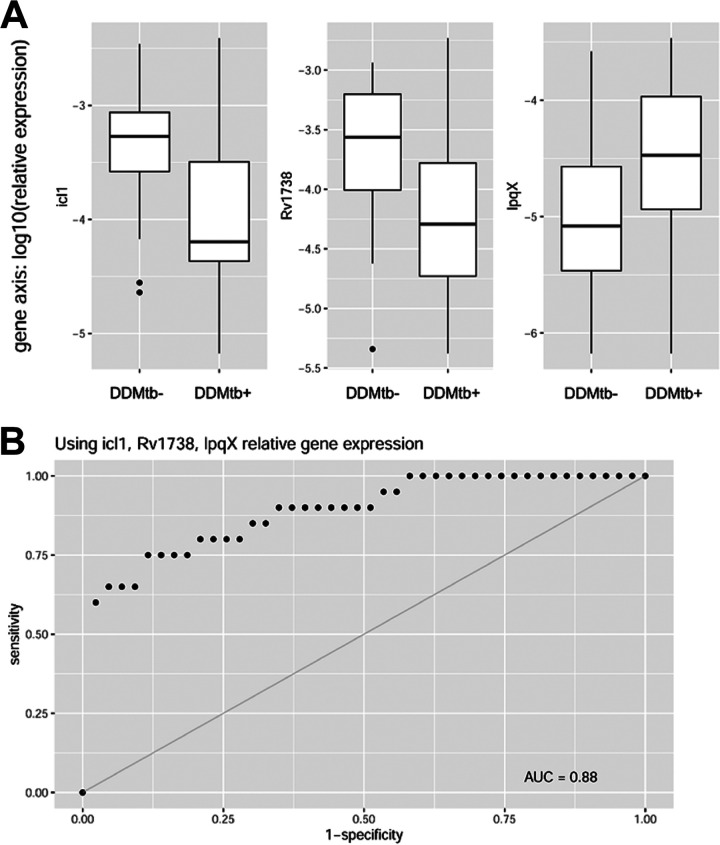
(A) Box plots of relative gene expression for *icl1*, *rv1738*, and *lpqX* show good separation in distributions based on presence or absence of DD M. tuberculosis in sputa. (B) Combining the relative gene expression for these three genes, DD M. tuberculosis presence in sputa is predicted when both *icl1* and *rv1738* expression levels are low and *lpqX* expression is high with an area under the receiver operating characteristic curve (ROC AUC) of 0.88 with a 95% CI of 0.77, 0.99.

The relative expression of certain DD M. tuberculosis candidate genes showed considerable separation in distributions based on whether sputa were DD M. tuberculosis positive or negative (Fig. 2). We therefore evaluated the utility of gene expression profiles as a diagnostic test for the presence of DD M. tuberculosis in sputa. The predictive power of each gene was determined by constructing receiver operating characteristic (ROC) curves and analyzing the area under the curve (AUC), with an AUC of 1 indicating perfect predictive power and an AUC of 0.5 indicating no predictive power. Expression of six of the DD M. tuberculosis candidate genes analyzed individually showed good predictive performance with regard to presence or absence of DD M. tuberculosis in sputa (ROC AUC values > 0.70), with *icl1* showing the best performance (AUC = 0.78 [95% CI = 0.65, 0.92] and 10-fold cross-validation average accuracy of 71.5% [standard deviation = 1.76%] over 500 runs). Predictive power improved when relative expression of a downregulated DD M. tuberculosis candidate gene was combined with one that was upregulated, with *icl1* and *lpqX* representing the best combination of genes (AUC = 0.84 [95% CI = 0.71, 0.97]). This was further improved by including a third gene, *rv1738*, which increased the AUC to 0.88 (95% CI = 0.77, 0.99) (Fig. 2). No further improvements were found with inclusion of additional genes.

10.1128/mbio.02701-22.4 FIG S4 Summary of Spearman correlation coefficients comparing the Δ(relative gene expression) at week 2 versus day 0 for the 14 DD M. tuberculosis candidate genes with respect to Δ(DD M. tuberculosis relative abundance) for paired sputum samples from patients with drug-sensitive (DS) or drug-resistant (DR) TB before and after initiation of first-line or second-line therapy, respectively. All, *n* = 21 to 22; DS, *n* = 13; DR, *n* = 8 to 9. Complete scatterplots and *R* and *P* values can be found below and in Table S4. MPN, most probable number; Max MPN, the maximum M. tuberculosis count obtained by MPN with or without CF; CF, culture filtrate. Download FIG S4, PDF file, 0.8 MB. Copyright © 2022 Zainabadi et al. 2022 Zainabadi et al. https://creativecommons.org/licenses/by/4.0/ This content is distributed under the terms of the Creative Commons Attribution 4.0 International license.

10.1128/mbio.02701-22.9 TABLE S4 Summary of Spearman correlation coefficients comparing the Δ(relative gene expression) at week 2 versus day 0 for the 14 DD M. tuberculosis candidate genes with respect to Δ(DD M. tuberculosis relative abundance) as determined by the Δ(MPN/CFU) ratio for all paired sputum samples or paired sputa from the individual cohorts (DS and DR). *P* values of <0.05 are highlighted in red; *P* values of 0.05 to 0.10 are highlighted in blue. MPN, most probable number; MPN^Max^, the maximum M. tuberculosis number obtained by MPN with or without CF; CF, culture filtrate; DS, drug sensitive; DR, drug resistant. Download Table S4, DOCX file, 0.02 MB. Copyright © 2022 Zainabadi et al. 2022 Zainabadi et al. https://creativecommons.org/licenses/by/4.0/ This content is distributed under the terms of the Creative Commons Attribution 4.0 International license.

### Stratifying data based on cohort or time point.

Next, we assessed whether similar or new associations between relative gene expression and relative DD M. tuberculosis abundance could be found when stratifying data based on cohort (DS versus DR) or time point (before versus after initiation of treatment). Of the 12 DD M. tuberculosis candidate genes that showed significant Spearman correlation coefficients in prior analyses (*n* = 62 to 63), 11 were also significant when analyzing sputa from the DS cohort (*n* = 35) (Fig. S3 and Table S3). For sputa from the DR cohort (*n* = 27 to 28), where sample size and relative abundance of DD M. tuberculosis were both lower (Table 2), the Spearman correlation coefficients for 10 of the 12 genes trended in the same direction but did not reach statistical significance (Fig. S3 and Table S3). When stratifying data based on time point, *icl1* was the only gene whose relative expression showed a significant correlation with the relative abundance of DD M. tuberculosis in sputa from after initiation of treatment from both cohorts (*n* = 26) (Table 3).

**TABLE 3:**
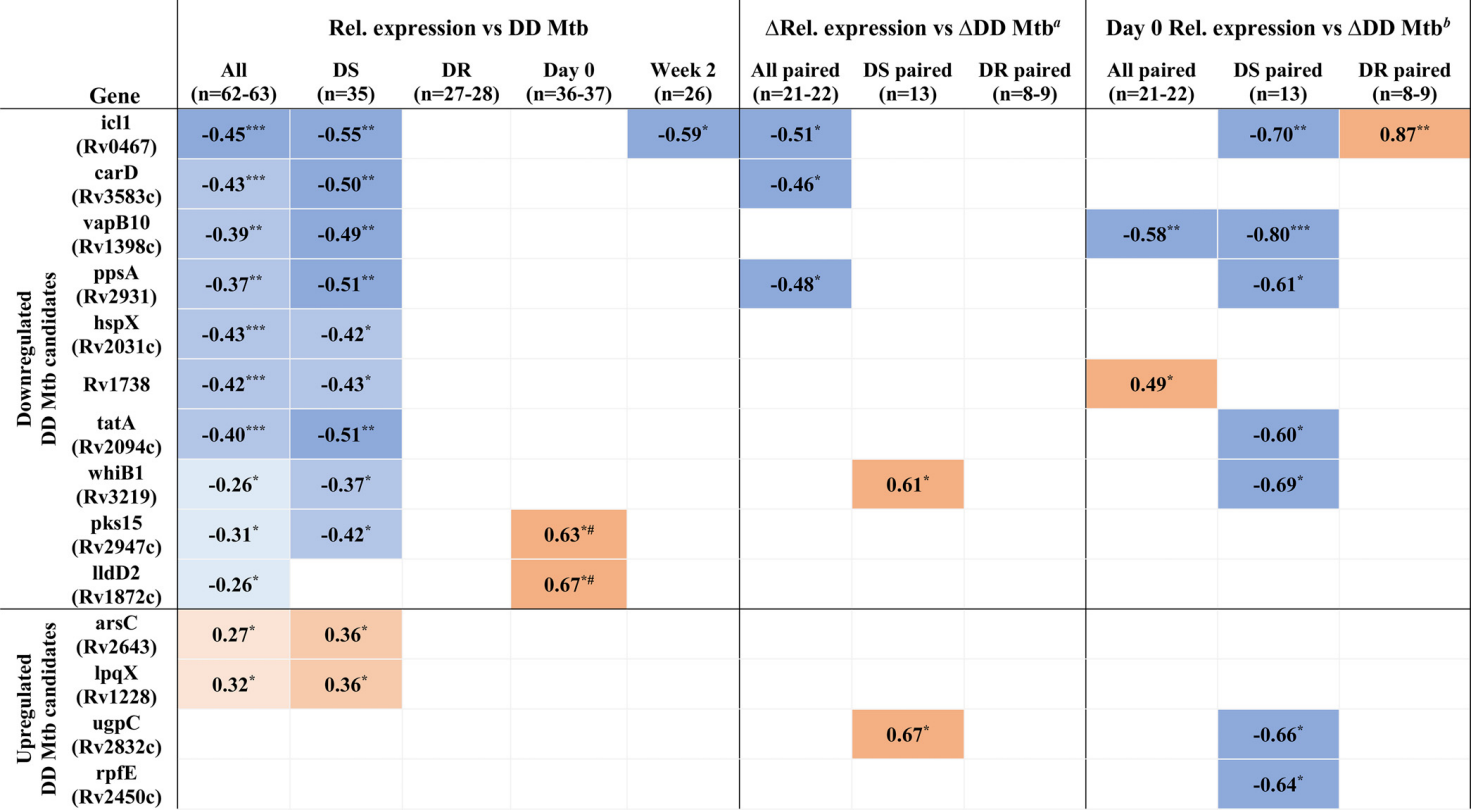
Summary of significant Spearman correlation coefficients for the relative expression of the 14 DD M. tuberculosis candidate genes with respect to the relative abundance of DD M. tuberculosis present in patient sputa for the different analyses/subanalyses performed in this study^*c*^

a ΔRel. expression versus ΔDD M. tuberculosis comparing week 2 versus day 0 values.

b ΔDD M. tuberculosis comparing week 2 versus day 0 values.

c Darker shade of blue corresponds to greater negative Spearman correlation coefficient; darker shade of orange corresponds to greater positive Spearman correlation coefficient. The highest Spearman correlation coefficient obtained from MPN^+CF^/CFU, MPN^−CF^/CFU, or MPN^Max^/CFU is presented. Rel, relative. Statistical significance shown as follows: *, *P* value < 0.05; **, *P* value < 0.01; ***, *P* value < 0.002; #, Spearman correlation coefficients reached statistical significance only for the DS cohort, which is presented here.

### Assessing whether changes in gene expression correlate with changes in DD M. tuberculosis.

**FIG 3:**
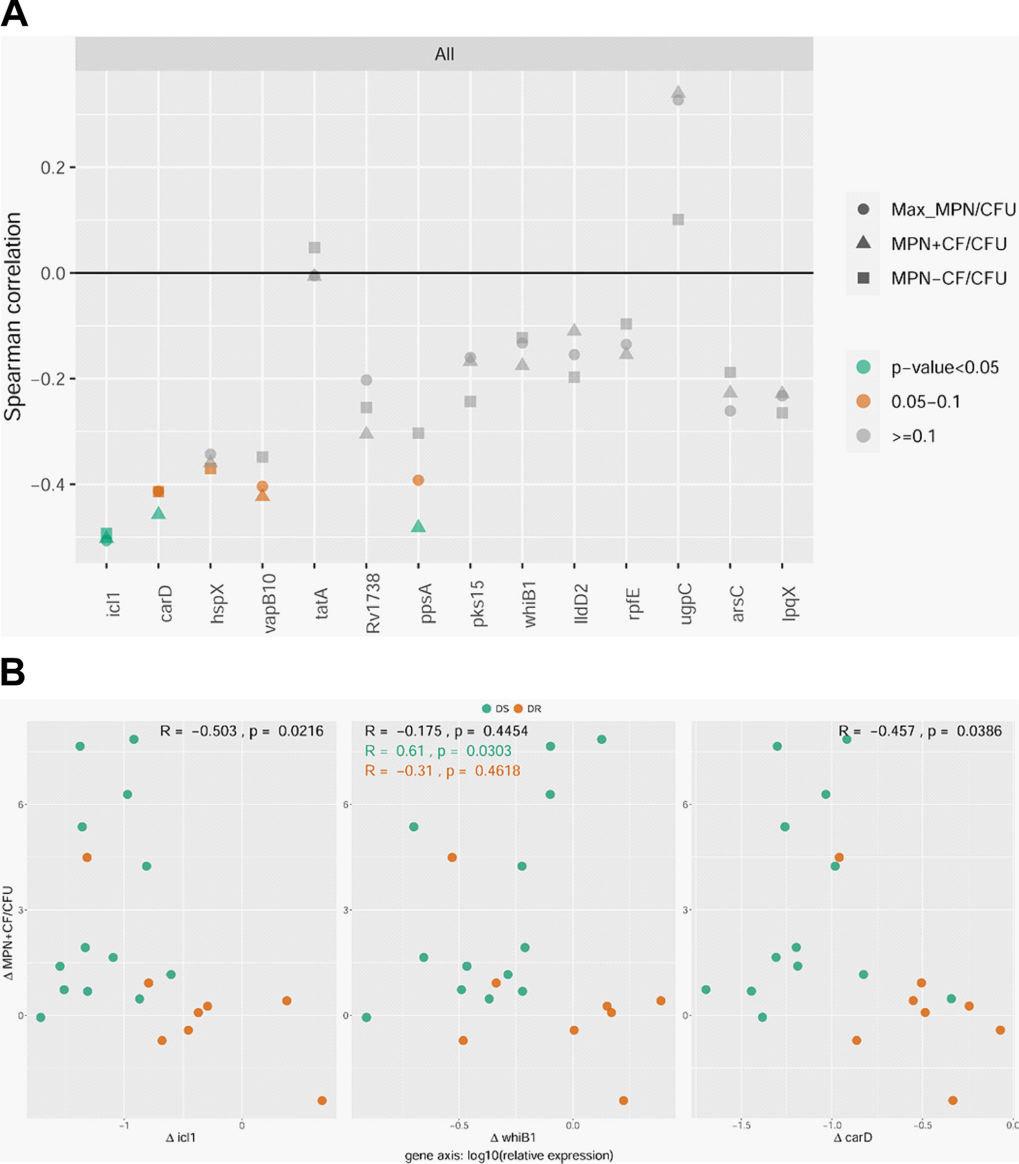
(A) Summary of Spearman correlation coefficients for the 14 DD M. tuberculosis candidate genes comparing Δ(relative gene expression) versus Δ(DD M. tuberculosis relative abundance) at week 2 versus day 0 for paired sputum samples from both cohorts. (B) Representative scatterplots for genes that show significant Spearman correlation coefficients as determined by the Δ(MPN^+CF^/CFU) ratio (comparing week 2 versus day 0). All, *n* = 21 to 22; DS, *n* = 13; DR, *n* = 8 to 9. Complete scatterplots and *R* and *P* values can be found in Fig. S4 and Table S4. MPN, most probable number; Max MPN, the maximum M. tuberculosis count obtained by MPN with or without CF; CF, culture filtrate.

Next, we focused on subjects from whom sputa from before (day 0) and after (week 2) initiation of therapy were both available. We asked if a change in expression after onset of therapy (in relation to before therapy) for any of the DD M. tuberculosis candidate genes correlated with a corresponding change in DD M. tuberculosis. When analyzing all paired samples from both cohorts (*n* = 21 to 22), a decrease in expression for three genes (*icl1*, *carD*, and *ppsA*) at week 2 showed a statistically significant correlation with an increase in DD M. tuberculosis at week 2 (and for a fourth gene, *vapB10*, it nearly reached significance, *P* value of 0.057) (Fig. 3, Fig. S4, and Table S4). When limiting analyses to paired samples from the DS cohort (*n* = 13), an increase in expression for two genes (*whiB1* and *ugpC*) at week 2 showed a statistically significant correlation with an increase in DD M. tuberculosis at week 2 (Fig. S4 and Table S4).

### Determining whether gene expression prior to treatment foretells generation of DD M. tuberculosis following treatment.

**FIG 4:**
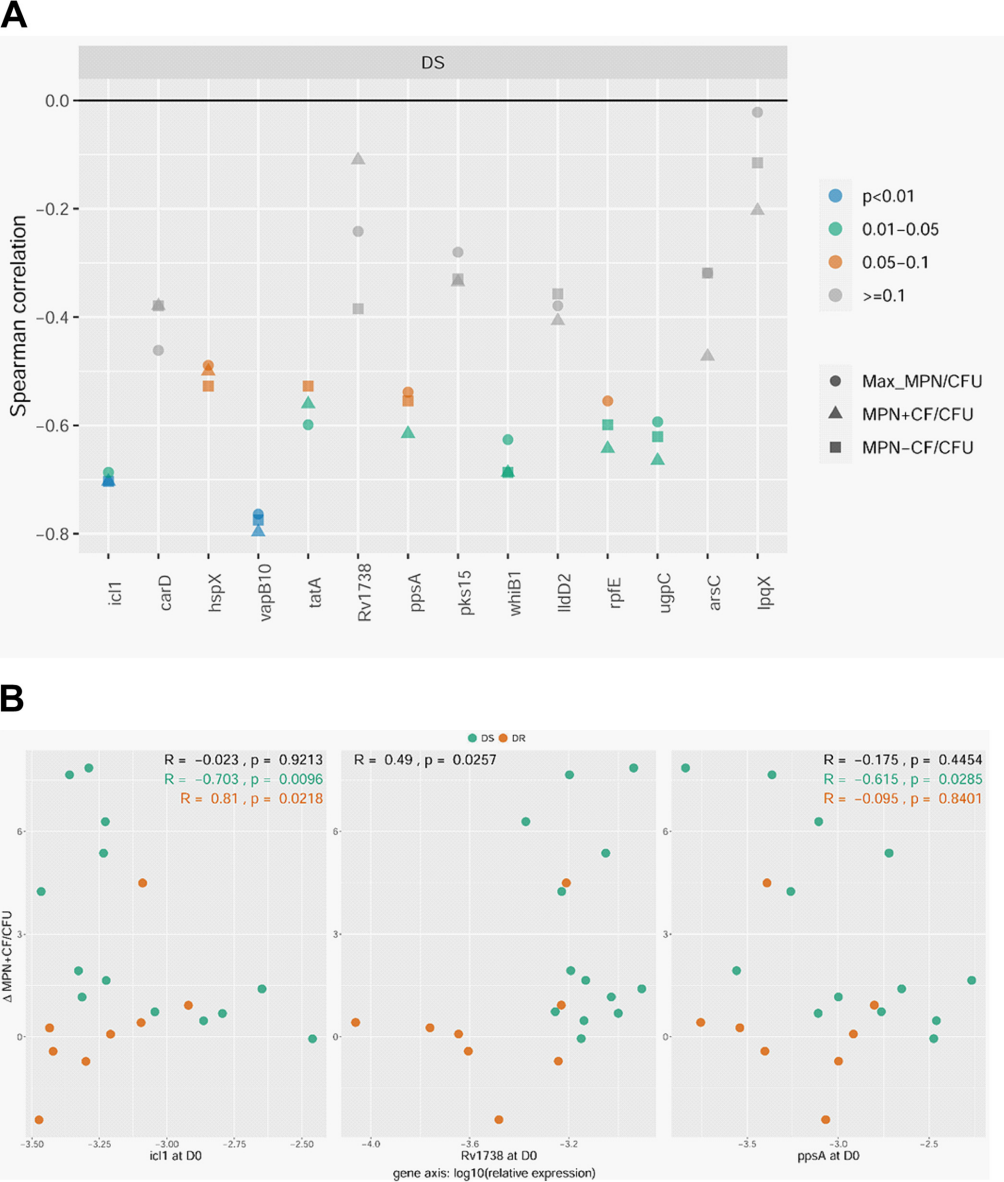
(A) Summary of Spearman correlation coefficients for the relative gene expression of the 14 DD M. tuberculosis candidate genes at day 0 with respect to Δ(DD M. tuberculosis relative abundance) at week 2 versus day 0 for paired sputum samples from the DS cohort. (B) Representative scatterplots for three genes that show significant Spearman correlation coefficients as determined by the Δ(MPN^+CF^/CFU) ratio. All, *n* = 21 to 22; DS, *n* = 13; DR, *n* = 8 to 9. Complete scatterplots and *R* and *P* values can be found in Fig. S5 and Table S5. D0, day 0; MPN, most probable number; Max MPN, the maximum M. tuberculosis count obtained by MPN with or without CF; CF, culture filtrate.

We were next curious about whether the expression of any of the DD M. tuberculosis candidates specifically at day 0 correlated with a positive change in DD M. tuberculosis at week 2. Put another way, did the expression of any of the genes prior to treatment foretell generation of DD M. tuberculosis after initiation of treatment? When analyzing all paired samples from both cohorts (*n* = 21 to 22), expression of *rv1738* (a bacterial hibernation factor) at day 0 showed a positive association, and expression of *vapB10* (an antitoxin) at day 0 showed a negative association with an increase in DD M. tuberculosis at week 2 (Fig. 4 and Table 3). When limiting analyses to paired samples from the DS cohort (*n* = 13), the expression of seven genes at day 0 (including *icl1*, *ppsA*, *whiB1*, *ugpC*, and *vapB10*) showed a negative correlation with an increase in DD M. tuberculosis at week 2 (Fig. 4, Fig. S5, and Table S5). In the DR cohort (*n* = 8 to 9), *icl1* was the only gene whose expression at day 0 showed an association (positive) with an increase in DD M. tuberculosis after initiation of therapy (Fig. S5 and Table S5).

10.1128/mbio.02701-22.5 FIG S5 Spearman correlations for the relative gene expression of the 14 DD M. tuberculosis candidate genes at day 0 with respect to Δ(DD M. tuberculosis relative abundance) at week 2 versus day 0 for paired sputum samples from patients with drug-sensitive (DS) or drug-resistant (DR) TB before and after initiation of first-line or second-line therapy, respectively. All, *n* = 21 to 22; DS, *n* = 13; DR, *n* = 8 to 9. Complete scatterplots and *R* and *P* values can be found below and in Table S5. MPN, most probable number; Max MPN, the maximum M. tuberculosis count obtained by MPN with or without CF; CF, culture filtrate; D0, day 0. Download FIG S5, PDF file, 0.8 MB. Copyright © 2022 Zainabadi et al. 2022 Zainabadi et al. https://creativecommons.org/licenses/by/4.0/ This content is distributed under the terms of the Creative Commons Attribution 4.0 International license.

10.1128/mbio.02701-22.10 TABLE S5 Summary of Spearman correlation coefficients comparing the relative gene expression of the 14 DD M. tuberculosis candidate genes at day 0 with respect to Δ(DD M. tuberculosis relative abundance) as determined by the Δ(MPN/CFU) ratio at week 2 versus day 0 for all paired sputum samples or paired sputa from the individual cohorts (DS and DR). *P* values of <0.05 are highlighted in red; *P* values of 0.05 to 0.10 are highlighted in blue. MPN, most probable number; MPN^Max^, the maximum M. tuberculosis number obtained by MPN with or without CF; CF, culture filtrate; DS, drug sensitive; DR, drug resistant; D0, day 0. Download Table S5, DOCX file, 0.02 MB. Copyright © 2022 Zainabadi et al. 2022 Zainabadi et al. https://creativecommons.org/licenses/by/4.0/ This content is distributed under the terms of the Creative Commons Attribution 4.0 International license.

## DISCUSSION

The goal of this study was to answer the question whether DD M. tuberculosis generated *in vitro* by nutrient deprivation followed by rifampin exposure resembles DD M. tuberculosis that occurs in patients. Using gene expression profiles as a proxy, this pilot study provides evidence for overlap between these two cell populations: 12 of 14 genes found differentially expressed in the PBS-RIF model showed a similar expression profile in patient sputa that correlated with the relative abundance of DD M. tuberculosis. It is notable that these correlations were found when including sputa from subjects with DS and DR TB both before and after initiation of therapy and that seven of the 12 DD M. tuberculosis candidate genes also showed correlations with DD M. tuberculosis in at least one additional analysis, including with changes in the relative abundance of DD M. tuberculosis after initiation of therapy (Table 3). Culture filtrate (CF)—which has been shown to promote recovery of certain DD M. tuberculosis populations—improved the Spearman correlation coefficients for most genes, supporting a DD M. tuberculosis-specific effect. Collectively, these data present preliminary evidence that the DD M. tuberculosis generated *in vitro* by Saito et al. (11) resembles DD M. tuberculosis from patients with respect to aspects of their transcriptional profile.

Other models of DD M. tuberculosis generation involve other forms of stress, such as heat stress (20), desiccation (20), and potassium deprivation (22, 24) (the PBS used in the PBS-RIF model contains potassium [11]). These models may represent distinct DD M. tuberculosis populations, both *in vitro* and in patients, and thus may have transcriptional profiles that do not necessarily overlap. To increase our chances of selecting for DD M. tuberculosis candidates that have broader roles in DD M. tuberculosis biology, we favored genes that had been found differentially expressed in a second model of DD M. tuberculosis generation (which uses potassium starvation and a 20-fold-lower dose of rifampin) (22). Transcriptional data for the remaining DD M. tuberculosis models were not available. Additionally, we also favored genes that had been implicated in models of M. tuberculosis persistence (23). These selection criteria selected for genes nearly all of which showed some level of association with DD M. tuberculosis in patient sputa.

One of the DD M. tuberculosis candidate genes, *isocitrate lyase* (*icl1*), was recently shown to play a restraining role in DD M. tuberculosis formation: M. tuberculosis cells lacking *icl1* showed enhanced DD M. tuberculosis generation in the PBS-RIF model (20). It is therefore notable that in our analyses, the expression of *icl1* showed a negative correlation with the proportion of DD M. tuberculosis cells present in patient sputa in five separate analyses, the most of any DD M. tuberculosis candidate gene. In the PBS-RIF model, *icl1* was one of the most downregulated genes (20), and in the present study we found *icl1* expression to display the strongest negative correlation with DD M. tuberculosis in patient sputa. In fact, low *icl1* expression was the single best predictor of whether a sputum sample was positive for DD M. tuberculosis, with an AUC of 0.78. Combining *icl1* expression with that of two additional DD M. tuberculosis candidate genes, *lpqX* and *rv1738*, resulted in an AUC of 0.88. Therefore, DD M. tuberculosis gene expression profiles may prove useful for monitoring the presence of DD M. tuberculosis in sputa. The utility of such an assay will require validation in a future trial examining an independent cohort of patients.

The gene that most closely clusters with *icl1* in hierarchal analyses is *carD*, an RNA polymerase-binding transcription factor involved in stress response (25) (Fig. S2). This may be explained by the fact that CarD regulates the expression of *icl1* in Mycobacterium smegmatis and M. tuberculosis (26, 27). The expression of four other DD M. tuberculosis candidate genes has also been found to be under possible regulation by CarD: *hspX*, *rv1738*, *arsC*, and *rpfE* (27). These observations, in addition to CarD’s established role in regulating M. tuberculosis sensitivity to rifampin, response to oxidative stress, and persistence in animal models, make it a top candidate for future research (26).

Our incomplete understanding of DD M. tuberculosis biology makes it difficult to ascribe biological significance for the observed gene expression changes. We imagine two scenarios in which a gene may associate with DD M. tuberculosis. First, a gene may associate directly with the DD M. tuberculosis phenotype, in which case the gene’s expression would correlate both with the relative abundance of DD M. tuberculosis and with changes in the relative abundance of DD M. tuberculosis in sputa over time. At least three genes meet these criteria: *icl1*, *carD*, and *ppsA* (Table 3). Second, a gene may be involved in promoting DD M. tuberculosis formation (in non-DD M. tuberculosis cells) in response to drug pressure and/or other stressors. For this class of genes, their expression specifically at day 0 (prior to drug pressure) would be expected to correlate with an increase in DD M. tuberculosis following drug pressure. At least two genes meet this criterion: *rv1738* (a bacterial hibernation factor) and *vapB10* (an antitoxin). Rv1738 promotes nonreplicative persistence (28), and *rv1738* was the only gene whose expression at day 0 positively correlated with an increase in DD M. tuberculosis at week 2. Reduced expression of the antitoxin *vapB10* may serve a similar function by allowing the activity of its cognate toxin protein, VapC10, which in other bacterial systems causes growth arrest (29). By halting replication, both genes may prime M. tuberculosis cells to enter the DD state upon drug pressure, perhaps akin to PBS starvation in the PBS-RIF model.

A limitation of the current study is the relatively small cohort size used for analyses, which makes data interpretation particularly difficult in subanalyses. For example, when analyzing the cohorts separately, it may be tempting to conclude that the correlations between gene expression and DD M. tuberculosis are specific to the DS cohort. However, the lack of statistical significance in the DR cohort may simply reflect its smaller sample size and the lower relative abundance of DD M. tuberculosis. Consistent with this, while statistical significance was not reached in the DR cohort, trends in the same direction were observed for 10 of the 12 DD M. tuberculosis candidate genes (see Table S3 in the supplemental material). For certain genes, opposite correlations between gene expression and relative abundance of DD M. tuberculosis were found depending on the analysis or subanalysis. For example, *icl1* expression in pretreatment sputa showed a negative or positive correlation with an increase in DD M. tuberculosis following treatment depending on which cohort was analyzed (Table 3). This discrepancy may reflect the differing drug regimens taken by the two cohorts (15), among other variables. Future trials examining larger cohort sizes and incorporating control genes whose expression does not correlate with DD M. tuberculosis in the PBS-RIF model should help in answering these questions.

Our results indicate that the PBS-RIF model has relevance to DD M. tuberculosis recovered from patients. This supports using the *in vitro* model to uncover physiologically relevant aspects of DD M. tuberculosis biology that can be evaluated in the clinic, while observations made in the clinic can generate hypotheses that can be experimentally tested with the *in vitro* model. The transcriptional profiles identified here may also have utility for tracking DD M. tuberculosis populations, which may be particularly useful for identifying drugs that can kill these cells in patients. This may ultimately help in determining whether the presence of DD M. tuberculosis (or, rather, the presence of a DD M. tuberculosis-like gene expression profile) in sputum correlates with slower or otherwise worse treatment outcomes in TB patients.

## MATERIALS AND METHODS

### Study design and patient populations.

This prospective observational study was performed at the Groupe Haïtien d’Étude du Sarcome de Kaposi et des Infectieuses Opportuniste (GHESKIO) centers in Port au Prince, Haiti, and was approved by the institutional review boards of both GHESKIO and Weill Cornell Medicine. All participants provided written informed consent and scored at least 90% on a quiz for assessment of understanding before enrollment. All aspects about the study design, including exclusion/inclusion criteria and patient characteristics, have been published previously (15).

Participants with drug-sensitive (DS) or drug-resistant (DR) TB had a positive Xpert M. tuberculosis/RIF assay (Cepheid, Sunnyvale, CA) without or with indication of rifampin resistance, respectively. Subjects with DS TB were followed in the GHESKIO outpatient clinic for the duration of their directly observed therapy (DOT). These participants were all treatment naive at time of enrollment and received isoniazid (H), rifampin (R), ethambutol (E), and pyrazinamide (Z) for 2 months and then HR for 4 months. Participants with DR TB were not excluded if they had been treated for drug-sensitive TB. These participants were hospitalized in GHESKIO’s inpatient multidrug-resistant TB hospital for approximately the first 4 months of treatment with DOT regimens comprised of bedaquiline, levofloxacin, linezolid, clofazimine, and pyrazinamide. Bedaquiline was discontinued after 6 months and linezolid after 12 months, with the remaining drugs continued to complete 20 months of therapy.

### Sputum processing and microbiological assays.

Overnight sputum samples (5 p.m. to 9 a.m.) were self-collected by participants and stored in a cool box with ice packs (4°C) until delivery to GHESKIO. Decontamination of sputum, preparation of culture filtrate (CF), and protocols for CFU and MPN-LD assays have been reported previously (11, 14, 15). The proportion of DD M. tuberculosis present in patient sputa was represented as the ratio of the viable M. tuberculosis count per milliliter obtained from the MPN-LD assays to that from CFU assays. MPN-LD assays performed without CF are referred to as MPN^−CF^, those with CF as MPN^+CF^, and the highest M. tuberculosis number per milliliter obtained between the two as MPN^Max^. This was necessary as CF at times promotes and at times impedes M. tuberculosis growth from patient sputa (12–16). All microbiological work took place in a biosafety level 3 laboratory with appropriate safety guidelines and personal protective equipment.

### RNA and qRT-PCR experiments.

The RNA-seq data set used to identify DD M. tuberculosis candidate genes has been published previously (20). For RNA extraction from TB patient sputum, a novel protocol was devised for this study that improved recovery of M. tuberculosis RNA but prevented copurification of M. tuberculosis DNA from patient sputum (30). RNA was extracted from 1 mL of neat sputum containing at least 1,000 M. tuberculosis bacteria per mL.

Quantitative reverse transcription-PCR (qRT-PCR) for M. tuberculosis 16S rRNA and two DD M. tuberculosis mRNA candidates was run in a multiplexed reaction (incorporating a maximum of three primer sets and three probes) in triplicate using QuantiTect multiplex RT-PCR master mix (Qiagen catalog no. 204645) and a Roche LC96 instrument (30). The use of the Qiagen QuantiTect multiplex kit is advised as we have found it ideally suited for multiplexed reactions and highly resistant to qRT-PCR inhibitors (30–33).

The expression of DD M. tuberculosis candidate genes was normalized to M. tuberculosis 16S rRNA (*sigA* expression in pretreatment sputa was found to be too low for use in normalization). Sequences for 16S rRNA primers and probe were obtained from the work of Choi et al. (see Table S1 in the supplemental material) (34), sequences from which have been shown to be specific to M. tuberculosis. We confirmed this by digital PCR (which indicated the probe was highly specific for M. tuberculosis) and by testing on 10 non-TB sputum samples. The 16S rRNA primer set shows a strong correlation with M. tuberculosis numbers as determined by culture (30) and fails to amplify 16S rRNA in H37Rv sterilized by treatment with rifampin and isoniazid for 4 weeks (as determined by CFU and mycobacterial growth indicator tube [MGIT]) (30).

We used high-throughput qRT-PCR expression data from the work of Walter et al. (21) to rate the expression of each M. tuberculosis mRNA candidate in patient sputum. For each gene, at least four different primers (and one probe) were designed in order to yield a total of four possible amplicons. These were tested on RNA extracted from pooled pretreatment TB patient sputum samples to identify the primer set that yielded the lowest overall qRT-PCR cycle threshold (*C_T_*) value. *C_T_* values for each gene from this experiment were then ranked and compared to those from the work of Walter et al. (21). Only the most highly expressed genes (i.e., genes with the lowest *C_T_* value) were selected for further study.

Downregulated and upregulated DD M. tuberculosis candidate genes were not run together in the same multiplexed qRT-PCR in order to prevent any bias in which increased expression of one gene may artificially suppress the other due to competition for PCR reagents. As a negative control, the same reaction was run without the reverse transcriptase (RT) enzyme to confirm lack of DNA amplification. Two microliters of RNA was used in a 10-μL final quantitative PCR (qPCR) volume (30). All primers and probes were used at 0.1 μM final concentrations (Table S1).

### Statistical analyses.

Following exploratory data analysis using descriptive statistics and visualizations, we present association analysis taking pairs of continuous variables: relative gene expression and the proportion of DD M. tuberculosis (represented by MPN/CFU). First, the relationship between relative gene expression and proportion of DD M. tuberculosis was assessed for all available sputum samples regardless of timing of sample collection (pre- or posttreatment) or cohort (drug sensitive or drug resistant). Second, noting some sputum samples share patient-level characteristics since they were collected from the same patients at different time points, we aimed to reduce interpatient variability in the association study by making comparisons within the same subjects. Using only pre- and posttreatment matched paired samples, we assessed the association of within-subject changes in relative gene expression versus within-subject changes in the proportion of DD M. tuberculosis. We defined within-subject changes in relative gene expression and in the proportion of DD M. tuberculosis by subtracting the pretreatment quantities from the posttreatment quantities. Lastly, we aimed to assess the association from a predictive model-building perspective and assessed monotonic association of within-patient changes in the proportion of DD M. tuberculosis and relative gene expression specifically at day 0.

In all three association analyses, Spearman rank-based correlation coefficients were used incorporating a metric between −1 and 1 to measure the strength and direction of the monotonic relationship between two variables, rather than linear relationship. We chose this metric over the Pearson correlation coefficient because scatterplots illustrated that the associations were not necessarily best described by lines, and monotonic association is sufficient for the purpose of this study. For all sample association studies, *P* values were adjusted using the Benjamini-Hochberg method to account for the simultaneous testing of hypotheses for multiple genes. For association studies with pre- and posttreatment paired samples, by comparing samples from the same patients, sample size was cut by more than half while interpatient variability was reduced, and thus, *P* values were not adjusted for multiple comparisons. In order to identify groups of genes exhibiting similar univariate associations with DD M. tuberculosis, we used Euclidian distance between Spearman correlation coefficients for a gene pairwise dissimilarity metric and performed agglomerative hierarchical clustering using complete linkage.

We defined DD M. tuberculosis as being present in a sputum sample when the MPN value was more than the upper bound of the 95% confidence interval of the CFU value. We then evaluated the utility of gene expression profiles as a diagnostic test for the presence or absence of DD M. tuberculosis in sputa by evaluating sensitivity and specificity with a receiver operating characteristic (ROC) curve based on area under the curve (AUC). First, the presence of DD M. tuberculosis is predicted based on relative expression of each gene individually (i.e., lower gene expression than a cutoff for downregulated DD M. tuberculosis candidate genes and higher gene expression than a cutoff for upregulated DD M. tuberculosis candidate genes). For a given cutoff value, sensitivity and specificity are evaluated and a ROC curve plots the sensitivity against the 1-specificity for all possible cutoff values (i.e., low to high). Given the small sample size, to assess performance of the diagnostic test, we decided not to choose a single optimal cutoff to evaluate sensitivity and specificity values. Instead, we focused on evaluating the potential utility of gene expression as a diagnostic test by investigating a spectrum of cutoff values to obtain corresponding pairs of sensitivity and specificity values. Second, we considered a multivariate DD M. tuberculosis prediction model which combined information based on expression of multiple genes and presented ROC curve plots. For a single gene expression prediction model for the presence of DD M. tuberculosis, we estimated the prediction performance by repeated 10-fold cross-validation (35). With the current sample size, however, we determined that multiple gene expression models are far too complex to cross-validate the models’ predictive power, and thus, k-fold cross-validation estimates are not reported for multivariate DD M. tuberculosis models. Analyses were performed using R version 4.0.1 and tidyverse, ggpubr, and pROC packages.
