# Supplementary figures and images for: Transcriptional Biomarkers of Differentially Detectable Mycobacterium tuberculosis in Patient Sputum
Source: mBio. 2022 Nov 3;13(6):e02701-22. doi: 10.1128/mbio.02701-22 (PMC9765512; doi:10.1128/mbio.02701-22)

# A

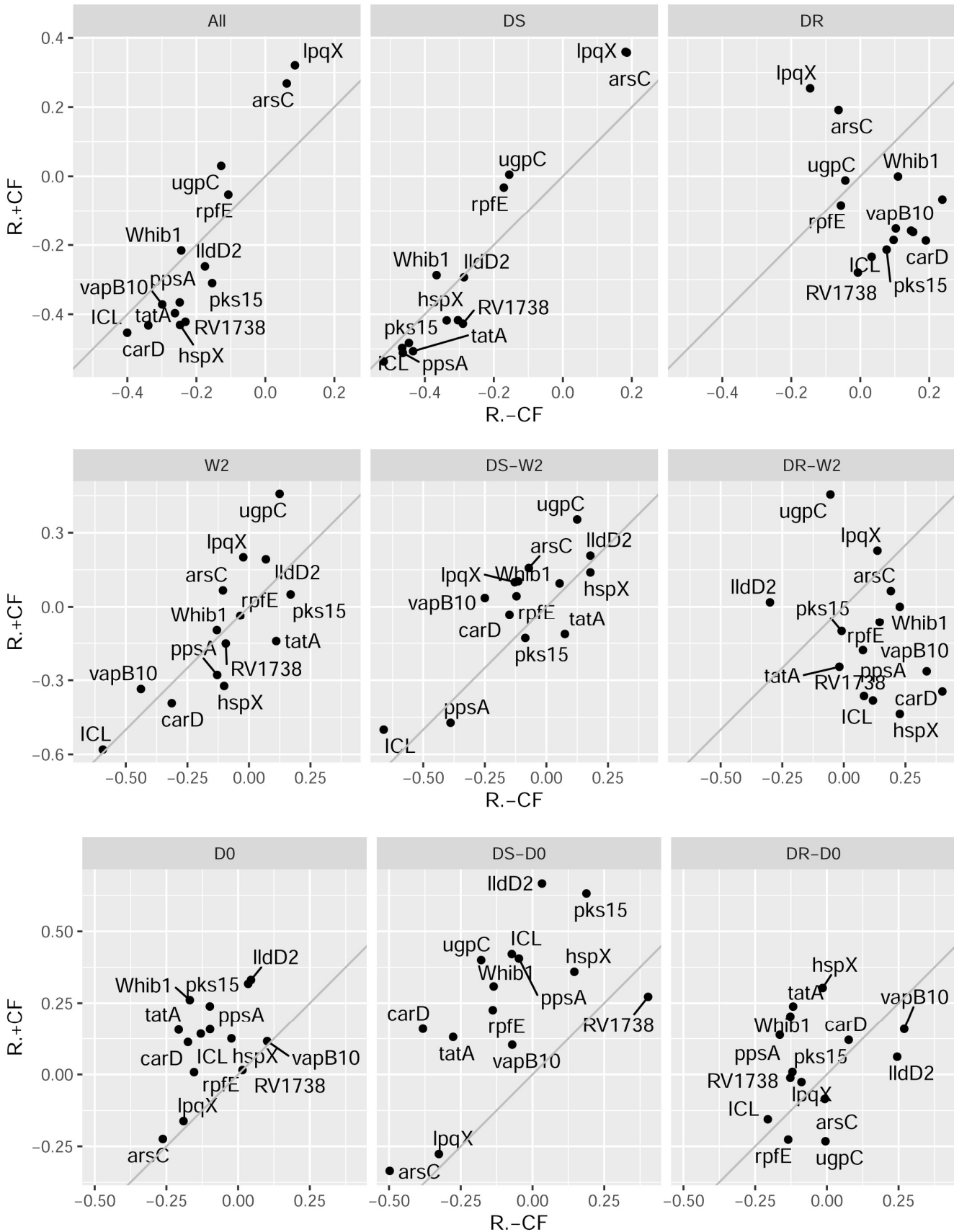

**B**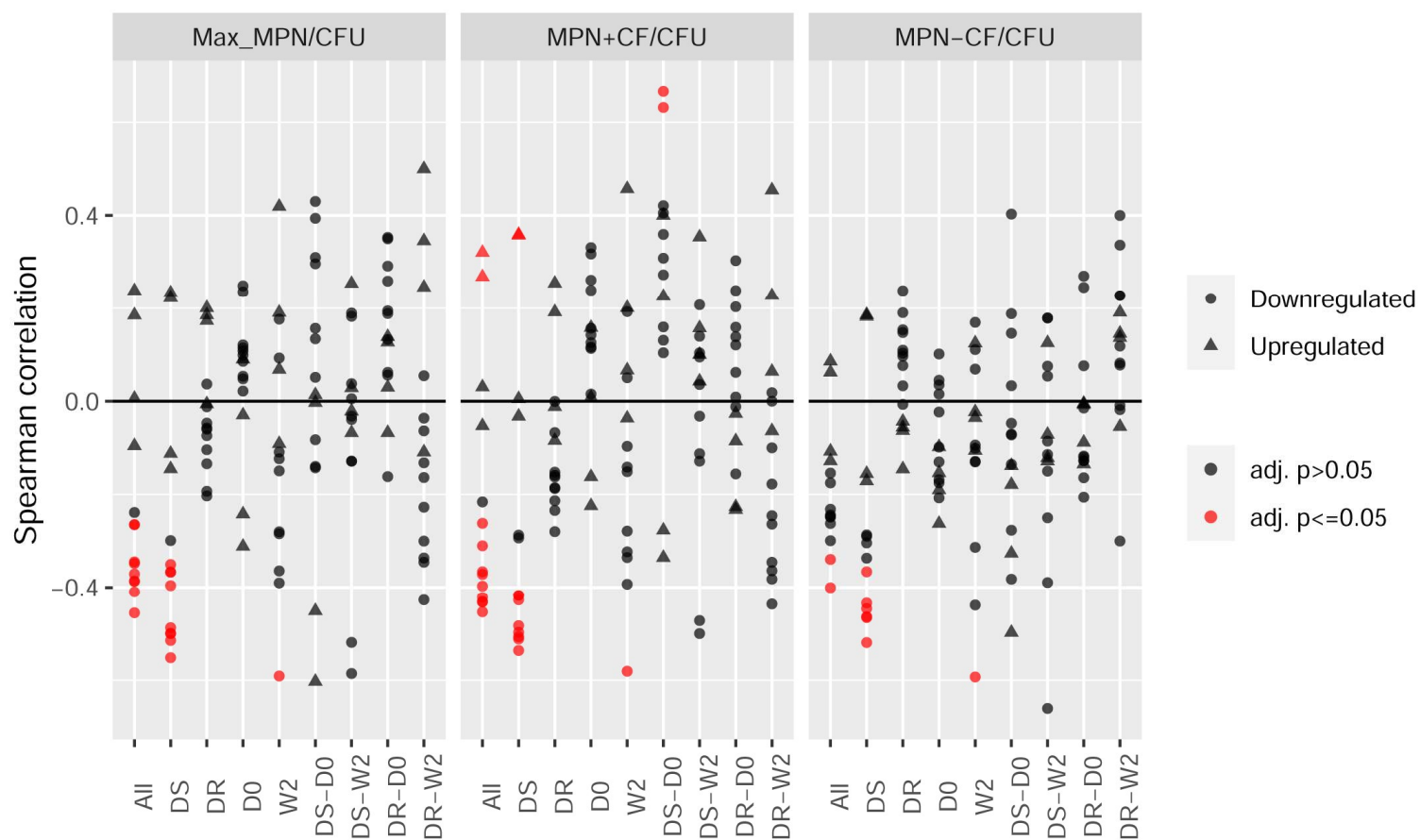

Supplement: FIG S1 [file mbio.02701-22-s0001.pdf]

Cluster Dendrogram

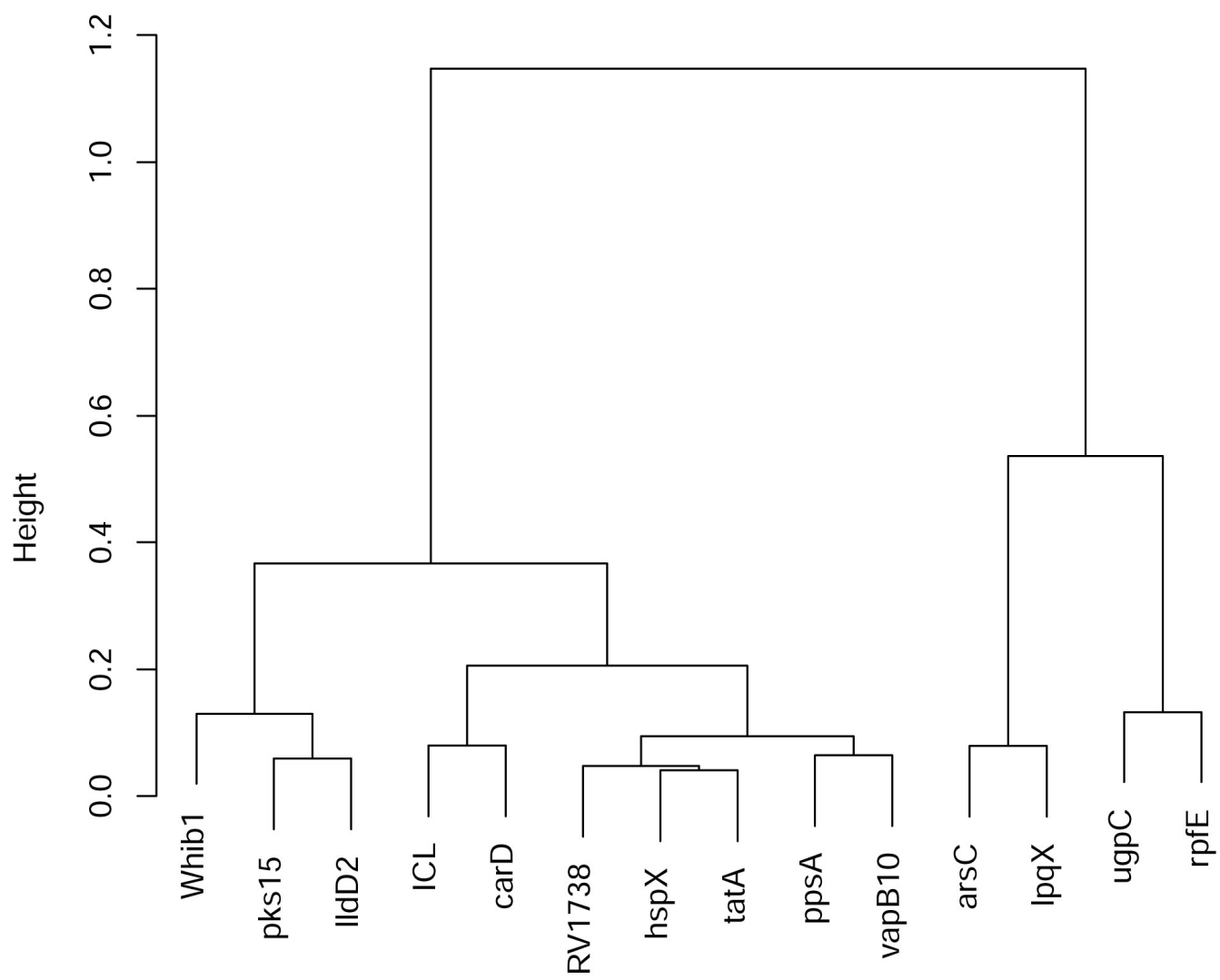

Supplement: FIG S2 [file mbio.02701-22-s0002.pdf]

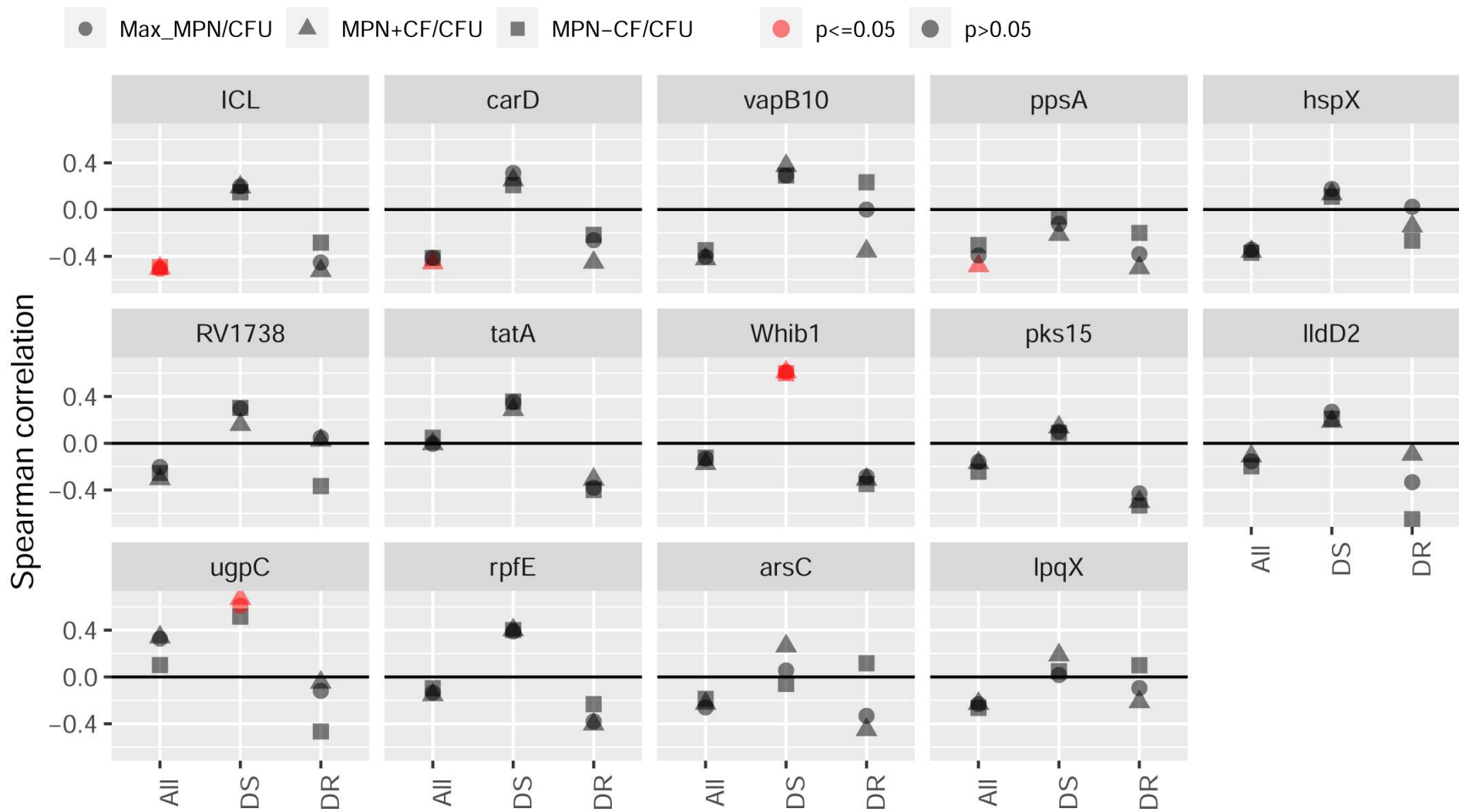

# All Paired Samples

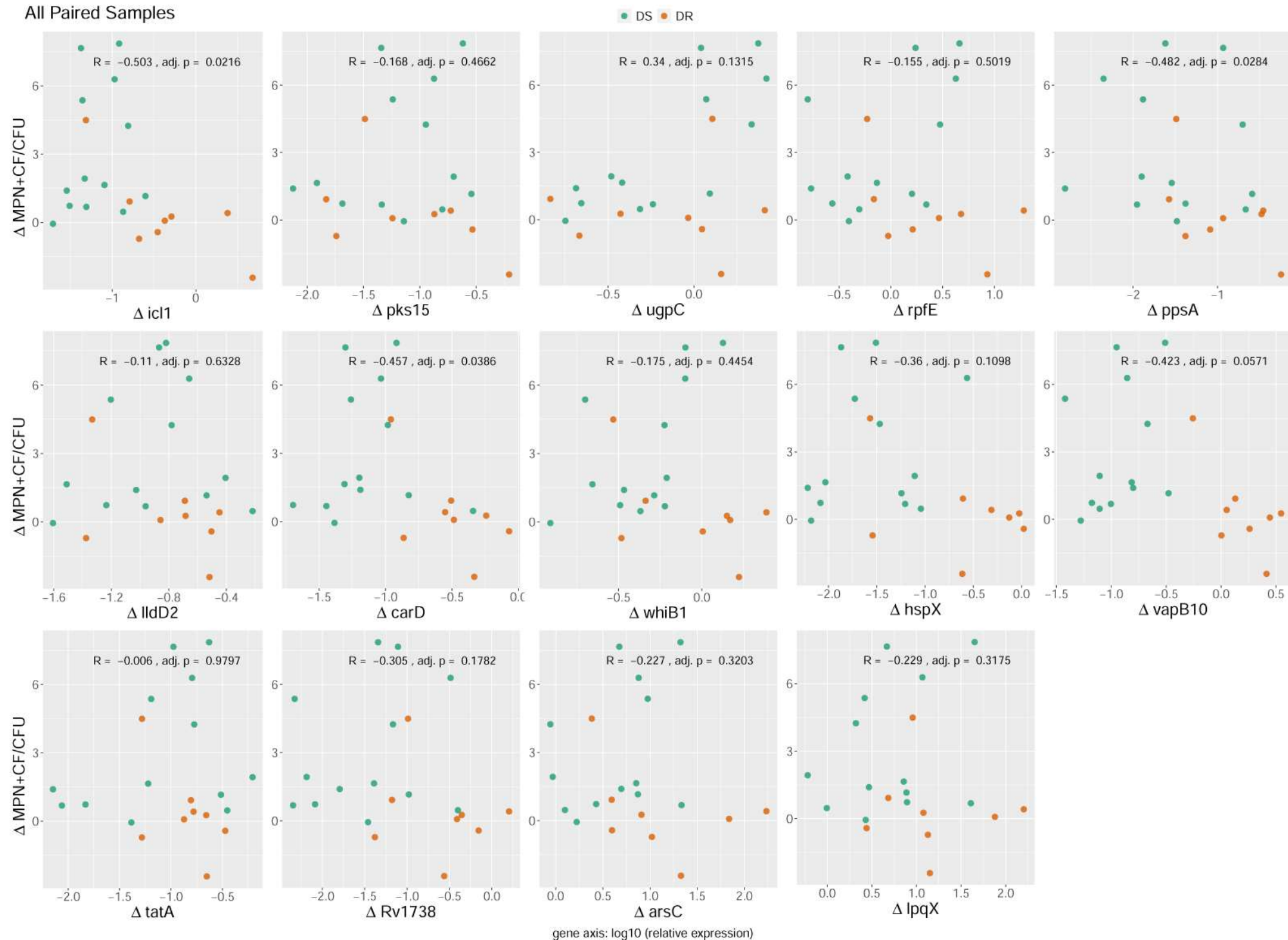

# All Paired Samples

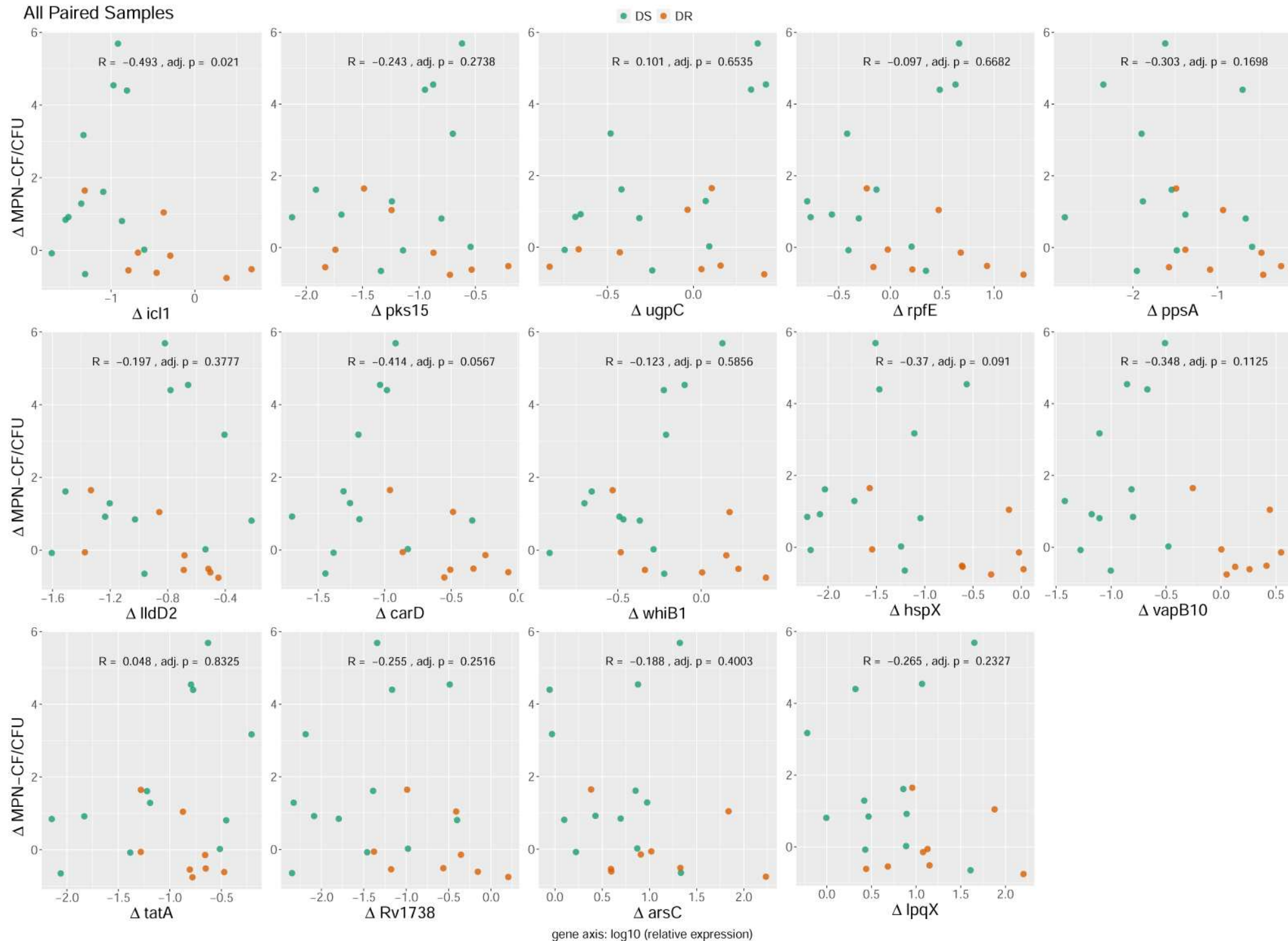

# All Paired Samples

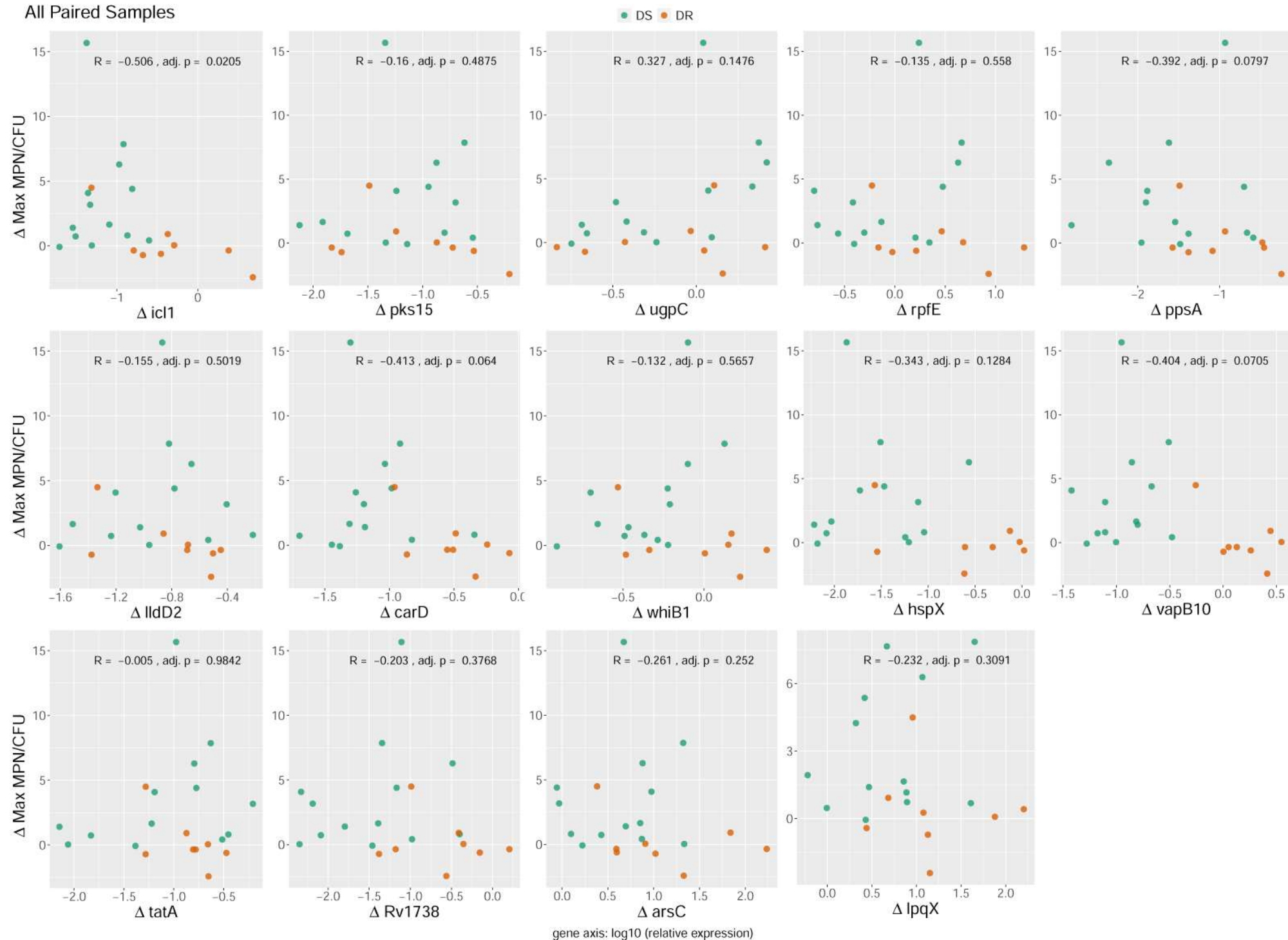

Supplement: FIG S4 [file mbio.02701-22-s0004.pdf]

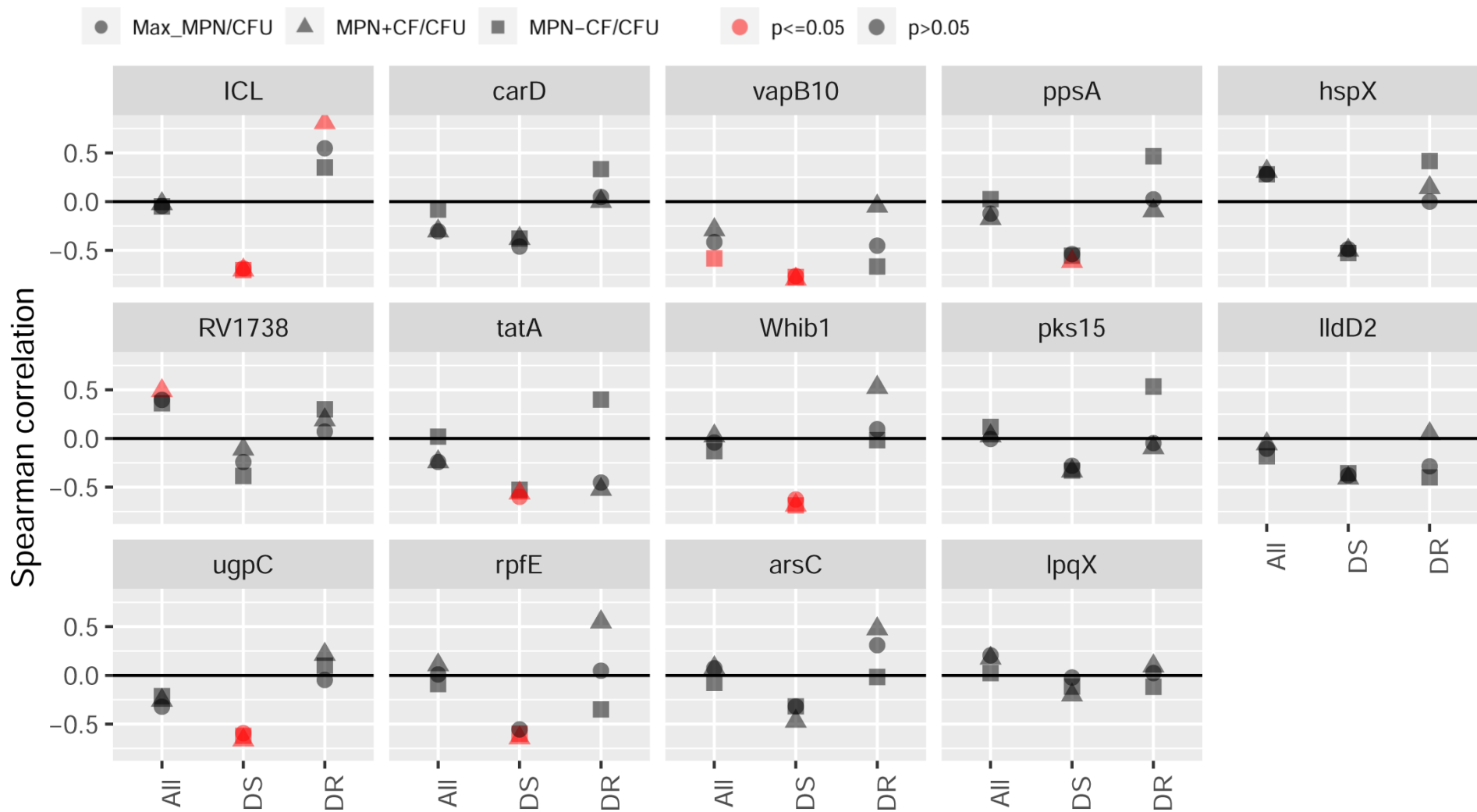

# All Paired Samples

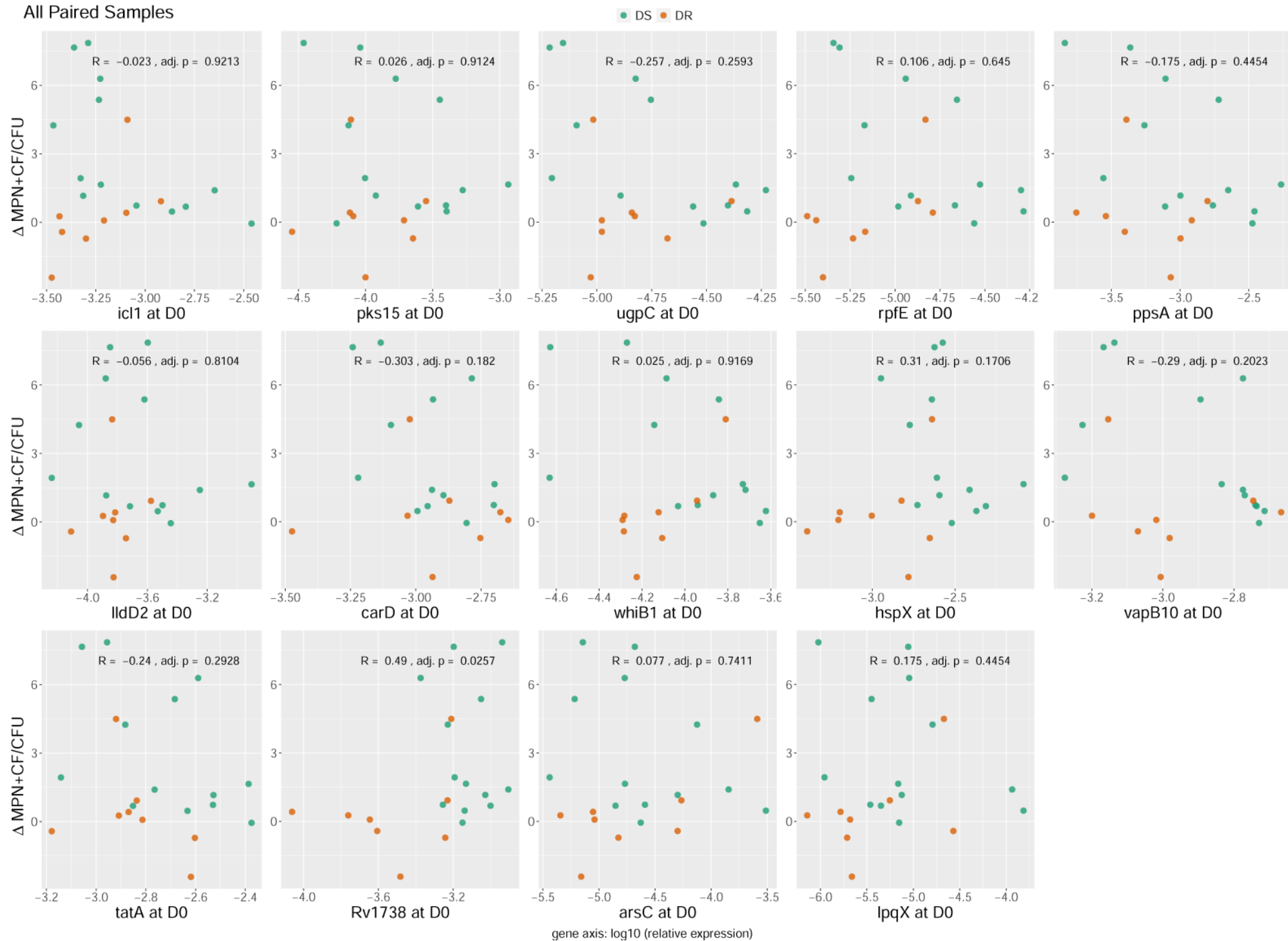

# All Paired Samples

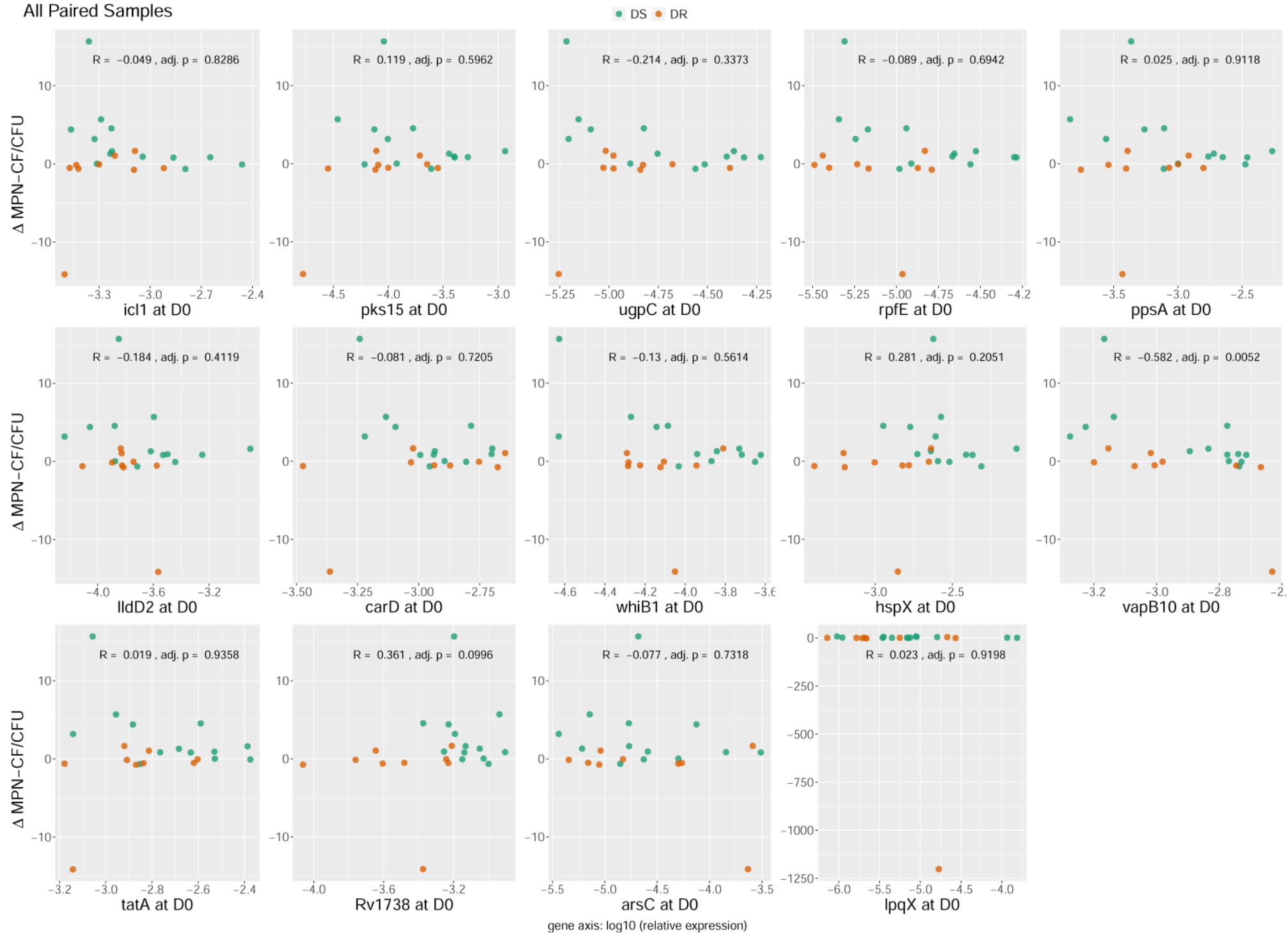

# All Paired Samples

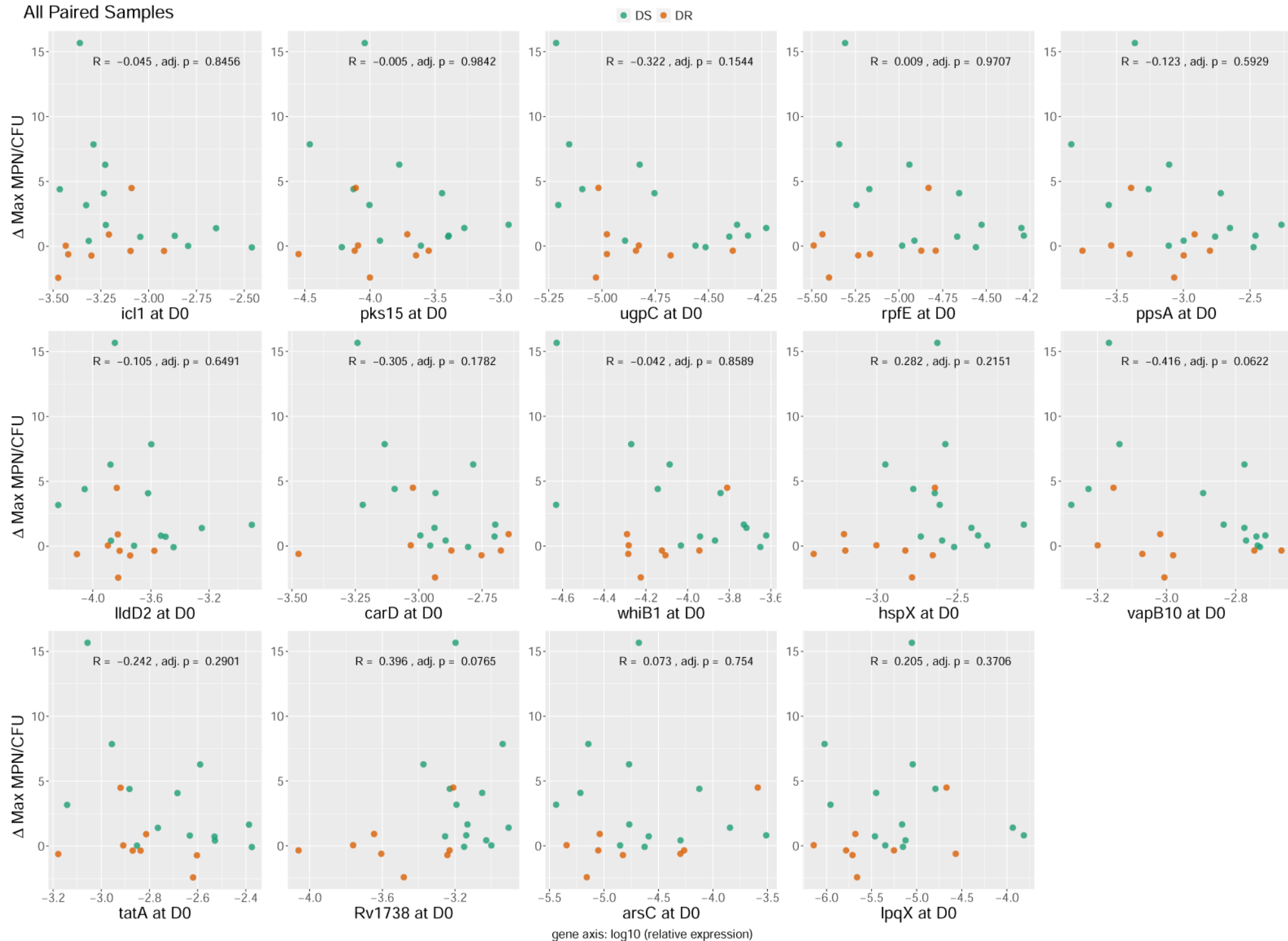

Supplement: FIG S5 [file mbio.02701-22-s0005.pdf]
