# Supplementary material for: Transcriptional Biomarkers of Differentially Detectable Mycobacterium tuberculosis in Patient Sputum
Source: mBio. 2022 Nov 3;13(6):e02701-22. doi: 10.1128/mbio.02701-22 (PMC9765512; doi:10.1128/mbio.02701-22)
Supplement: TABLE S3 [file mbio.02701-22-s0008.docx]

**ALL SAMPLES**

|  |  |  | **MPN^Max^/CFU**  **(n=62)** | | **MPN^+CF^/CFU**  **(n=62)** | | **MPN^-CF^/CFU**  **(n=63)** | |
| --- | --- | --- | --- | --- | --- | --- | --- | --- |
|  | **Gene** | **Rv#** | **Spearman correlation** | **Adj p-val** | **Spearman correlation** | **Adj p-val** | **Spearman correlation** | **Adj p-val** |
| **Downregulated**  **DD Mtb candidates** | **icl1** | **Rv0467** | -0.455 | 0.0013 | -0.453 | 0.001 | -0.401 | 0.0083 |
|  | **carD** | **Rv3583c** | -0.410 | 0.0033 | -0.432 | 0.001 | -0.339 | 0.0276 |
|  | **vapB10** | **Rv1398c** | -0.386 | 0.0037 | -0.372 | 0.0037 | -0.299 | 0.0538 |
|  | **ppsA** | **Rv2931** | -0.348 | 0.007 | -0.366 | 0.0037 | -0.248 | 0.0813 |
|  | **hspX** | **Rv2031c** | -0.387 | 0.0037 | -0.431 | 0.001 | -0.247 | 0.0813 |
|  | **Rv1738** | **Rv1738** | -0.345 | 0.007 | -0.422 | 0.001 | -0.232 | 0.0907 |
|  | **tatA** | **Rv2094c** | -0.371 | 0.0044 | -0.397 | 0.0019 | -0.262 | 0.0813 |
|  | **whiB1** | **Rv3219** | -0.265 | 0.0421 | -0.216 | 0.0848 | -0.244 | 0.0813 |
|  | **pks15** | **Rv2947c** | -0.265 | 0.0421 | -0.310 | 0.0143 | -0.154 | 0.2779 |
|  | **lldD2** | **Rv1872c** | -0.239 | 0.0601 | -0.262 | 0.0367 | -0.175 | 0.2237 |
| **Upregulated DD Mtb candidates** | **arsC** | **Rv2643** | 0.185 | 0.1465 | 0.268 | 0.0348 | 0.062 | 0.6077 |
|  | **lpqX** | **Rv1228** | 0.238 | 0.0601 | 0.321 | 0.0119 | 0.086 | 0.5136 |
|  | **ugpC** | **Rv2832c** | 0.006 | 0.9626 | 0.030 | 0.8037 | -0.128 | 0.3653 |
|  | **rpfE** | **Rv2450c** | -0.096 | 0.464 | -0.053 | 0.7153 | -0.107 | 0.4347 |

**ALL DS SAMPLES**

|  |  |  | **MPN^Max^/CFU**  **(n=35)** | | | **MPN^+CF^/CFU**  **(n=35)** | | | **MPN^-CF^/CFU**  **(n=35)** | | |
| --- | --- | --- | --- | --- | --- | --- | --- | --- | --- | --- | --- |
|  | **Gene** | **Rv#** | **Spearman correlation** | **p-val** | **Adj p-val** | **Spearman correlation** | **p-val** | **Adj p-val** | **Spearman correlation** | **p-val** | **Adj p-val** |
| **Downregulated**  **DD Mtb candidates** | **icl1** | **Rv0467** | -0.552 | 0.0002 | 0.0027 | -0.536 | 0.0003 | 0.0029 | -0.519 | 0.0005 | 0.0071 |
|  | **carD** | **Rv3583c** | -0.500 | 0.0009 | 0.003 | -0.497 | 0.0009 | 0.0033 | -0.466 | 0.0021 | 0.0098 |
|  | **vapB10** | **Rv1398c** | -0.487 | 0.0012 | 0.0034 | -0.483 | 0.0014 | 0.0038 | -0.446 | 0.0033 | 0.0114 |
|  | **ppsA** | **Rv2931** | -0.515 | 0.0005 | 0.003 | -0.512 | 0.0005 | 0.0029 | -0.464 | 0.0020 | 0.0098 |
|  | **hspX** | **Rv2031c** | -0.366 | 0.0175 | 0.0307 | -0.417 | 0.0064 | 0.0111 | -0.304 | 0.0508 | 0.0889 |
|  | **Rv1738** | **Rv1738** | -0.350 | 0.0229 | 0.0357 | -0.427 | 0.0048 | 0.0111 | -0.290 | 0.0628 | 0.0925 |
|  | **tatA** | **Rv2094c** | -0.500 | 0.0008 | 0.003 | -0.507 | 0.0006 | 0.0029 | -0.434 | 0.0041 | 0.0114 |
|  | **whiB1** | **Rv3219** | -0.367 | 0.0174 | 0.0307 | -0.287 | 0.0654 | 0.0763 | -0.366 | 0.0177 | 0.0412 |
|  | **pks15** | **Rv2947c** | -0.396 | 0.0099 | 0.0231 | -0.418 | 0.0063 | 0.0111 | -0.337 | 0.0298 | 0.0595 |
|  | **lldD2** | **Rv1872c** | -0.299 | 0.0549 | 0.0768 | -0.293 | 0.0598 | 0.0761 | -0.287 | 0.0661 | 0.0925 |
| **Upregulated DD Mtb candidates** | **arsC** | **Rv2643** | 0.223 | 0.1561 | 0.1822 | 0.357 | 0.0207 | 0.029 | 0.186 | 0.2383 | 0.2893 |
|  | **lpqX** | **Rv1228** | 0.233 | 0.1368 | 0.1741 | 0.359 | 0.0200 | 0.029 | 0.182 | 0.2480 | 0.2893 |
|  | **ugpC** | **Rv2832c** | -0.112 | 0.4779 | 0.4779 | 0.005 | 0.9755 | 0.9755 | -0.155 | 0.3248 | 0.3248 |
|  | **rpfE** | **Rv2450c** | -0.145 | 0.3579 | 0.3854 | -0.032 | 0.8378 | 0.9023 | -0.171 | 0.2777 | 0.2991 |

**ALL DR SAMPLES**

|  | **Gene** | **Rv#** | **MPN^Max^/CFU**  **(n=27)** | | | **MPN^+CF^/CFU**  **(n=27)** | | | **MPN^-CF^/CFU**  **(n=28)** | | |
| --- | --- | --- | --- | --- | --- | --- | --- | --- | --- | --- | --- |
|  |  |  | **Spearman correlation** | **p-val** | **Adj p-val** | **Spearman correlation** | **p-val** | **Adj p-val** | **Spearman correlation** | **p-val** | **Adj p-val** |
| **Downregulated**  **DD Mtb candidates** | **icl1** | **Rv0467** | -0.203 | 0.2986 | 0.9767 | -0.234 | 0.2292 | 0.615 | 0.033 | 0.8651 | 0.9317 |
|  | **carD** | **Rv3583c** | -0.057 | 0.7710 | 0.9767 | -0.187 | 0.3386 | 0.615 | 0.190 | 0.3217 | 0.9317 |
|  | **vapB10** | **Rv1398c** | -0.047 | 0.8118 | 0.9767 | -0.067 | 0.7329 | 0.855 | 0.238 | 0.2131 | 0.9317 |
|  | **ppsA** | **Rv2931** | -0.060 | 0.7607 | 0.9767 | -0.152 | 0.4393 | 0.615 | 0.103 | 0.5949 | 0.9317 |
|  | **hspX** | **Rv2031c** | -0.134 | 0.4947 | 0.9767 | -0.163 | 0.4069 | 0.615 | 0.153 | 0.4259 | 0.9317 |
|  | **Rv1738** | **Rv1738** | -0.193 | 0.3231 | 0.9767 | -0.280 | 0.1492 | 0.615 | -0.007 | 0.9725 | 0.9725 |
|  | **tatA** | **Rv2094c** | -0.074 | 0.7078 | 0.9767 | -0.186 | 0.3429 | 0.615 | 0.097 | 0.6170 | 0.9317 |
|  | **whiB1** | **Rv3219** | 0.037 | 0.8531 | 0.9767 | -0.001 | 0.9989 | 0.9989 | 0.109 | 0.5708 | 0.9317 |
|  | **pks15** | **Rv2947c** | -0.104 | 0.5971 | 0.9767 | -0.213 | 0.2741 | 0.615 | 0.076 | 0.6929 | 0.9317 |
|  | **lldD2** | **Rv1872c** | -0.012 | 0.9523 | 0.9767 | -0.159 | 0.4181 | 0.615 | 0.147 | 0.4441 | 0.9317 |
| **Upregulated DD Mtb candidates** | **arsC** | **Rv2643** | 0.185 | 0.3444 | 0.9767 | 0.192 | 0.3273 | 0.615 | -0.063 | 0.7446 | 0.9317 |
|  | **lpqX** | **Rv1228** | 0.200 | 0.3065 | 0.9767 | 0.254 | 0.1919 | 0.615 | -0.145 | 0.4517 | 0.9317 |
|  | **ugpC** | **Rv2832c** | 0.173 | 0.3771 | 0.9767 | -0.012 | 0.9523 | 0.9989 | -0.043 | 0.8231 | 0.9317 |
|  | **rpfE** | **Rv2450c** | -0.006 | 0.9767 | 0.9767 | -0.084 | 0.6687 | 0.8511 | -0.056 | 0.7718 | 0.9317 |

**ALL W2 SAMPLES (DS & DR)**

|  | **Gene** | **Rv#** | **MPN^Max^/CFU**  **(n=26)** | | | **MPN^+CF^/CFU**  **(n=26)** | | | **MPN^-CF^/CFU**  **(n=26)** | | | |
| --- | --- | --- | --- | --- | --- | --- | --- | --- | --- | --- | --- | --- |
|  |  |  | **Spearman correlation** | **p-val** | **Adj p-val** | **Spearman correlation** | **p-val** | **Adj p-val** | **Spearman correlation** | **p-val** | **Adj p-val** |  |
| **Downregulated**  **DD Mtb candidates** | **icl1** | **Rv0467** | -0.591 | 0.0018 | 0.0252 | -0.581 | 0.0022 | 0.0313 | -0.593 | 0.0017 | 0.0241 |  |
|  | **carD** | **Rv3583c** | -0.390 | 0.0498 | 0.2322 | -0.393 | 0.0481 | 0.2243 | -0.314 | 0.1190 | 0.5553 |  |
|  | **vapB10** | **Rv1398c** | -0.364 | 0.0682 | 0.2387 | -0.335 | 0.0944 | 0.3015 | -0.439 | 0.0260 | 0.1821 |  |
|  | **hspX** | **Rv2031c** | -0.284 | 0.1592 | 0.3873 | -0.323 | 0.1077 | 0.3015 | -0.101 | 0.6227 | 0.8244 |  |
|  | **ppsA** | **Rv2931** | -0.280 | 0.1660 | 0.3873 | -0.279 | 0.1682 | 0.3924 | -0.129 | 0.5300 | 0.8244 |  |
|  | **Rv1738** | **Rv1738** | -0.149 | 0.4663 | 0.7068 | -0.151 | 0.4611 | 0.6879 | -0.094 | 0.6477 | 0.8244 |  |
|  | **tatA** | **Rv2094c** | -0.109 | 0.5958 | 0.7068 | -0.141 | 0.4914 | 0.6879 | 0.111 | 0.5900 | 0.8244 |  |
|  | **whiB1** | **Rv3219** | -0.123 | 0.5465 | 0.7068 | -0.097 | 0.6371 | 0.8108 | -0.130 | 0.5243 | 0.8244 |  |
|  | **pks15** | **Rv2947c** | 0.093 | 0.6515 | 0.7068 | 0.050 | 0.8073 | 0.8594 | 0.169 | 0.4068 | 0.8244 |  |
|  | **lldD2** | **Rv1872c** | 0.176 | 0.3879 | 0.6788 | 0.192 | 0.3446 | 0.6030 | 0.069 | 0.7381 | 0.8611 |  |
| **Upregulated DD Mtb candidates** | **arsC** | **Rv2643** | 0.068 | 0.7407 | 0.7407 | 0.067 | 0.7457 | 0.8594 | -0.106 | 0.6062 | 0.8244 |  |
|  | **lpqX** | **Rv1228** | 0.190 | 0.3498 | 0.6788 | 0.201 | 0.3241 | 0.6030 | -0.023 | 0.9122 | 0.9122 |  |
|  | **ugpC** | **Rv2832c** | 0.419 | 0.0330 | 0.2312 | 0.457 | 0.0189 | 0.1321 | 0.124 | 0.5446 | 0.8244 |  |
|  | **rpfE** | **Rv2450c** | -0.091 | 0.6563 | 0.7068 | -0.037 | 0.8594 | 0.8594 | -0.035 | 0.8647 | 0.9122 |  |

**DS W2 SAMPLES**

|  |  |  | **MPN^Max^/CFU**  **(n=15)** | | | **MPN^+CF^/CFU**  **(n=15)** | | | **MPN^-CF^/CFU**  **(n=15)** | | |
| --- | --- | --- | --- | --- | --- | --- | --- | --- | --- | --- | --- |
|  | **Gene** | **Rv#** | **Spearman correlation** | **p-val** | **Adj p-val** | **Spearman correlation** | **p-val** | **Adj p-val** | **Spearman correlation** | **p-val** | **Adj p-val** |
| **Downregulated**  **DD Mtb candidates** | **icl1** | **Rv0467** | -0.586 | 0.0243 | 0.3322 | -0.500 | 0.0602 | 0.5292 | -0.661 | 0.0090 | 0.1266 |
|  | **carD** | **Rv3583c** | -0.129 | 0.6482 | 0.9849 | -0.032 | 0.9132 | 0.9132 | -0.150 | 0.5934 | 0.8495 |
|  | **vapB10** | **Rv1398c** | -0.129 | 0.6482 | 0.9849 | 0.036 | 0.9031 | 0.9132 | -0.250 | 0.3677 | 0.8495 |
|  | **hspX** | **Rv2031c** | 0.182 | 0.5151 | 0.9849 | 0.139 | 0.6206 | 0.9132 | 0.179 | 0.5235 | 0.8495 |
|  | **ppsA** | **Rv2931** | -0.519 | 0.0475 | 0.3322 | -0.472 | 0.0756 | 0.5292 | -0.389 | 0.1516 | 0.8495 |
|  | **Rv1738** | **Rv1738** | 0.038 | 0.8944 | 0.9849 | 0.095 | 0.7370 | 0.9132 | 0.054 | 0.8495 | 0.8495 |
|  | **tatA** | **Rv2094c** | 0.005 | 0.9849 | 0.9849 | -0.113 | 0.6895 | 0.9132 | 0.075 | 0.7903 | 0.8495 |
|  | **whiB1** | **Rv3219** | -0.032 | 0.9132 | 0.9849 | 0.104 | 0.7144 | 0.9132 | -0.114 | 0.6858 | 0.8495 |
|  | **pks15** | **Rv2947c** | -0.039 | 0.8929 | 0.9849 | -0.129 | 0.6482 | 0.9132 | -0.086 | 0.7630 | 0.8495 |
|  | **lldD2** | **Rv1872c** | 0.189 | 0.4984 | 0.9849 | 0.207 | 0.4578 | 0.9132 | 0.179 | 0.5235 | 0.8495 |
| **Upregulated DD Mtb candidates** | **arsC** | **Rv2643** | 0.029 | 0.9234 | 0.9849 | 0.157 | 0.5756 | 0.9132 | -0.071 | 0.8025 | 0.8495 |
|  | **lpqX** | **Rv1228** | -0.021 | 0.9438 | 0.9849 | 0.100 | 0.7241 | 0.9132 | -0.129 | 0.6482 | 0.8495 |
|  | **ugpC** | **Rv2832c** | 0.254 | 0.3607 | 0.9849 | 0.354 | 0.1963 | 0.9132 | 0.125 | 0.6575 | 0.8495 |
|  | **rpfE** | **Rv2450c** | -0.068 | 0.8124 | 0.9849 | 0.043 | 0.8828 | 0.9132 | -0.121 | 0.6669 | 0.8495 |

**DR W2 SAMPLES**

|  |  |  | **MPN^Max^/CFU**  **(n=11)** | | | **MPN^+CF^/CFU**  **(n=11)** | | | **MPN^-CF^/CFU**  **(n=11)** | | |
| --- | --- | --- | --- | --- | --- | --- | --- | --- | --- | --- | --- |
|  | **Gene** | **Rv#** | **Spearman correlation** | **p-val** | **Adj p-val** | **Spearman correlation** | **p-val** | **Adj p-val** | **Spearman correlation** | **p-val** | **Adj p-val** |
| **Downregulated**  **DD Mtb candidates** | **icl1** | **Rv0467** | -0.336 | 0.3130 | 0.8658 | -0.382 | 0.2484 | 0.8383 | 0.118 | 0.7343 | 0.9892 |
|  | **carD** | **Rv3583c** | -0.227 | 0.5031 | 0.8804 | -0.345 | 0.2994 | 0.8383 | 0.400 | 0.2250 | 0.9892 |
|  | **vapB10** | **Rv1398c** | -0.164 | 0.6339 | 0.9244 | -0.264 | 0.4345 | 0.8804 | 0.336 | 0.3130 | 0.9892 |
|  | **hspX** | **Rv2031c** | -0.427 | 0.1926 | 0.8658 | -0.436 | 0.1825 | 0.8383 | 0.227 | 0.5031 | 0.9892 |
|  | **ppsA** | **Rv2931** | -0.132 | 0.6986 | 0.9244 | -0.178 | 0.6012 | 0.9352 | 0.077 | 0.8209 | 0.9892 |
|  | **Rv1738** | **Rv1738** | -0.300 | 0.3711 | 0.8658 | -0.364 | 0.2732 | 0.8383 | 0.082 | 0.8177 | 0.9892 |
|  | **tatA** | **Rv2094c** | -0.345 | 0.2994 | 0.8658 | -0.245 | 0.4682 | 0.8804 | -0.018 | 0.9676 | 0.9892 |
|  | **whiB1** | **Rv3219** | 0.055 | 0.8815 | 0.9244 | 0.000 | 1.0000 | 1 | 0.227 | 0.5031 | 0.9892 |
|  | **pks15** | **Rv2947c** | -0.036 | 0.9244 | 0.9244 | -0.100 | 0.7757 | 1 | -0.009 | 0.9892 | 0.9892 |
|  | **lldD2** | **Rv1872c** | -0.064 | 0.8601 | 0.9244 | 0.018 | 0.9676 | 1 | -0.300 | 0.3711 | 0.9892 |
| **Upregulated DD Mtb candidates** | **arsC** | **Rv2643** | 0.245 | 0.4682 | 0.8804 | 0.064 | 0.8601 | 1 | 0.191 | 0.5763 | 0.9892 |
|  | **lpqX** | **Rv1228** | 0.345 | 0.2994 | 0.8658 | 0.227 | 0.5031 | 0.8804 | 0.136 | 0.6935 | 0.9892 |
|  | **ugpC** | **Rv2832c** | 0.500 | 0.1214 | 0.8658 | 0.455 | 0.1634 | 0.8383 | -0.055 | 0.8815 | 0.9892 |
|  | **rpfE** | **Rv2450c** | -0.109 | 0.7549 | 0.9244 | -0.064 | 0.8601 | 1 | 0.145 | 0.6734 | 0.9892 |

**ALL D0 SAMPLES (DS & DR)**

|  | **Gene** | **Rv#** | **MPN^Max^/CFU**  **(n=36)** | | | **MPN^+CF^/CFU**  **(n=36)** | | | **MPN^-CF^/CFU**  **(n=37)** | | | |
| --- | --- | --- | --- | --- | --- | --- | --- | --- | --- | --- | --- | --- |
|  |  |  | **Spearman correlation** | **p-val** | **Adj p-val** | **Spearman correlation** | **p-val** | **Adj p-val** | **Spearman correlation** | **p-val** | **Adj p-val** |  |
| **Downregulated**  **DD Mtb candidates** | **icl1** | **Rv0467** | 0.054 | 0.7558 | 0.9005 | 0.142 | 0.4061 | 0.5942 | -0.130 | 0.4411 | 0.7881 |  |
|  | **carD** | **Rv3583c** | 0.022 | 0.9005 | 0.9005 | 0.113 | 0.5093 | 0.5942 | -0.175 | 0.2991 | 0.7881 |  |
|  | **vapB10** | **Rv1398c** | 0.099 | 0.5650 | 0.8666 | 0.116 | 0.4987 | 0.5942 | 0.101 | 0.5497 | 0.7881 |  |
|  | **hspX** | **Rv2031c** | 0.085 | 0.6190 | 0.8666 | 0.126 | 0.4639 | 0.5942 | -0.023 | 0.8914 | 0.9292 |  |
|  | **ppsA** | **Rv2931** | 0.114 | 0.5073 | 0.8666 | 0.239 | 0.1608 | 0.5251 | -0.099 | 0.5619 | 0.7881 |  |
|  | **Rv1738** | **Rv1738** | 0.108 | 0.5298 | 0.8666 | 0.015 | 0.9302 | 0.9636 | 0.015 | 0.9292 | 0.9292 |  |
|  | **tatA** | **Rv2094c** | 0.048 | 0.7810 | 0.9005 | 0.156 | 0.3606 | 0.5942 | -0.207 | 0.2177 | 0.7881 |  |
|  | **whiB1** | **Rv3219** | 0.121 | 0.4816 | 0.8666 | 0.261 | 0.1244 | 0.5251 | -0.168 | 0.3180 | 0.7881 |  |
|  | **pks15** | **Rv2947c** | 0.249 | 0.1434 | 0.5802 | 0.317 | 0.0597 | 0.4182 | 0.035 | 0.8375 | 0.9292 |  |
|  | **lldD2** | **Rv1872c** | 0.236 | 0.1658 | 0.5802 | 0.331 | 0.0492 | 0.4182 | 0.045 | 0.7918 | 0.9292 |  |
| **Upregulated DD Mtb candidates** | **arsC** | **Rv2643** | -0.311 | 0.0649 | 0.5802 | -0.224 | 0.1875 | 0.5251 | -0.262 | 0.1165 | 0.7881 |  |
|  | **lpqX** | **Rv1228** | -0.242 | 0.1550 | 0.5802 | -0.162 | 0.3446 | 0.5942 | -0.190 | 0.2589 | 0.7881 |  |
|  | **ugpC** | **Rv2832c** | 0.090 | 0.6001 | 0.8666 | 0.158 | 0.3567 | 0.5942 | -0.098 | 0.5629 | 0.7881 |  |
|  | **rpfE** | **Rv2450c** | -0.030 | 0.8626 | 0.9005 | 0.008 | 0.9636 | 0.9636 | -0.154 | 0.3626 | 0.7881 |  |

**DS D0 SAMPLES**

|  | **Gene** | **Rv#** | **MPN^Max^/CFU**  **(n=20)** | | | **MPN^+CF^/CFU**  **(n=20)** | | | **MPN^-CF^/CFU**  **(n=20)** | | | |
| --- | --- | --- | --- | --- | --- | --- | --- | --- | --- | --- | --- | --- |
|  |  |  | **Spearman correlation** | **p-val** | **Adj p-val** | **Spearman correlation** | **p-val** | **Adj p-val** | **Spearman correlation** | **p-val** | **Adj p-val** |  |
| **Downregulated**  **DD Mtb candidates** | **icl1** | **Rv0467** | 0.134 | 0.5725 | 0.8015 | 0.421 | 0.0658 | 0.2286 | -0.072 | 0.7625 | 0.8913 |  |
|  | **carD** | **Rv3583c** | -0.140 | 0.5551 | 0.8015 | 0.159 | 0.5005 | 0.5839 | -0.382 | 0.0974 | 0.4545 |  |
|  | **vapB10** | **Rv1398c** | -0.083 | 0.7287 | 0.9274 | 0.104 | 0.6626 | 0.6626 | -0.071 | 0.7673 | 0.8913 |  |
|  | **ppsA** | **Rv2931** | 0.156 | 0.5101 | 0.8015 | 0.405 | 0.0762 | 0.2286 | -0.047 | 0.8427 | 0.8913 |  |
|  | **hspX** | **Rv2031c** | 0.296 | 0.2041 | 0.4763 | 0.359 | 0.1201 | 0.2803 | 0.146 | 0.5380 | 0.7954 |  |
|  | **Rv1738** | **Rv1738** | 0.430 | 0.0598 | 0.2789 | 0.272 | 0.2447 | 0.3426 | 0.403 | 0.0792 | 0.4545 |  |
|  | **tatA** | **Rv2094c** | -0.143 | 0.5465 | 0.8015 | 0.131 | 0.5812 | 0.6259 | -0.277 | 0.2367 | 0.6628 |  |
|  | **whiB1** | **Rv3219** | 0.051 | 0.8313 | 0.9698 | 0.308 | 0.1857 | 0.3249 | -0.135 | 0.5681 | 0.7954 |  |
|  | **pks15** | **Rv2947c** | 0.394 | 0.0867 | 0.3033 | 0.632 | 0.0035 | 0.0244 | 0.188 | 0.4257 | 0.7954 |  |
|  | **lldD2** | **Rv1872c** | 0.310 | 0.1835 | 0.4763 | 0.666 | 0.0018 | 0.0244 | 0.033 | 0.8913 | 0.8913 |  |
| **Upregulated DD Mtb candidates** | **arsC** | **Rv2643** | -0.603 | 0.0058 | 0.0809 | -0.335 | 0.1485 | 0.2969 | -0.498 | 0.0271 | 0.379 |  |
|  | **lpqX** | **Rv1228** | -0.451 | 0.0474 | 0.2789 | -0.277 | 0.2367 | 0.3426 | -0.326 | 0.1602 | 0.5608 |  |
|  | **ugpC** | **Rv2832c** | 0.014 | 0.9569 | 0.9924 | 0.400 | 0.0816 | 0.2286 | -0.179 | 0.4486 | 0.7954 |  |
|  | **rpfE** | **Rv2450c** | -0.003 | 0.9924 | 0.9924 | 0.226 | 0.3374 | 0.4294 | -0.138 | 0.5594 | 0.7954 |  |

**DR D0 SAMPLES**

|  |  |  | **MPN^Max^/CFU**  **(n=16)** | | | **MPN^+CF^/CFU**  **(n=16)** | | | **MPN^-CF^/CFU**  **(n=17)** | | |
| --- | --- | --- | --- | --- | --- | --- | --- | --- | --- | --- | --- |
|  | **Gene** | **Rv#** | **Spearman correlation** | **p-val** | **Adj p-val** | **Spearman correlation** | **p-val** | **Adj p-val** | **Spearman correlation** | **p-val** | **Adj p-val** |
| **Downregulated**  **DD Mtb candidates** | **icl1** | **Rv0467** | -0.162 | 0.5486 | 0.8968 | -0.156 | 0.5635 | 0.9781 | -0.206 | 0.4264 | 0.9837 |
|  | **carD** | **Rv3583c** | 0.188 | 0.4839 | 0.8968 | 0.121 | 0.6564 | 0.9781 | 0.076 | 0.7729 | 0.9837 |
|  | **vapB10** | **Rv1398c** | 0.291 | 0.2731 | 0.8968 | 0.159 | 0.5560 | 0.9781 | 0.270 | 0.2942 | 0.9837 |
|  | **ppsA** | **Rv2931** | 0.132 | 0.6248 | 0.8968 | 0.138 | 0.6092 | 0.9781 | -0.164 | 0.5276 | 0.9837 |
|  | **hspX** | **Rv2031c** | 0.194 | 0.4700 | 0.8968 | 0.303 | 0.2534 | 0.9781 | -0.015 | 0.9585 | 0.9887 |
|  | **Rv1738** | **Rv1738** | 0.062 | 0.8222 | 0.9039 | -0.012 | 0.9694 | 0.9781 | -0.127 | 0.6254 | 0.9837 |
|  | **tatA** | **Rv2094c** | 0.350 | 0.1841 | 0.8968 | 0.238 | 0.3729 | 0.9781 | -0.118 | 0.6526 | 0.9837 |
|  | **whiB1** | **Rv3219** | 0.259 | 0.3319 | 0.8968 | 0.203 | 0.4496 | 0.9781 | -0.127 | 0.6254 | 0.9837 |
|  | **pks15** | **Rv2947c** | 0.056 | 0.8393 | 0.9039 | 0.009 | 0.9781 | 0.9781 | -0.120 | 0.6458 | 0.9837 |
|  | **lldD2** | **Rv1872c** | 0.353 | 0.1802 | 0.8968 | 0.062 | 0.8222 | 0.9781 | 0.245 | 0.3417 | 0.9837 |
| **Upregulated DD Mtb candidates** | **arsC** | **Rv2643** | 0.126 | 0.6406 | 0.8968 | -0.085 | 0.7546 | 0.9781 | -0.007 | 0.9811 | 0.9887 |
|  | **lpqX** | **Rv1228** | 0.029 | 0.9136 | 0.9136 | -0.027 | 0.9223 | 0.9781 | -0.088 | 0.7357 | 0.9837 |
|  | **ugpC** | **Rv2832c** | 0.138 | 0.6092 | 0.8968 | -0.232 | 0.3852 | 0.9781 | -0.005 | 0.9887 | 0.9887 |
|  | **rpfE** | **Rv2450c** | -0.068 | 0.8051 | 0.9039 | -0.226 | 0.3976 | 0.9781 | -0.135 | 0.6053 | 0.9837 |
