# Supplementary material for: Transcriptional Biomarkers of Differentially Detectable Mycobacterium tuberculosis in Patient Sputum
Source: mBio. 2022 Nov 3;13(6):e02701-22. doi: 10.1128/mbio.02701-22 (PMC9765512; doi:10.1128/mbio.02701-22)
Supplement: FIG S3 [file mbio.02701-22-s0003.pdf]

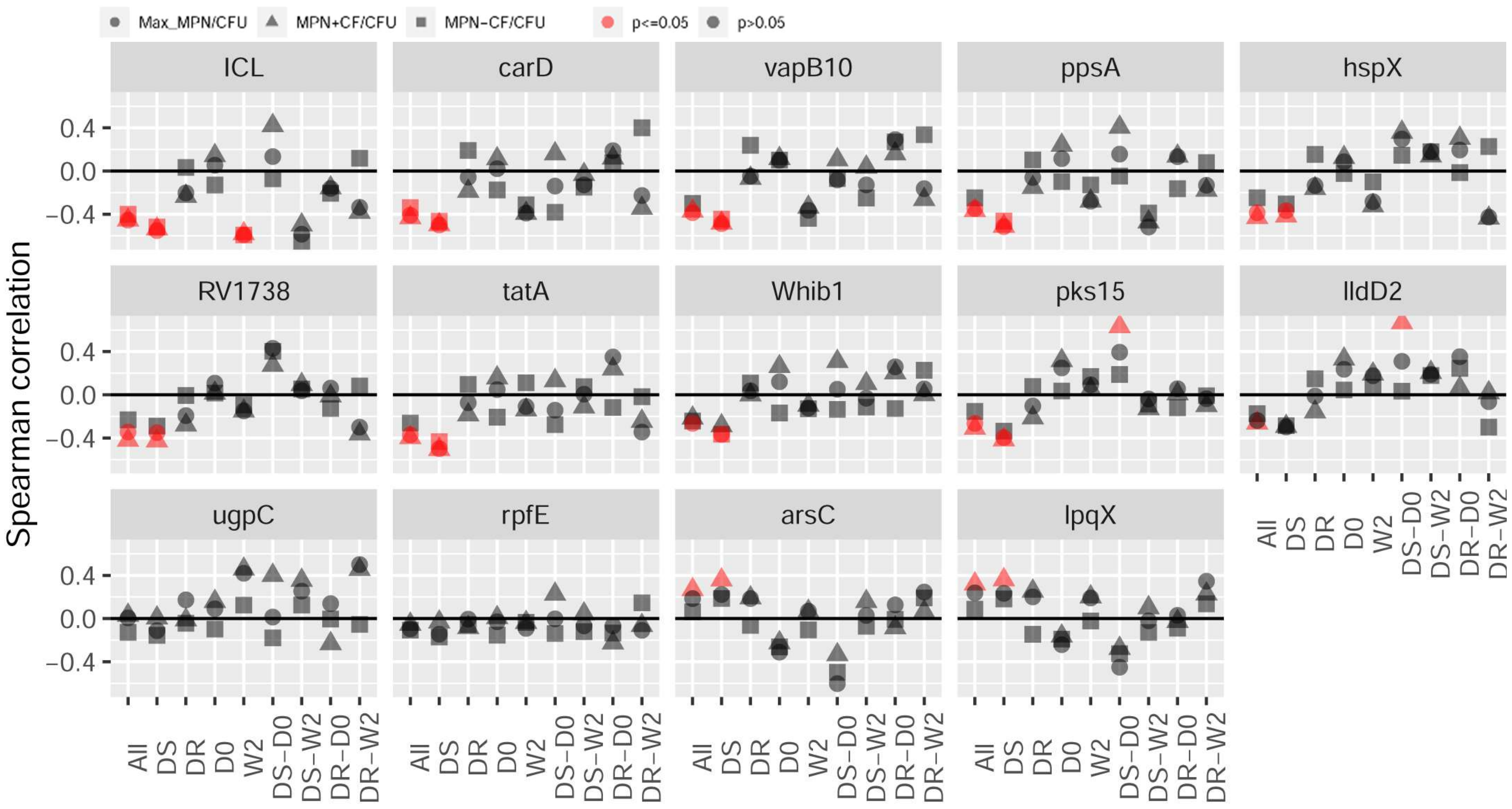

Max_MPN/CFU
MPN+CF/CFU
MPN−CF/CFU
p<=0.05
p>0.05
ICL
carD
vapB10
ppsA
hspX
RV1738
tatA
Whib1
pks15
lldD2
ugpC
rpfE
arsC
lpqX
Spearman correlation
0.4
0.0
−0.4
All
DS
DR
D0
W2
DS−D0
DS−W2
DR−D0
DR−W2

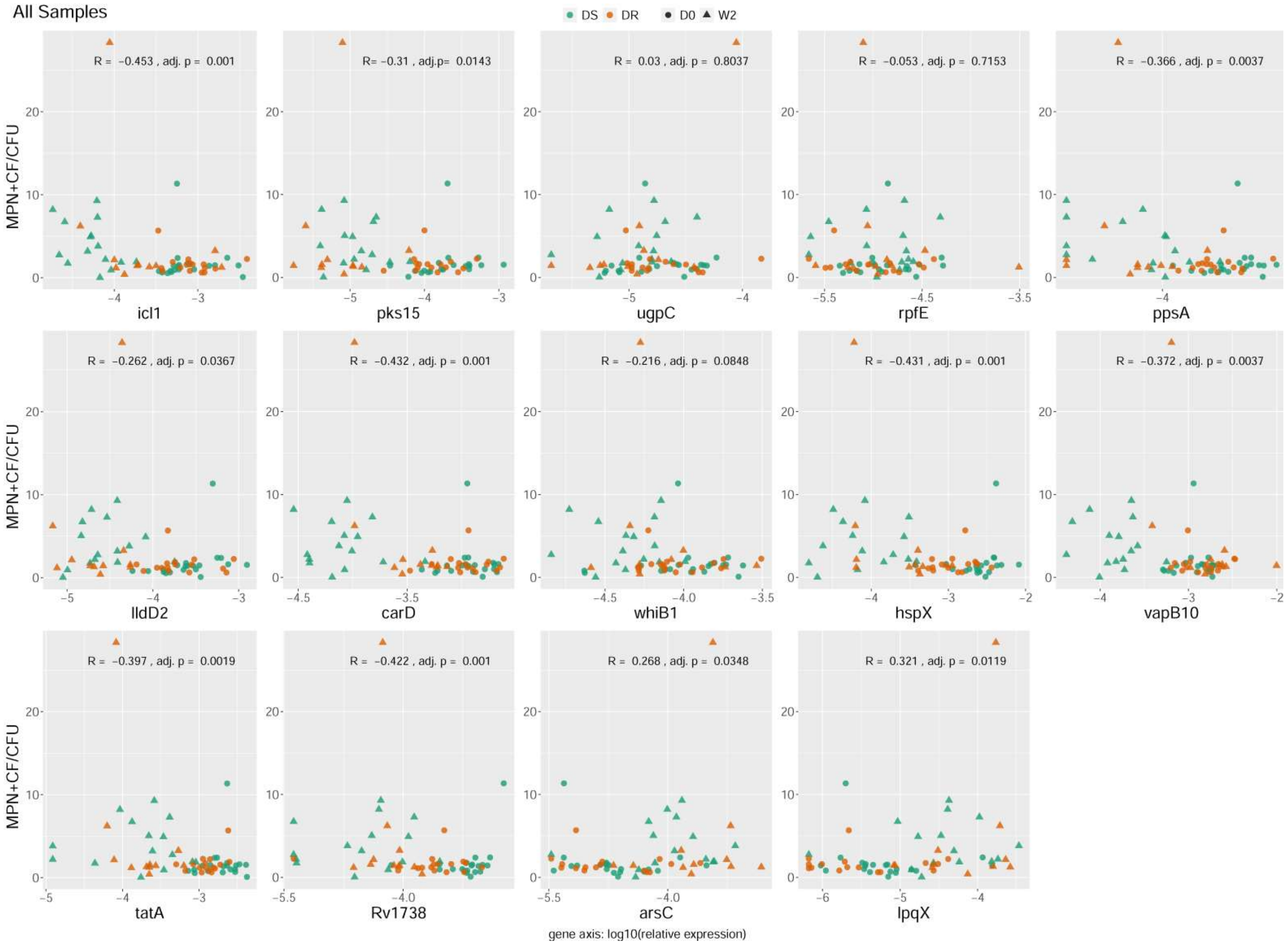
All Samples
DS
DR
D0
W2
MPN+CF/CFU
R = −0.453 , adj. p = 0.001
icl1
R= −0.31 , adj.p= 0.0143
pks15
R = 0.03 , adj. p = 0.8037
ugpC
R = −0.053 , adj. p = 0.7153
rpfE
R = −0.366 , adj. p = 0.0037
ppsA
R = −0.262 , adj. p = 0.0367
lldD2
R = −0.432 , adj. p = 0.001
carD
R = −0.216 , adj. p = 0.0848
whiB1
R = −0.431 , adj. p = 0.001
hspX
R = −0.372 , adj. p = 0.0037
vapB10
R = −0.397 , adj. p = 0.0019
tatA
R = −0.422 , adj. p = 0.001
Rv1738
R = 0.268 , adj. p = 0.0348
arsC
R = 0.321 , adj. p = 0.0119
lpqX
gene axis: log10(relative expression)

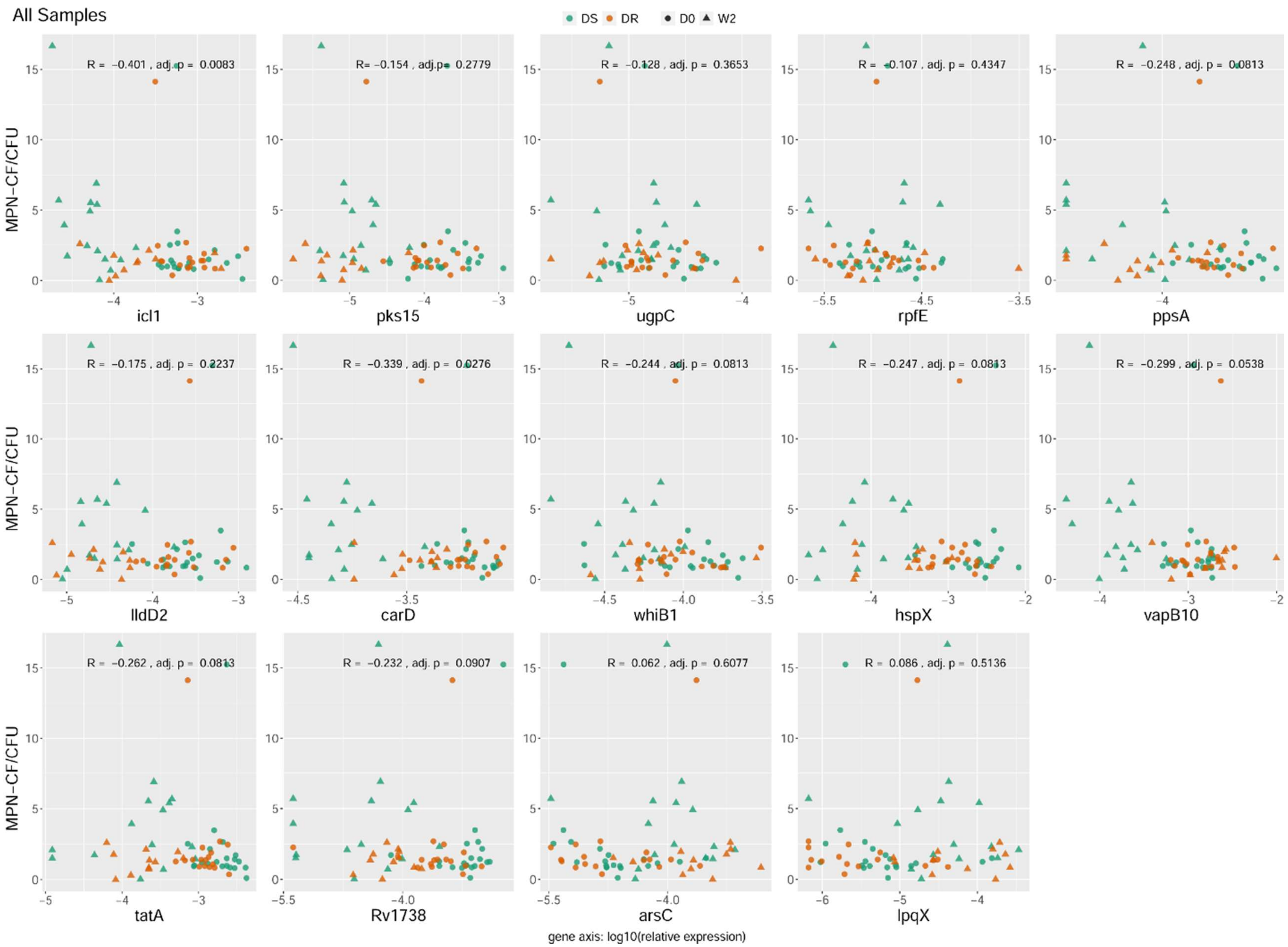
All Samples
DS DR D0 W2
MPN−CF/CFU
R = −0.401 , adj. p = 0.0083
icl1
R= −0.154 , adj.p= 0.2779
pks15
R = −0.128 , adj. p = 0.3653
ugpC
R = 0.107 , adj. p = 0.4347
rpfE
R = −0.248 , adj. p = 0.0813
ppsA
R = −0.175 , adj. p = 0.2237
lldD2
R= −0.339 , adj. p = 0.0276
carD
R = −0.244 , adj. p = 0.0813
whiB1
R = −0.247 , adj. p = 0.0813
hspX
R = −0.299 , adj. p = 0.0538
vapB10
R = −0.262 , adj. p = 0.0813
tatA
R = −0.232 , adj. p = 0.0907
Rv1738
R = 0.062 , adj. p = 0.6077
arsC
R = 0.086 , adj. p = 0.5136
lpqX
gene axis: log10(relative expression)

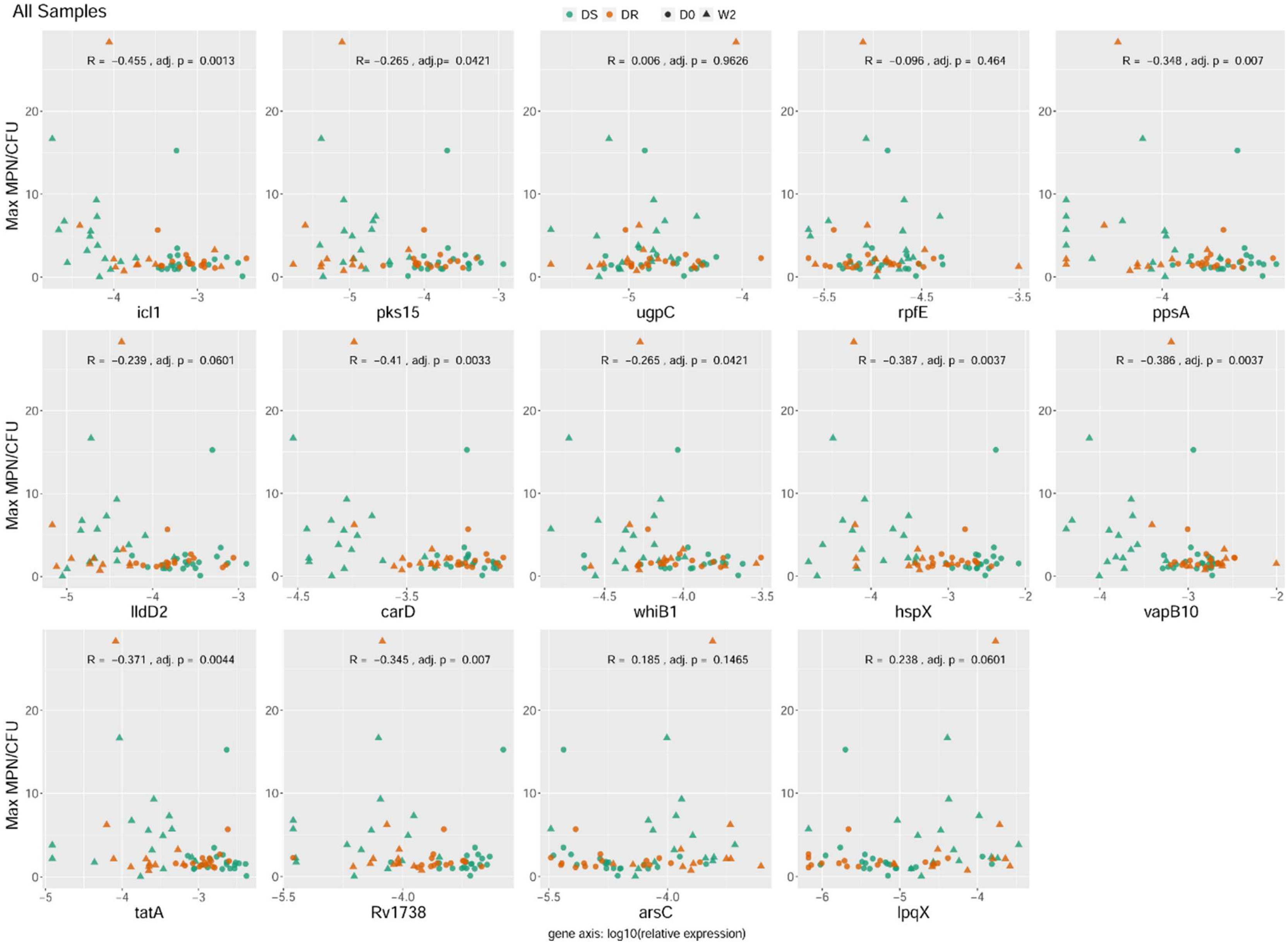
All Samples
DS DR D0 W2
Max MPN/CFU
R = −0.455 , adj. p = 0.0013
icl1
R= −0.265 , adj.p= 0.0421
pks15
R = 0.006 , adj. p = 0.9626
ugpC
R = −0.096 , adj. p = 0.464
rpfE
R = −0.348 , adj. p = 0.007
ppsA
R = −0.239 , adj. p = 0.0601
lldD2
R = −0.41 , adj. p = 0.0033
carD
R = −0.265 , adj. p = 0.0421
whiB1
R = −0.387 , adj. p = 0.0037
hspX
R = −0.386 , adj. p = 0.0037
vapB10
R = −0.371 , adj. p = 0.0044
tatA
R = −0.345 , adj. p = 0.007
Rv1738
R = 0.185 , adj. p = 0.1465
arsC
R = 0.238 , adj. p = 0.0601
lpqX
gene axis: log10(relative expression)

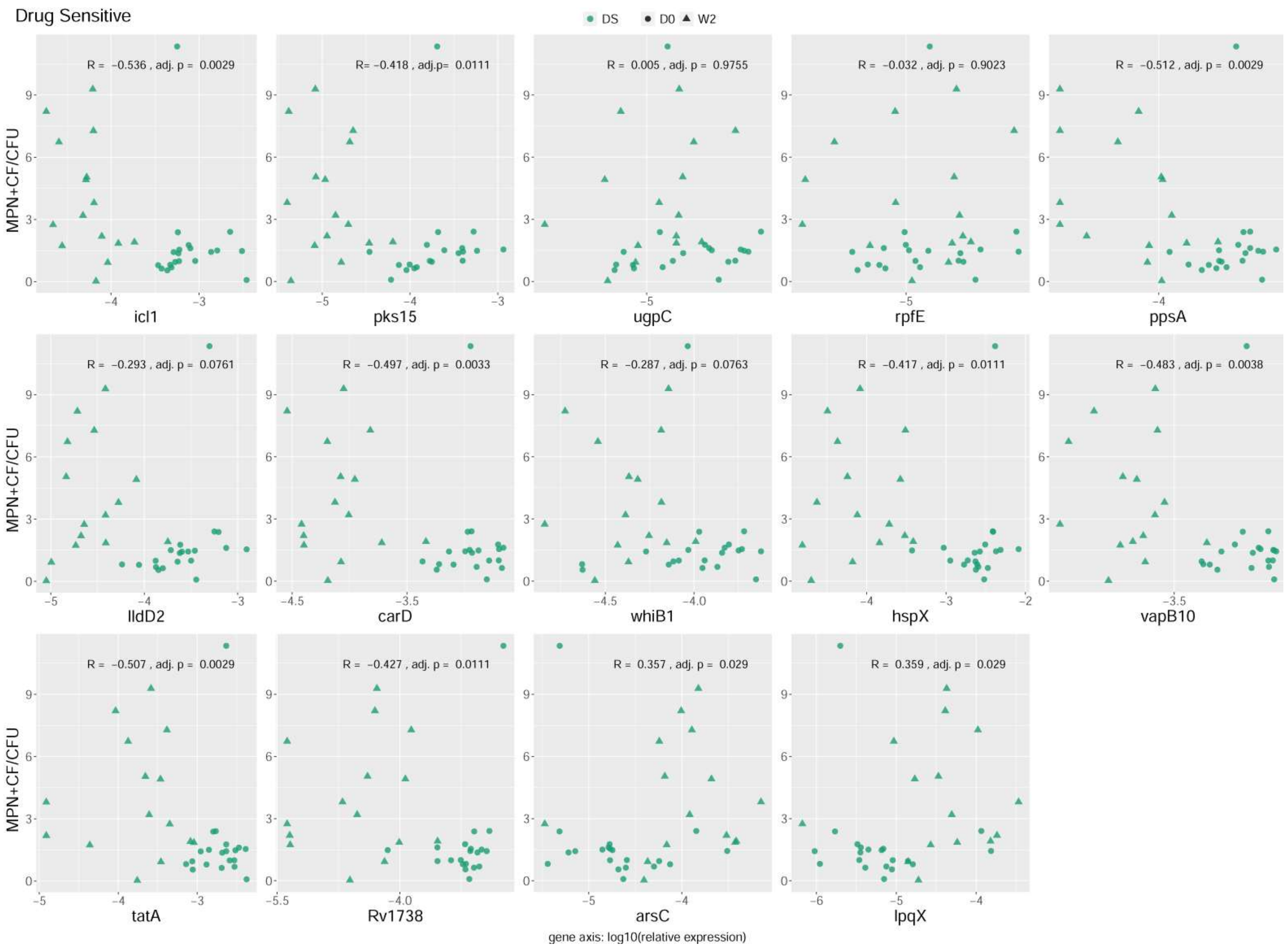
Drug Sensitive
DS
D0
W2
MPN+CF/CFU
R = −0.536 , adj. p = 0.0029
icl1
R= −0.418 , adj.p= 0.0111
pks15
R = 0.005 , adj. p = 0.9755
ugpC
R = −0.032 , adj. p = 0.9023
rpfE
R = −0.512 , adj. p = 0.0029
ppsA
R = −0.293 , adj. p = 0.0761
lldD2
R = −0.497 , adj. p = 0.0033
carD
R = −0.287 , adj. p = 0.0763
whiB1
R = −0.417 , adj. p = 0.0111
hspX
R = −0.483 , adj. p = 0.0038
vapB10
R = −0.507 , adj. p = 0.0029
tatA
R = −0.427 , adj. p = 0.0111
Rv1738
R = 0.357 , adj. p = 0.029
arsC
R = 0.359 , adj. p = 0.029
lpqX
gene axis: log10(relative expression)

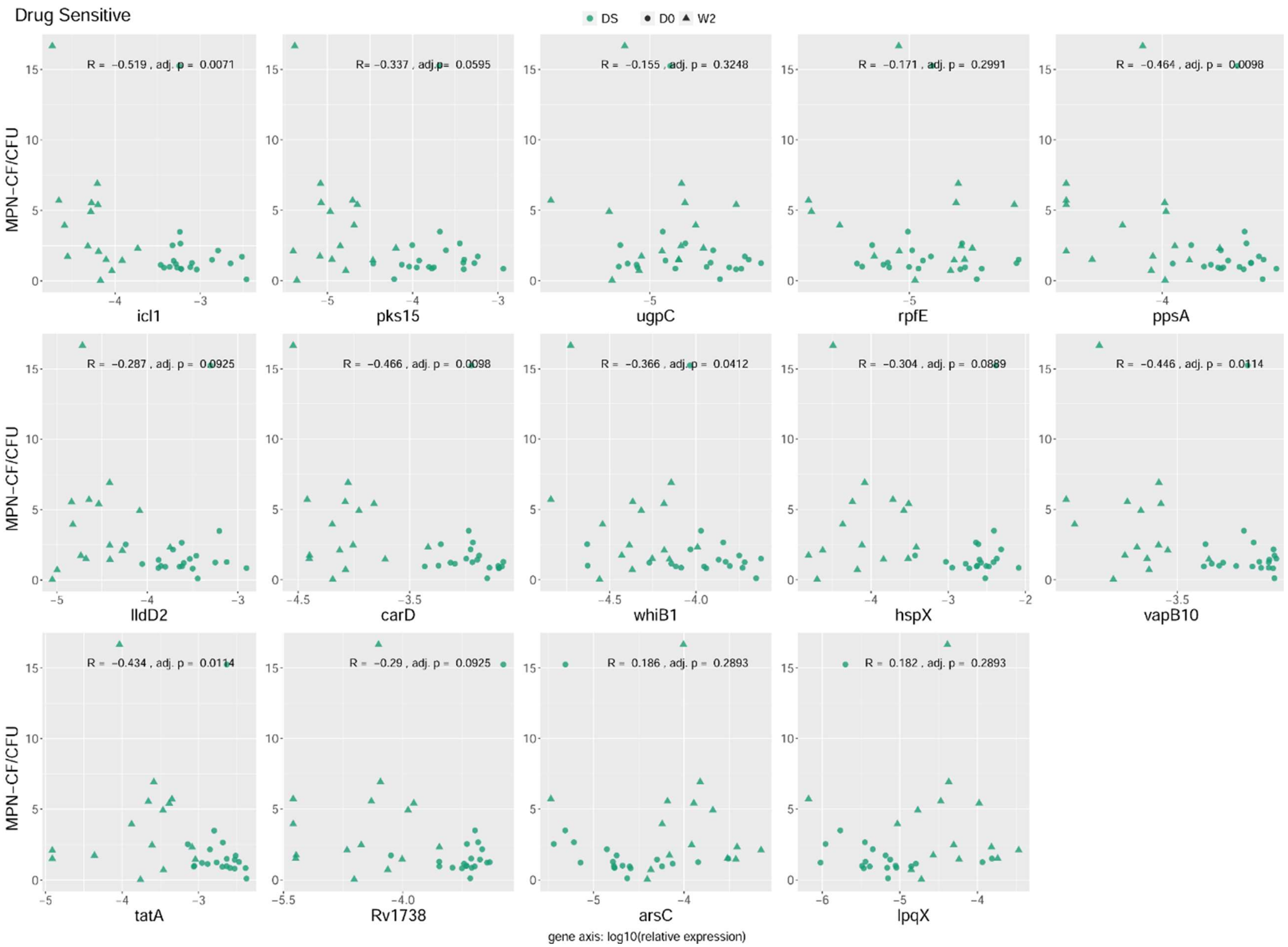

Drug Sensitive
DS
D0
W2
MPN-CF/CFU
R = -0.519 , adj. p = 0.0071
icl1
R= -0.337 , adj. p= 0.0595
pks15
R = -0.155 , adj. p = 0.3248
ugpC
R = -0.171 , adj. p = 0.2991
rpfE
R = -0.464 , adj. p = 0.0098
ppsA
R = -0.287 , adj. p = 0.0925
lldD2
R = -0.466 , adj. p = 0.0098
carD
R = -0.366 , adj. p = 0.0412
whiB1
R = -0.304 , adj. p = 0.0889
hspX
R = -0.446 , adj. p = 0.0114
vapB10
R = -0.434 , adj. p = 0.0114
tatA
R = -0.29 , adj. p = 0.0925
Rv1738
R = 0.186 , adj. p = 0.2893
arsC
R = 0.182 , adj. p = 0.2893
lpqX
gene axis: log10(relative expression)

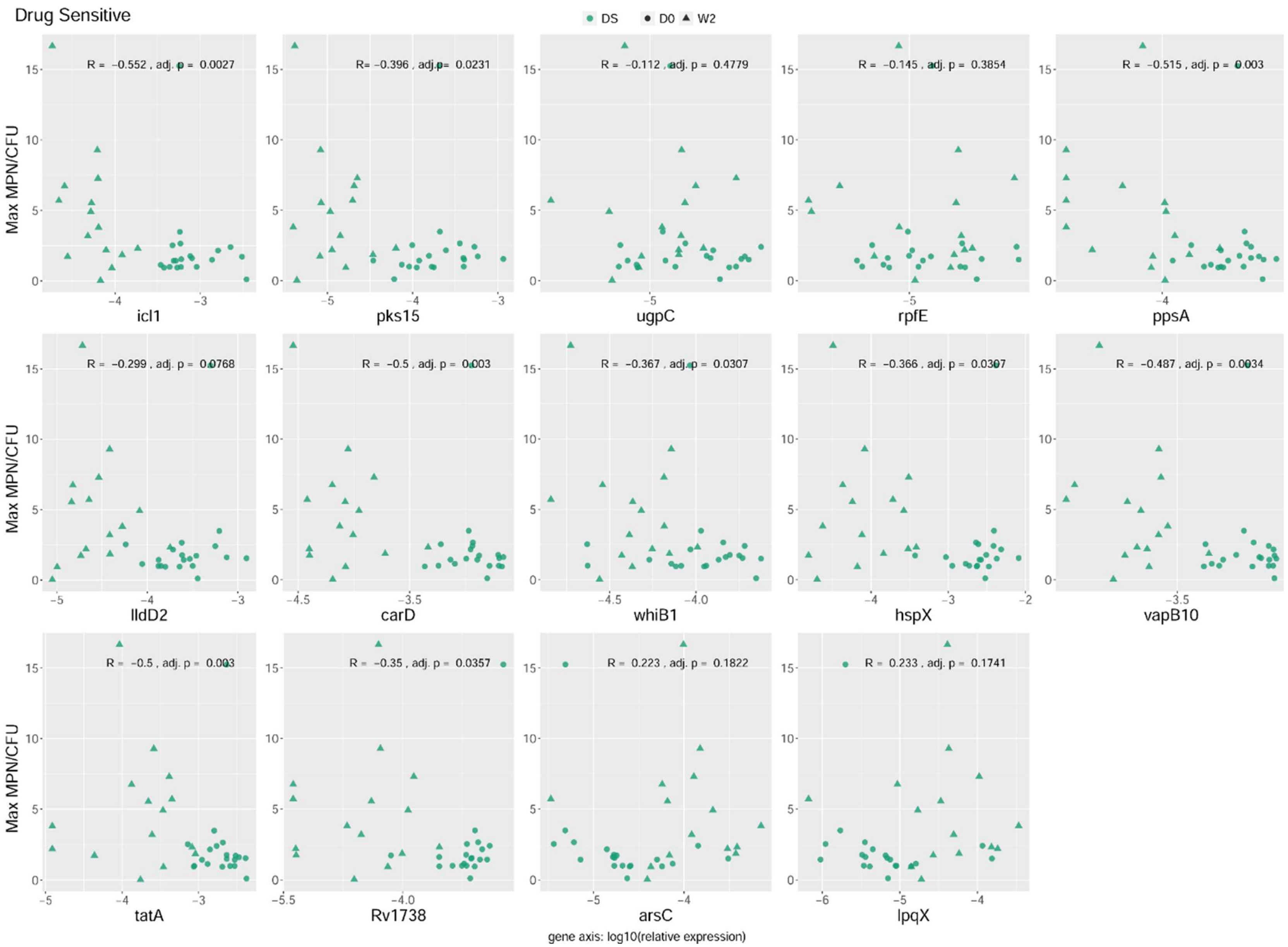
Drug Sensitive
DS
D0
W2
Max MPN/CFU
R = −0.552 , adj. p = 0.0027
icl1
R= −0.396 , adj.p= 0.0231
pks15
R = −0.112 , adj. p = 0.4779
ugpC
R = −0.145 , adj. p = 0.3854
rpfE
R = −0.515 , adj. p = 0.003
ppsA
R = −0.299 , adj. p = 0.0768
lldD2
R = −0.5 , adj. p = 0.003
carD
R = −0.367 , adj. p = 0.0307
whiB1
R = −0.366 , adj. p = 0.0307
hspX
R = −0.487 , adj. p = 0.0034
vapB10
R = −0.5 , adj. p = 0.003
tatA
R = −0.35 , adj. p = 0.0357
Rv1738
R = 0.223 , adj. p = 0.1822
arsC
R = 0.233 , adj. p = 0.1741
lpqX
gene axis: log10(relative expression)

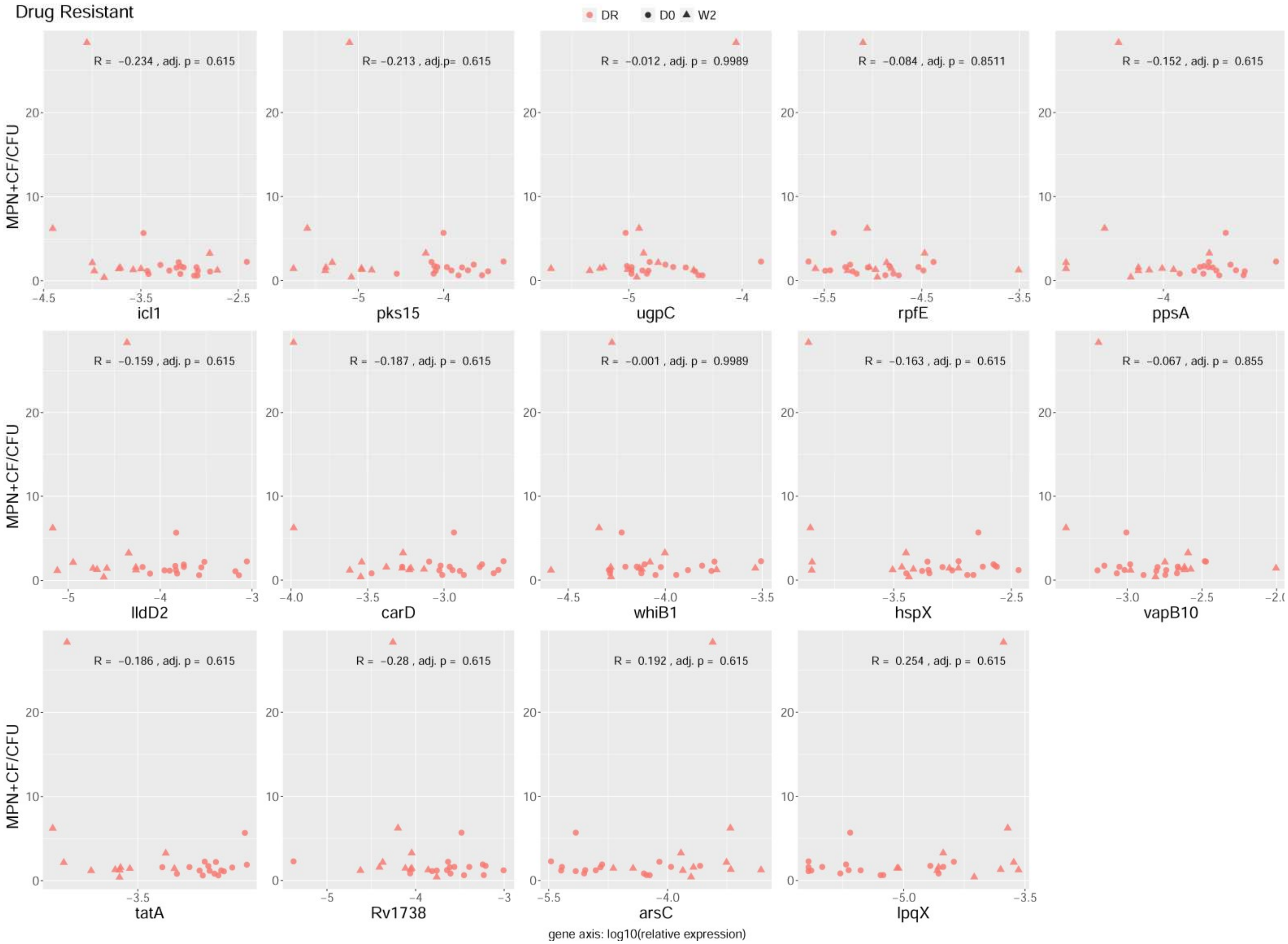

Drug Resistant
DR
D0
W2
MPN+CF/CFU
R = −0.234 , adj. p = 0.615
icl1
R= −0.213 , adj.p= 0.615
pks15
R = −0.012 , adj. p = 0.9989
ugpC
R = −0.084 , adj. p = 0.8511
rpfE
R = −0.152 , adj. p = 0.615
ppsA
R = −0.159 , adj. p = 0.615
lldD2
R = −0.187 , adj. p = 0.615
carD
R = −0.001 , adj. p = 0.9989
whiB1
R = −0.163 , adj. p = 0.615
hspX
R = −0.067 , adj. p = 0.855
vapB10
R = −0.186 , adj. p = 0.615
tatA
R = −0.28 , adj. p = 0.615
Rv1738
R = 0.192 , adj. p = 0.615
arsC
R = 0.254 , adj. p = 0.615
lpqX
gene axis: log10(relative expression)

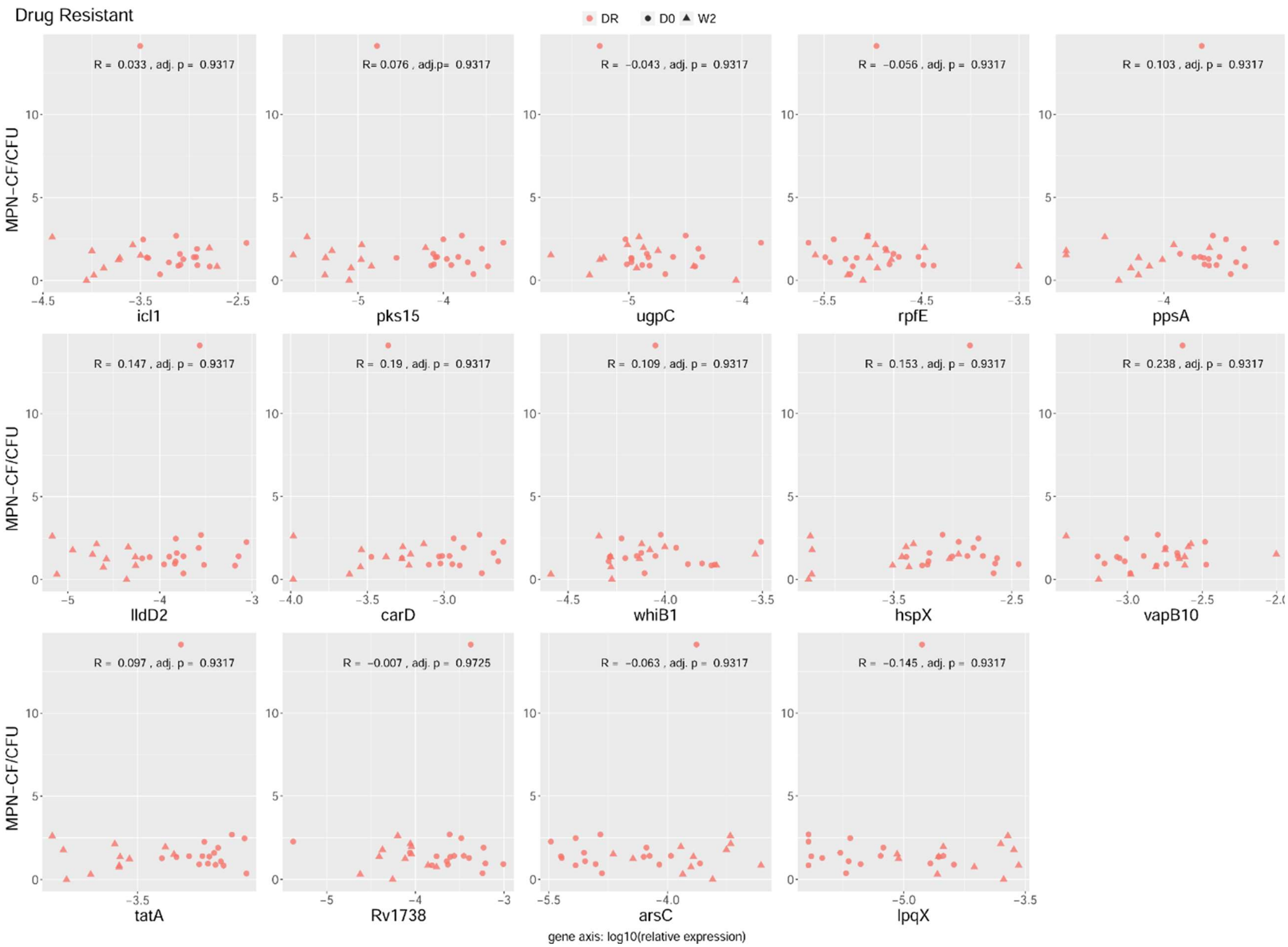

Drug Resistant
DR
D0
W2
MPN−CF/CFU
R = 0.033 , adj. p = 0.9317
icl1
R= 0.076 , adj.p= 0.9317
pks15
R = −0.043 , adj. p = 0.9317
ugpC
R = −0.056 , adj. p = 0.9317
rpfE
R = 0.103 , adj. p = 0.9317
ppsA
R = 0.147 , adj. p = 0.9317
lldD2
R = 0.19 , adj. p = 0.9317
carD
R = 0.109 , adj. p = 0.9317
whiB1
R = 0.153 , adj. p = 0.9317
hspX
R = 0.238 , adj. p = 0.9317
vapB10
R = 0.097 , adj. p = 0.9317
tatA
R = −0.007 , adj. p = 0.9725
Rv1738
R = −0.063 , adj. p = 0.9317
arsC
R = −0.145 , adj. p = 0.9317
lpqX
gene axis: log10(relative expression)

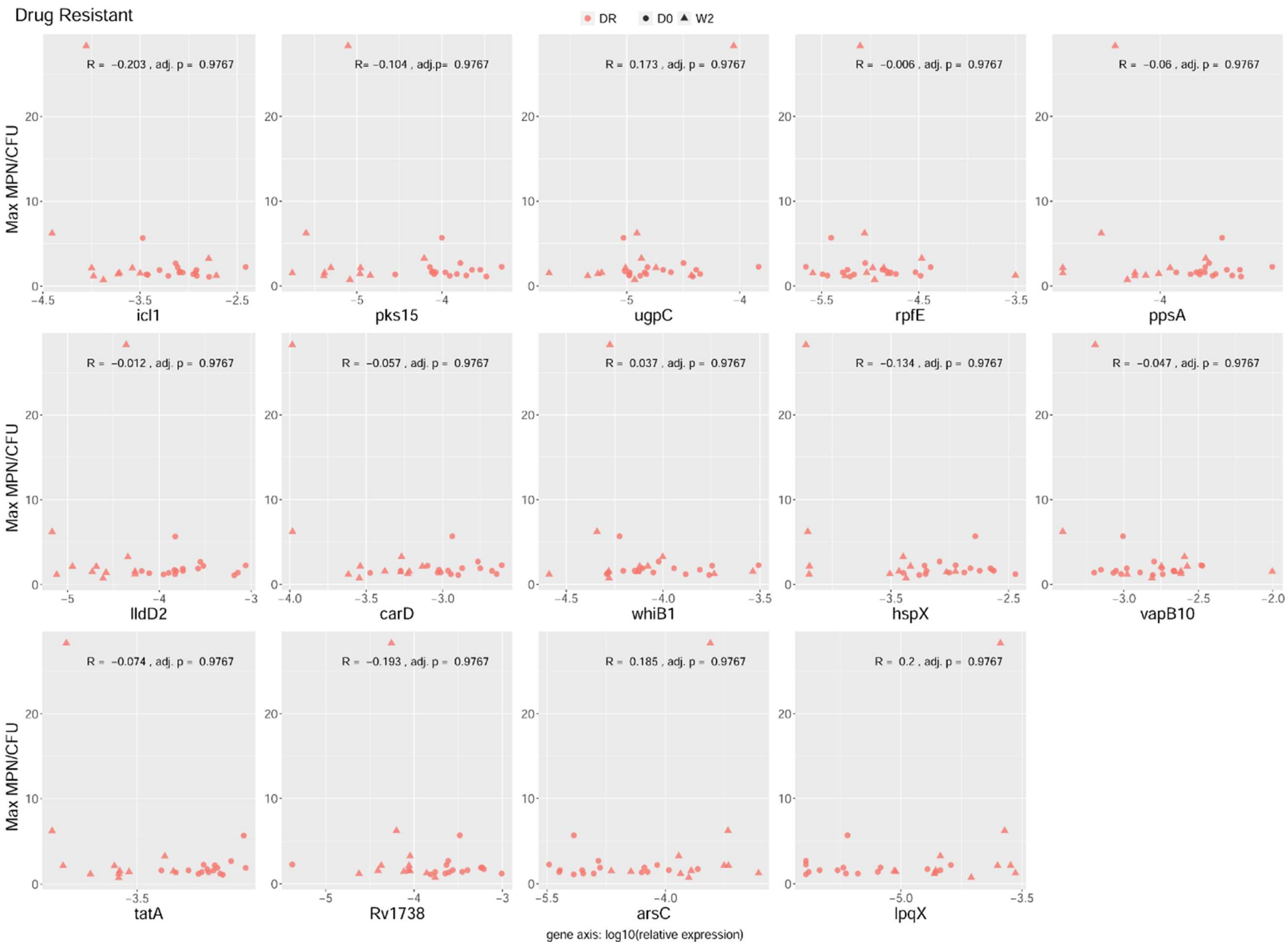
Drug Resistant
DR D0 W2
Max MPN/CFU
R = −0.203 , adj. p = 0.9767
icl1
R= −0.104 , adj.p= 0.9767
pks15
R = 0.173 , adj. p = 0.9767
ugpC
R = −0.006 , adj. p = 0.9767
rpfE
R = −0.06 , adj. p = 0.9767
ppsA
R = −0.012 , adj. p = 0.9767
lldD2
R = −0.057 , adj. p = 0.9767
carD
R = 0.037 , adj. p = 0.9767
whiB1
R = −0.134 , adj. p = 0.9767
hspX
R = −0.047 , adj. p = 0.9767
vapB10
R = −0.074 , adj. p = 0.9767
tatA
R = −0.193 , adj. p = 0.9767
Rv1738
R = 0.185 , adj. p = 0.9767
arsC
R = 0.2 , adj. p = 0.9767
lpqX
gene axis: log10(relative expression)

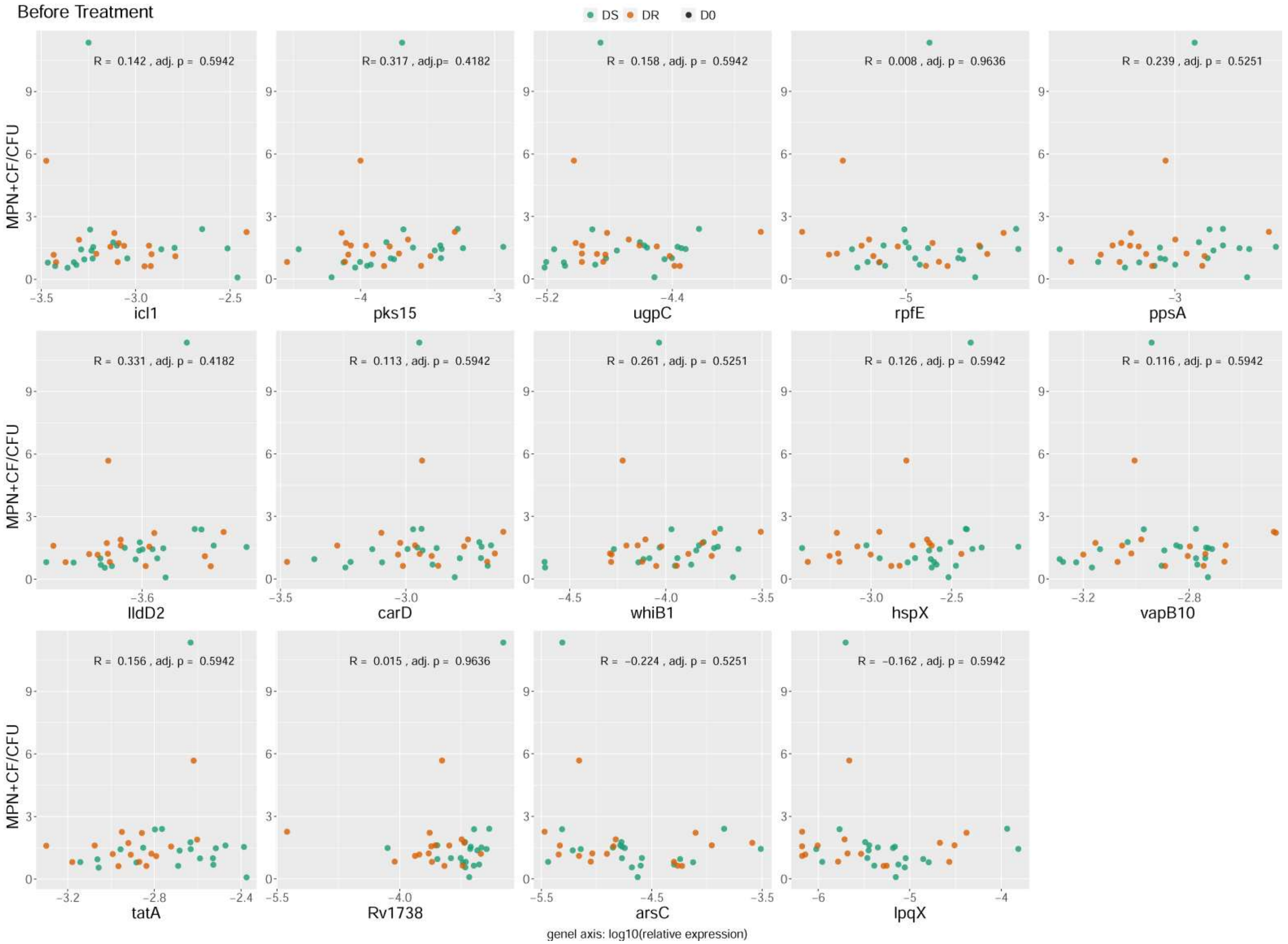

Before Treatment
DS DR D0
MPN+CF/CFU
R = 0.142 , adj. p = 0.5942
icl1
R= 0.317 , adj.p= 0.4182
pks15
R = 0.158 , adj. p = 0.5942
ugpC
R = 0.008 , adj. p = 0.9636
rpfE
R = 0.239 , adj. p = 0.5251
ppsA
R = 0.331 , adj. p = 0.4182
lldD2
R = 0.113 , adj. p = 0.5942
carD
R = 0.261 , adj. p = 0.5251
whiB1
R = 0.126 , adj. p = 0.5942
hspX
R = 0.116 , adj. p = 0.5942
vapB10
R = 0.156 , adj. p = 0.5942
tatA
R = 0.015 , adj. p = 0.9636
Rv1738
R = −0.224 , adj. p = 0.5251
arsC
R = −0.162 , adj. p = 0.5942
lpqX
genel axis: log10(relative expression)

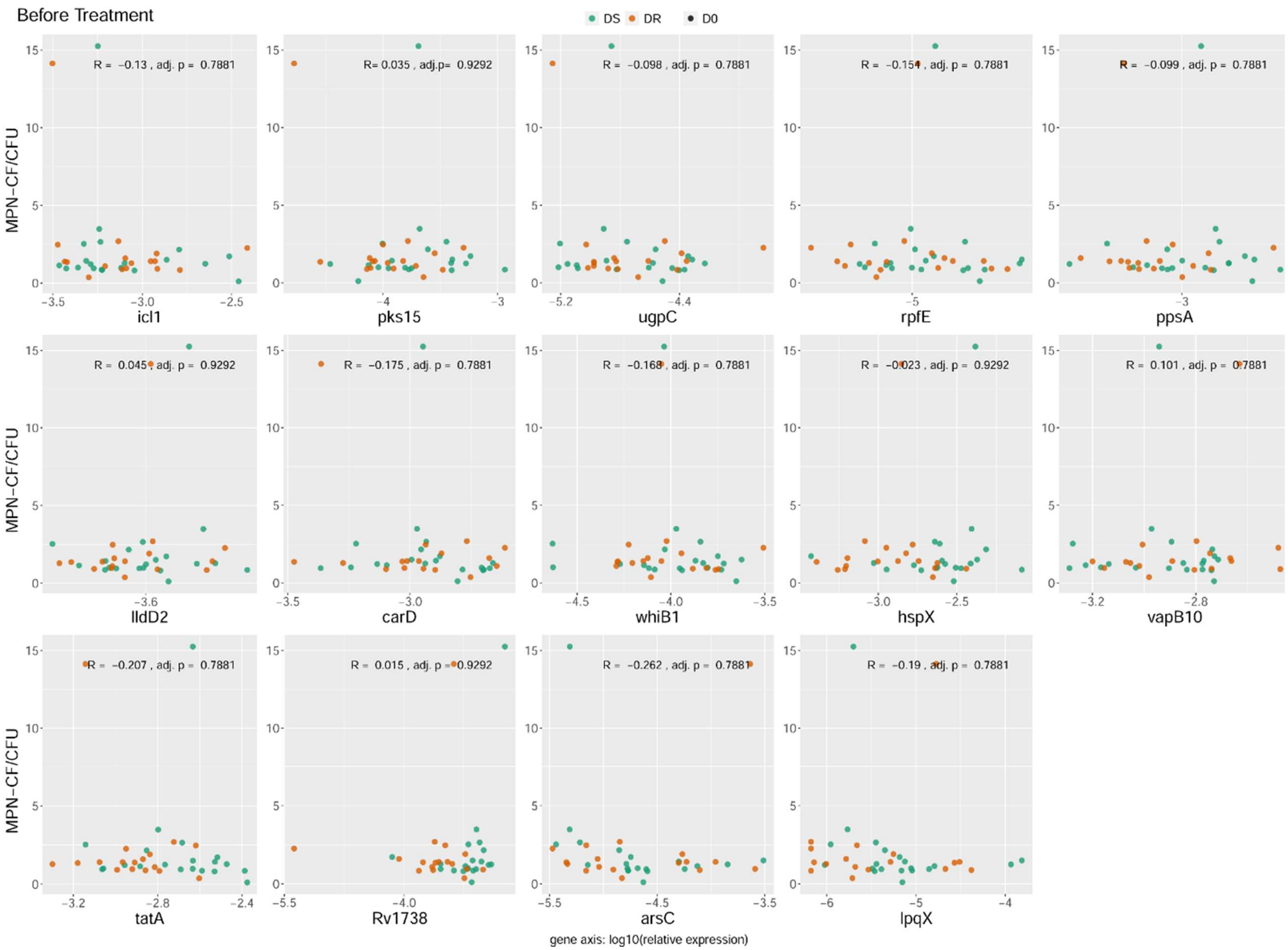

Before Treatment
DS DR D0
MPN-CF/CFU
R = −0.13 , adj. p = 0.7881
icl1
R= 0.035 , adj.p= 0.9292
pks15
R = −0.098 , adj. p = 0.7881
ugpC
R = −0.154 , adj. p = 0.7881
rpfE
R = −0.099 , adj. p = 0.7881
ppsA
R = 0.045 , adj. p = 0.9292
lldD2
R = −0.175 , adj. p = 0.7881
carD
R = −0.168 , adj. p = 0.7881
whiB1
R = −0.023 , adj. p = 0.9292
hspX
R = 0.101 , adj. p = 0.7881
vapB10
R = −0.207 , adj. p = 0.7881
tatA
R = 0.015 , adj. p = 0.9292
Rv1738
R = −0.262 , adj. p = 0.7881
arsC
R = −0.19 , adj. p = 0.7881
lpqX
gene axis: log10(relative expression)

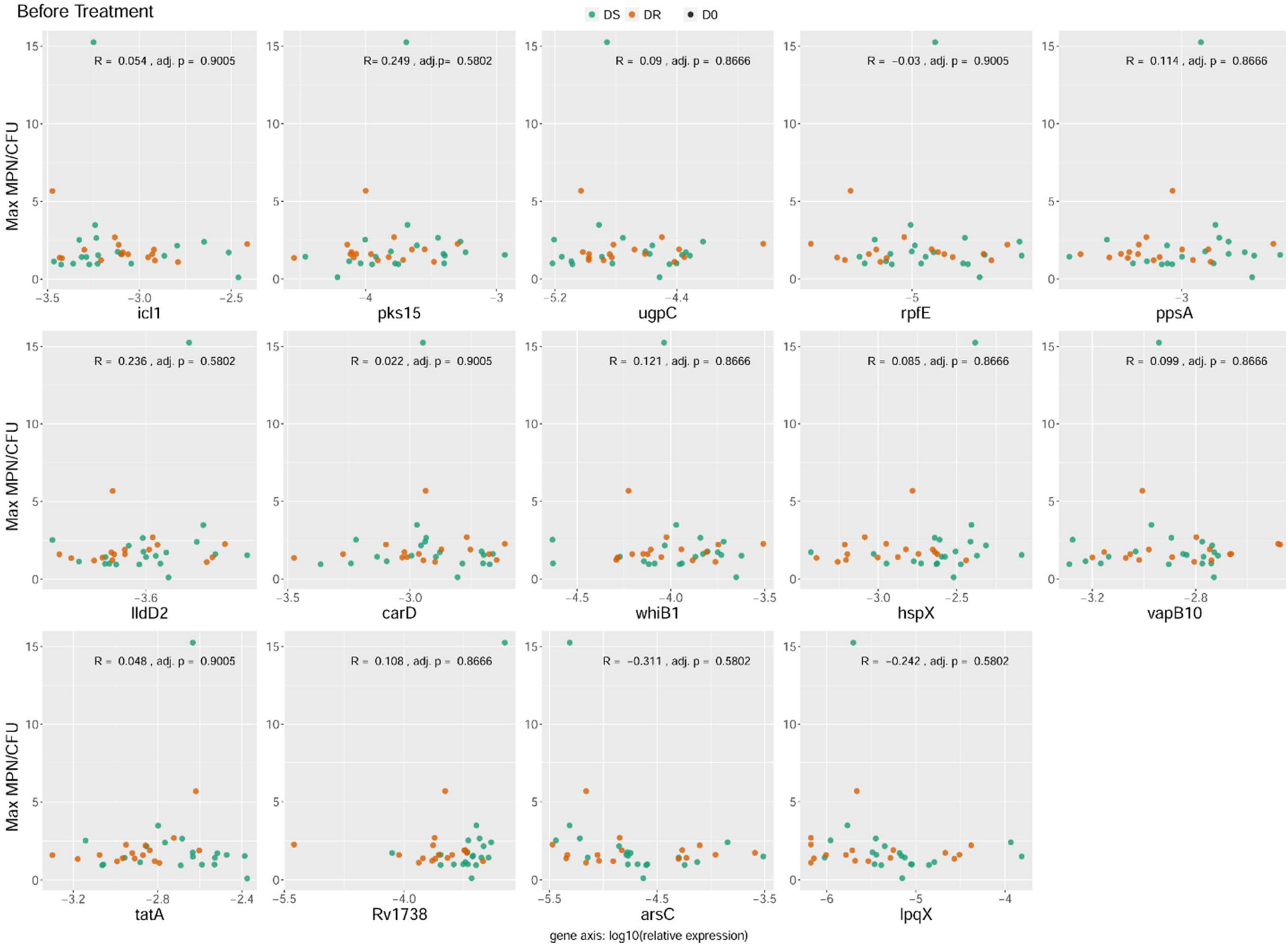

Before Treatment
DS DR D0
Max MPN/CFU
R = 0.054 , adj. p = 0.9005
icl1
R= 0.249 , adj.p= 0.5802
pks15
R = 0.09 , adj. p = 0.8666
ugpC
R = −0.03 , adj. p = 0.9005
rpfE
R = 0.114 , adj. p = 0.8666
ppsA
R = 0.236 , adj. p = 0.5802
lldD2
R = 0.022 , adj. p = 0.9005
carD
R = 0.121 , adj. p = 0.8666
whiB1
R = 0.085 , adj. p = 0.8666
hspX
R = 0.099 , adj. p = 0.8666
vapB10
R = 0.048 , adj. p = 0.9005
tatA
R = 0.108 , adj. p = 0.8666
Rv1738
R = −0.311 , adj. p = 0.5802
arsC
R = −0.242 , adj. p = 0.5802
lpqX
gene axis: log10(relative expression)

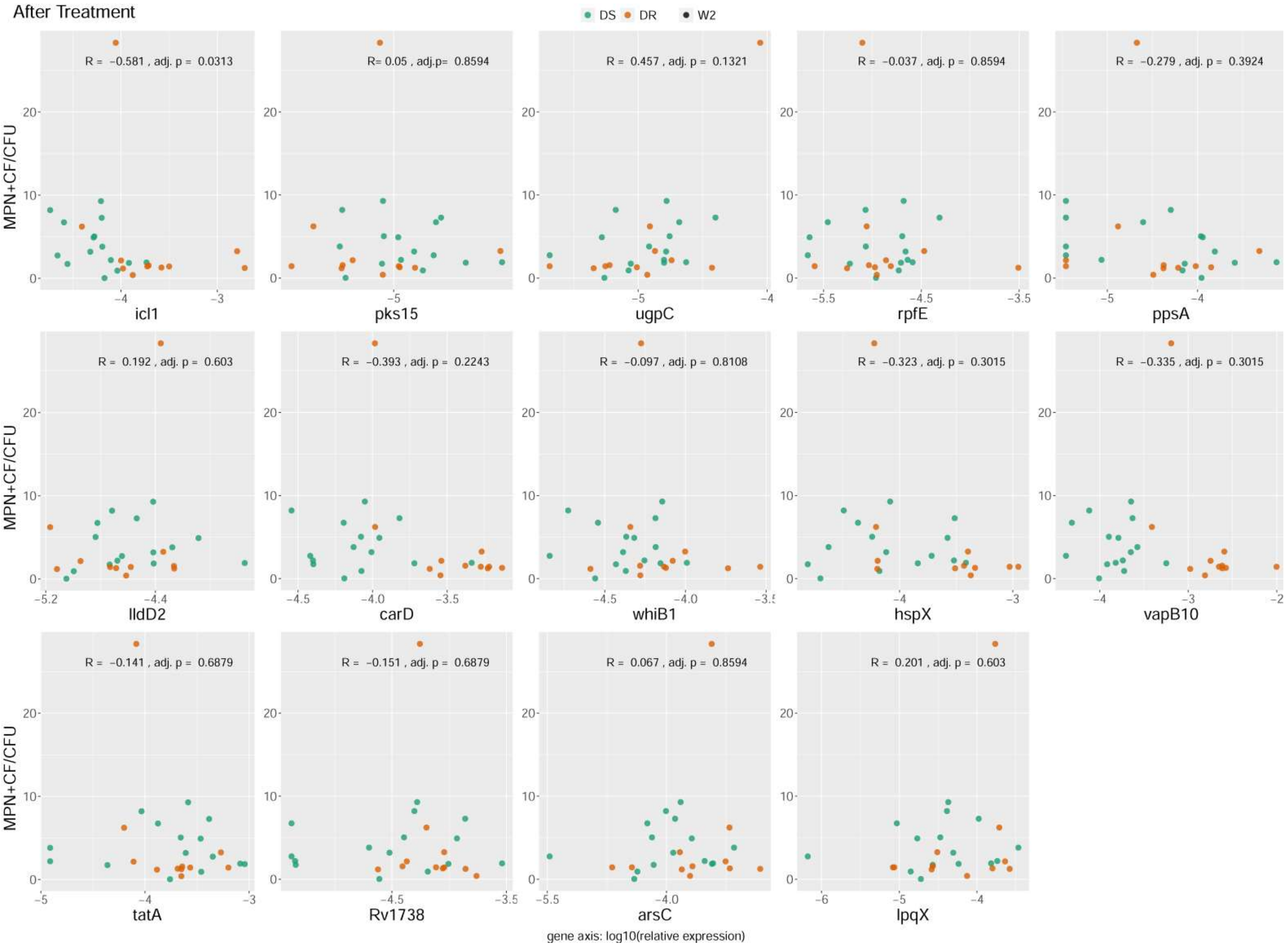
After Treatment
DS
DR
W2
MPN+CF/CFU
R = −0.581 , adj. p = 0.0313
icl1
R= 0.05 , adj.p= 0.8594
pks15
R = 0.457 , adj. p = 0.1321
ugpC
R = −0.037 , adj. p = 0.8594
rpfE
R = −0.279 , adj. p = 0.3924
ppsA
R = 0.192 , adj. p = 0.603
lldD2
R = −0.393 , adj. p = 0.2243
carD
R = −0.097 , adj. p = 0.8108
whiB1
R = −0.323 , adj. p = 0.3015
hspX
R = −0.335 , adj. p = 0.3015
vapB10
R = −0.141 , adj. p = 0.6879
tatA
R = −0.151 , adj. p = 0.6879
Rv1738
R = 0.067 , adj. p = 0.8594
arsC
R = 0.201 , adj. p = 0.603
lpqX
gene axis: log10(relative expression)

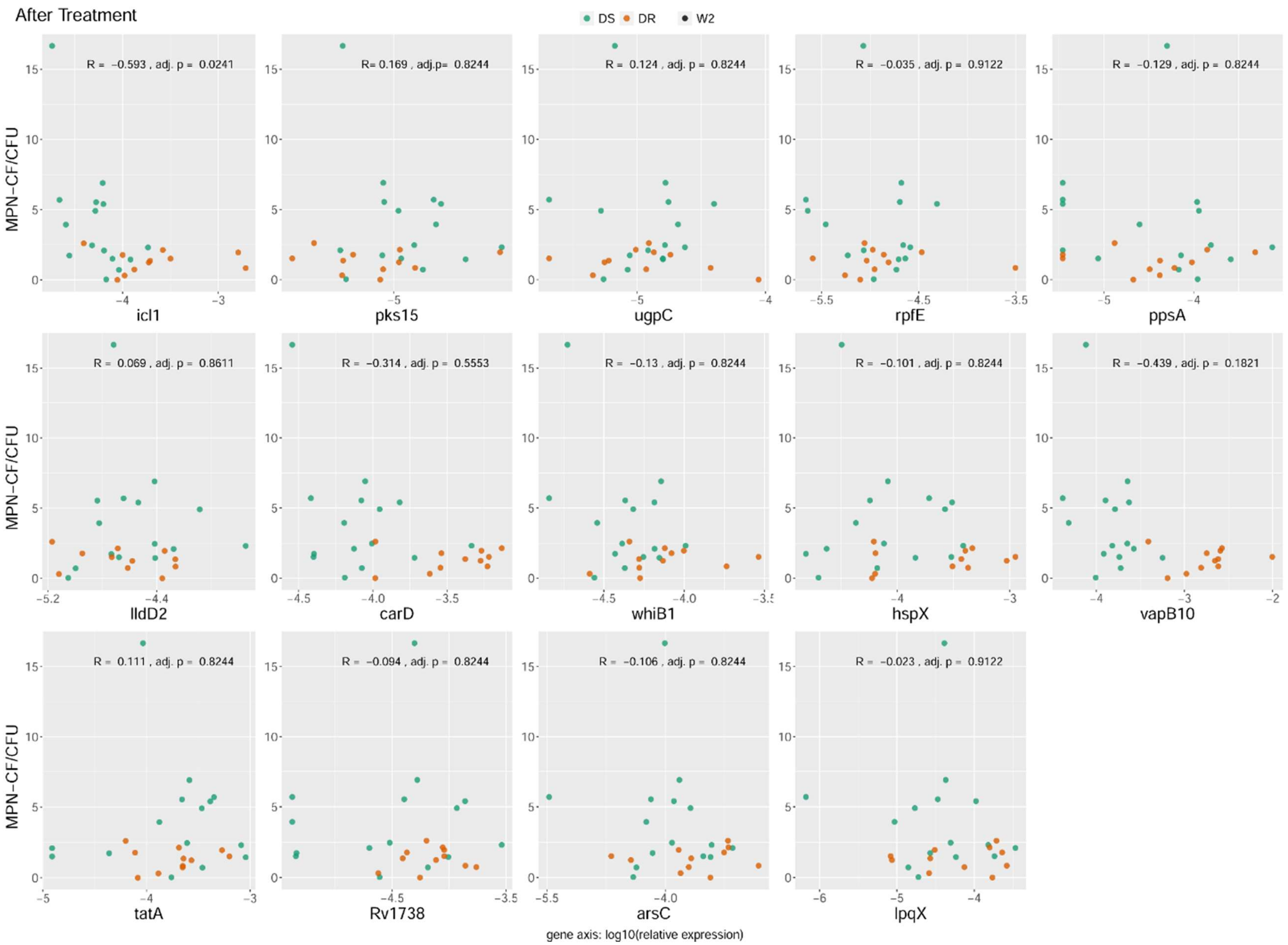
After Treatment
DS
DR
W2
MPN−CF/CFU
R = −0.593 , adj. p = 0.0241
icl1
R= 0.169 , adj.p= 0.8244
pks15
R = 0.124 , adj. p = 0.8244
ugpC
R = −0.035 , adj. p = 0.9122
rpfE
R = −0.129 , adj. p = 0.8244
ppsA
R = 0.069 , adj. p = 0.8611
lldD2
R = −0.314 , adj. p = 0.5553
carD
R = −0.13 , adj. p = 0.8244
whiB1
R = −0.101 , adj. p = 0.8244
hspX
R = −0.439 , adj. p = 0.1821
vapB10
R = 0.111 , adj. p = 0.8244
tatA
R = −0.094 , adj. p = 0.8244
Rv1738
R = −0.106 , adj. p = 0.8244
arsC
R = −0.023 , adj. p = 0.9122
lpqX
gene axis: log10(relative expression)

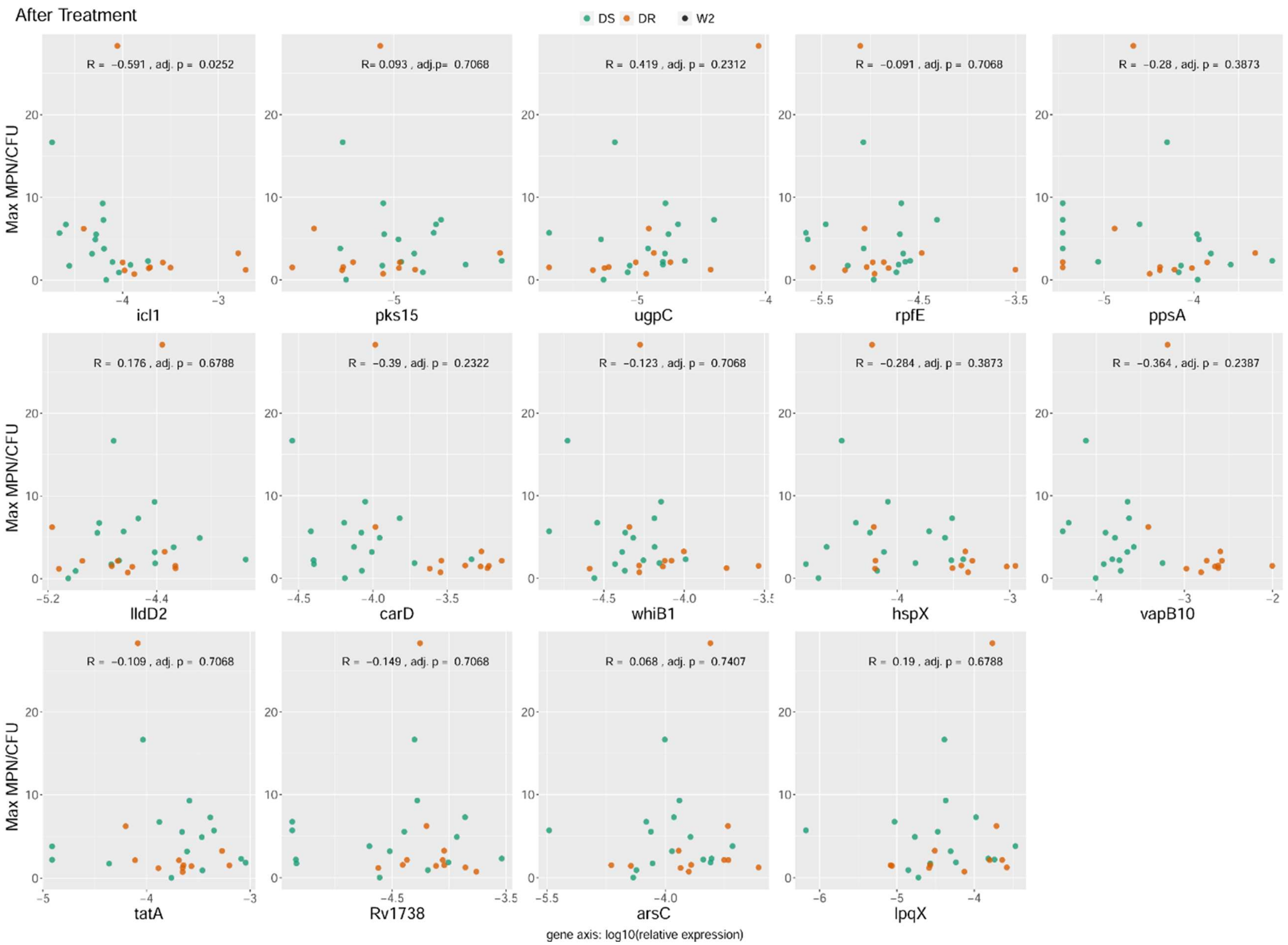
After Treatment
DS
DR
W2
Max MPN/CFU
R = −0.591 , adj. p = 0.0252
icl1
R= 0.093 , adj.p= 0.7068
pks15
R = 0.419 , adj. p = 0.2312
ugpC
R = −0.091 , adj. p = 0.7068
rpfE
R = −0.28 , adj. p = 0.3873
ppsA
R = 0.176 , adj. p = 0.6788
lldD2
R = −0.39 , adj. p = 0.2322
carD
R = −0.123 , adj. p = 0.7068
whiB1
R = −0.284 , adj. p = 0.3873
hspX
R = −0.364 , adj. p = 0.2387
vapB10
R = −0.109 , adj. p = 0.7068
tatA
R = −0.149 , adj. p = 0.7068
Rv1738
R = 0.068 , adj. p = 0.7407
arsC
R = 0.19 , adj. p = 0.6788
lpqX
gene axis: log10(relative expression)
