# Supplementary material for: Transcriptional Biomarkers of Differentially Detectable Mycobacterium tuberculosis in Patient Sputum
Source: mBio. 2022 Nov 3;13(6):e02701-22. doi: 10.1128/mbio.02701-22 (PMC9765512; doi:10.1128/mbio.02701-22)
Supplement: TABLE S4 [file mbio.02701-22-s0009.docx]

**∆GENE EXPRESSION vs ∆DDMtb FOR ALL PAIRED SAMPLES**

|  |  |  | **MPN^Max^/CFU**  **(n=21)** | | **MPN^+CF^/CFU**  **(n=21)** | | **MPN^-CF^/CFU**  **(n=22)** | |
| --- | --- | --- | --- | --- | --- | --- | --- | --- |
|  | **Gene** | **Rv#** | **Spearman correlation** | **p-val** | **Spearman correlation** | **p-val** | **Spearman correlation** | **p-val** |
| **Downregulated**  **DD Mtb candidates** | **icl1** | **Rv0467** | -0.506 | 0.0205 | -0.503 | 0.0216 | -0.493 | 0.021 |
|  | **carD** | **Rv3583c** | -0.413 | 0.064 | -0.457 | 0.0386 | -0.414 | 0.0567 |
|  | **vapB10** | **Rv1398c** | -0.404 | 0.0705 | -0.423 | 0.0571 | -0.348 | 0.1125 |
|  | **ppsA** | **Rv2931** | -0.392 | 0.0797 | -0.482 | 0.0284 | -0.303 | 0.1698 |
|  | **hspX** | **Rv2031c** | -0.343 | 0.1284 | -0.360 | 0.1098 | -0.370 | 0.091 |
|  | **Rv1738** | **Rv1738** | -0.203 | 0.3768 | -0.305 | 0.1782 | -0.255 | 0.2516 |
|  | **tatA** | **Rv2094c** | -0.005 | 0.9842 | -0.006 | 0.9797 | 0.048 | 0.8325 |
|  | **whiB1** | **Rv3219** | -0.132 | 0.5657 | -0.175 | 0.4454 | -0.123 | 0.5856 |
|  | **pks15** | **Rv2947c** | -0.160 | 0.4875 | -0.168 | 0.4662 | -0.243 | 0.2738 |
|  | **lldD2** | **Rv1872c** | -0.155 | 0.5019 | -0.110 | 0.6328 | -0.197 | 0.3777 |
| **Upregulated DD Mtb candidates** | **arsC** | **Rv2643** | -0.261 | 0.252 | -0.227 | 0.3203 | -0.188 | 0.4003 |
|  | **lpqX** | **Rv1228** | -0.232 | 0.3091 | -0.229 | 0.3175 | -0.265 | 0.2327 |
|  | **ugpC** | **Rv2832c** | 0.327 | 0.1476 | 0.340 | 0.1315 | 0.101 | 0.6535 |
|  | **rpfE** | **Rv2450c** | -0.135 | 0.558 | -0.155 | 0.5019 | -0.097 | 0.6682 |

**∆GENE EXPRESSION vs ∆DDMtb FOR DS PAIRED SAMPLES**

|  |  |  | **MPN^Max^/CFU**  **(n=13)** | | **MPN^+CF^/CFU**  **(n=13)** | | **MPN^-CF^/CFU**  **(n=13)** | |
| --- | --- | --- | --- | --- | --- | --- | --- | --- |
|  | **Gene** | **Rv#** | **Spearman correlation** | **p-val** | **Spearman correlation** | **p-val** | **Spearman correlation** | **p-val** |
| **Downregulated**  **DD Mtb candidates** | **icl1** | **Rv0467** | 0.198 | 0.5172 | 0.192 | 0.5292 | 0.148 | 0.6298 |
|  | **carD** | **Rv3583c** | 0.313 | 0.2973 | 0.253 | 0.4043 | 0.209 | 0.4935 |
|  | **vapB10** | **Rv1398c** | 0.291 | 0.3341 | 0.374 | 0.2094 | 0.291 | 0.3341 |
|  | **ppsA** | **Rv2931** | -0.121 | 0.6961 | -0.214 | 0.4819 | -0.060 | 0.849 |
|  | **hspX** | **Rv2031c** | 0.176 | 0.566 | 0.132 | 0.6693 | 0.110 | 0.7232 |
|  | **Rv1738** | **Rv1738** | 0.297 | 0.3247 | 0.159 | 0.6039 | 0.302 | 0.3154 |
|  | **tatA** | **Rv2094c** | 0.352 | 0.2392 | 0.286 | 0.3436 | 0.357 | 0.2315 |
|  | **whiB1** | **Rv3219** | 0.610 | 0.0303 | 0.610 | 0.0303 | 0.599 | 0.034 |
|  | **pks15** | **Rv2947c** | 0.099 | 0.7507 | 0.137 | 0.656 | 0.088 | 0.7785 |
|  | **lldD2** | **Rv1872c** | 0.269 | 0.3733 | 0.187 | 0.5413 | 0.209 | 0.4935 |
| **Upregulated DD Mtb candidates** | **arsC** | **Rv2643** | 0.055 | 0.8632 | 0.264 | 0.3835 | -0.060 | 0.849 |
|  | **lpqX** | **Rv1228** | 0.016 | 0.9639 | 0.187 | 0.5413 | 0.049 | 0.8775 |
|  | **ugpC** | **Rv2832c** | 0.610 | 0.0303 | 0.670 | 0.0149 | 0.516 | 0.074 |
|  | **rpfE** | **Rv2450c** | 0.390 | 0.1888 | 0.401 | 0.1758 | 0.401 | 0.1758 |

**∆GENE EXPRESSION vs ∆DDMtb FOR DR PAIRED SAMPLES**

|  |  |  | **MPN^Max^/CFU**  **(n=8)** | | **MPN^+CF^/CFU**  **(n=8)** | | **MPN^-CF^/CFU**  **(n=9)** | |
| --- | --- | --- | --- | --- | --- | --- | --- | --- |
|  | **Gene** | **Rv#** | **Spearman correlation** | **p-val** | **Spearman correlation** | **p-val** | **Spearman correlation** | **p-val** |
| **Downregulated**  **DD Mtb candidates** | **icl1** | **Rv0467** | -0.452 | 0.2675 | -0.524 | 0.1966 | -0.283 | 0.463 |
|  | **carD** | **Rv3583c** | -0.262 | 0.5364 | -0.452 | 0.2675 | -0.217 | 0.5809 |
|  | **vapB10** | **Rv1398c** | 0.000 | 1 | -0.357 | 0.3894 | 0.233 | 0.5517 |
|  | **ppsA** | **Rv2931** | -0.381 | 0.3599 | -0.500 | 0.2162 | -0.200 | 0.6134 |
|  | **hspX** | **Rv2031c** | 0.024 | 0.9768 | -0.143 | 0.752 | -0.267 | 0.4933 |
|  | **Rv1738** | **Rv1738** | 0.048 | 0.9349 | 0.024 | 0.9768 | -0.367 | 0.3363 |
|  | **tatA** | **Rv2094c** | -0.381 | 0.3599 | -0.310 | 0.4618 | -0.400 | 0.2912 |
|  | **whiB1** | **Rv3219** | -0.286 | 0.5008 | -0.310 | 0.4618 | -0.350 | 0.3586 |
|  | **pks15** | **Rv2947c** | -0.429 | 0.2992 | -0.500 | 0.2162 | -0.533 | 0.1475 |
|  | **lldD2** | **Rv1872c** | -0.333 | 0.4279 | -0.095 | 0.8401 | -0.650 | 0.0666 |
| **Upregulated DD Mtb candidates** | **arsC** | **Rv2643** | -0.333 | 0.4279 | -0.452 | 0.2675 | 0.117 | 0.7756 |
|  | **lpqX** | **Rv1228** | -0.095 | 0.8401 | -0.214 | 0.6191 | 0.100 | 0.81 |
|  | **ugpC** | **Rv2832c** | -0.119 | 0.793 | -0.048 | 0.9349 | -0.467 | 0.2125 |
|  | **rpfE** | **Rv2450c** | -0.381 | 0.3599 | -0.405 | 0.3268 | -0.233 | 0.5517 |
