# Supplementary material for: Transcriptional Biomarkers of Differentially Detectable Mycobacterium tuberculosis in Patient Sputum
Source: mBio. 2022 Nov 3;13(6):e02701-22. doi: 10.1128/mbio.02701-22 (PMC9765512; doi:10.1128/mbio.02701-22)
Supplement: TABLE S5 [file mbio.02701-22-s0010.docx]

**D0 GENE EXPRESSION vs ∆DDMtb FOR ALL PAIRED SAMPLES**

|  |  |  | **MPN^Max^/CFU**  **(n=21)** | | **MPN^+CF^/CFU**  **(n=21)** | | **MPN^-CF^/CFU**  **(n=22)** | |
| --- | --- | --- | --- | --- | --- | --- | --- | --- |
|  | **Gene** | **Rv#** | **Spearman correlation** | **p-val** | **Spearman correlation** | **p-val** | **Spearman correlation** | **p-val** |
| **Downregulated**  **DD Mtb candidates** | **icl1** | **Rv0467** | -0.045 | 0.8456 | -0.023 | 0.9213 | -0.049 | 0.8286 |
|  | **carD** | **Rv3583c** | -0.305 | 0.1782 | -0.303 | 0.182 | -0.081 | 0.7205 |
|  | **vapB10** | **Rv1398c** | -0.416 | 0.0622 | -0.290 | 0.2023 | -0.582 | 0.0052 |
|  | **ppsA** | **Rv2931** | -0.123 | 0.5929 | -0.175 | 0.4454 | 0.025 | 0.9118 |
|  | **hspX** | **Rv2031c** | 0.282 | 0.2151 | 0.310 | 0.1706 | 0.281 | 0.2051 |
|  | **Rv1738** | **Rv1738** | 0.396 | 0.0765 | 0.490 | 0.0257 | 0.361 | 0.0996 |
|  | **tatA** | **Rv2094c** | -0.242 | 0.2901 | -0.240 | 0.2928 | 0.019 | 0.9358 |
|  | **whiB1** | **Rv3219** | -0.042 | 0.8589 | 0.025 | 0.9169 | -0.130 | 0.5614 |
|  | **pks15** | **Rv2947c** | -0.005 | 0.9842 | 0.026 | 0.9124 | 0.119 | 0.5962 |
|  | **lldD2** | **Rv1872c** | -0.105 | 0.6491 | -0.056 | 0.8104 | -0.184 | 0.4119 |
| **Upregulated DD Mtb candidates** | **arsC** | **Rv2643** | 0.073 | 0.754 | 0.077 | 0.7411 | -0.077 | 0.7318 |
|  | **lpqX** | **Rv1228** | 0.205 | 0.3706 | 0.175 | 0.4454 | 0.023 | 0.9198 |
|  | **ugpC** | **Rv2832c** | -0.322 | 0.1544 | -0.257 | 0.2593 | -0.214 | 0.3373 |
|  | **rpfE** | **Rv2450c** | 0.009 | 0.9707 | 0.106 | 0.645 | -0.089 | 0.6942 |

**D0 GENE EXPRESSION vs ∆DDMtb FOR DS PAIRED SAMPLES**

|  |  |  | **MPN^Max^/CFU**  **(n=13)** | | **MPN^+CF^/CFU**  **(n=13)** | | **MPN^-CF^/CFU**  **(n=13)** | |
| --- | --- | --- | --- | --- | --- | --- | --- | --- |
|  | **Gene** | **Rv#** | **Spearman correlation** | **p-val** | **Spearman correlation** | **p-val** | **Spearman correlation** | **p-val** |
| **Downregulated**  **DD Mtb candidates** | **icl1** | **Rv0467** | -0.687 | 0.012 | -0.703 | 0.0096 | -0.703 | 0.0096 |
|  | **carD** | **Rv3583c** | -0.462 | 0.1149 | -0.379 | 0.2024 | -0.379 | 0.2024 |
|  | **vapB10** | **Rv1398c** | -0.764 | 0.0036 | -0.797 | 0.0018 | -0.775 | 0.0029 |
|  | **ppsA** | **Rv2931** | -0.538 | 0.0611 | -0.615 | 0.0285 | -0.555 | 0.0525 |
|  | **hspX** | **Rv2031c** | -0.489 | 0.0929 | -0.500 | 0.085 | -0.527 | 0.0673 |
|  | **Rv1738** | **Rv1738** | -0.242 | 0.4258 | -0.110 | 0.7232 | -0.385 | 0.1955 |
|  | **tatA** | **Rv2094c** | -0.599 | 0.034 | -0.560 | 0.0499 | -0.527 | 0.0673 |
|  | **whiB1** | **Rv3219** | -0.626 | 0.0253 | -0.687 | 0.012 | -0.687 | 0.012 |
|  | **pks15** | **Rv2947c** | -0.280 | 0.3534 | -0.335 | 0.2631 | -0.330 | 0.2715 |
|  | **lldD2** | **Rv1872c** | -0.379 | 0.2024 | -0.407 | 0.1695 | -0.357 | 0.2315 |
| **Upregulated DD Mtb candidates** | **arsC** | **Rv2643** | -0.319 | 0.2886 | -0.473 | 0.1057 | -0.319 | 0.2886 |
|  | **lpqX** | **Rv1228** | -0.022 | 0.9494 | -0.203 | 0.5053 | -0.115 | 0.7096 |
|  | **ugpC** | **Rv2832c** | -0.593 | 0.036 | -0.665 | 0.016 | -0.621 | 0.0269 |
|  | **rpfE** | **Rv2450c** | -0.555 | 0.0525 | -0.643 | 0.0209 | -0.599 | 0.034 |

**D0 GENE EXPRESSION vs ∆DDMtb FOR DR PAIRED SAMPLES**

|  |  |  | **MPN^Max^/CFU**  **(n=8)** | | **MPN^+CF^/CFU**  **(n=8)** | | **MPN^-CF^/CFU**  **(n=9)** | |
| --- | --- | --- | --- | --- | --- | --- | --- | --- |
|  | **Gene** | **Rv#** | **Spearman correlation** | **p-val** | **Spearman correlation** | **p-val** | **Spearman correlation** | **p-val** |
| **Downregulated**  **DD Mtb candidates** | **icl1** | **Rv0467** | 0.683 | 0.0503 | 0.867 | 0.0045 | 0.350 | 0.3586 |
|  | **carD** | **Rv3583c** | 0.283 | 0.463 | 0.250 | 0.5206 | 0.333 | 0.3853 |
|  | **vapB10** | **Rv1398c** | -0.617 | 0.0857 | -0.333 | 0.3853 | -0.667 | 0.0589 |
|  | **ppsA** | **Rv2931** | 0.150 | 0.7081 | 0.050 | 0.9116 | 0.467 | 0.2125 |
|  | **hspX** | **Rv2031c** | -0.033 | 0.9484 | 0.100 | 0.81 | 0.417 | 0.2696 |
|  | **Rv1738** | **Rv1738** | 0.000 | 1 | 0.117 | 0.7756 | 0.300 | 0.4366 |
|  | **tatA** | **Rv2094c** | -0.067 | 0.8801 | -0.117 | 0.7756 | 0.400 | 0.2912 |
|  | **whiB1** | **Rv3219** | -0.017 | 0.9816 | 0.317 | 0.4101 | -0.017 | 0.9816 |
|  | **pks15** | **Rv2947c** | 0.267 | 0.4933 | 0.233 | 0.5517 | 0.533 | 0.1475 |
|  | **lldD2** | **Rv1872c** | -0.500 | 0.1777 | -0.267 | 0.4933 | -0.400 | 0.2912 |
| **Upregulated DD Mtb candidates** | **arsC** | **Rv2643** | 0.050 | 0.9116 | 0.167 | 0.6777 | -0.017 | 0.9816 |
|  | **lpqX** | **Rv1228** | -0.100 | 0.81 | -0.050 | 0.9116 | -0.117 | 0.7756 |
|  | **ugpC** | **Rv2832c** | 0.267 | 0.4933 | 0.450 | 0.2298 | 0.100 | 0.81 |
|  | **rpfE** | **Rv2450c** | 0.017 | 0.9816 | 0.433 | 0.2499 | -0.350 | 0.3586 |
